# Generalized Score Matching for Non-Negative Data

**Published:** 2019-04

**Authors:** Shiqing Yu, Mathias Drton, Ali Shojaie

**Affiliations:** Department of Statistics, University of Washington, Seattle, WA, U.S.A.; Department of Mathematical Sciences, University of Copenhagen, Copenhagen, Denmark; Department of Statistics, University of Washington, Seattle, WA, U.S.A.; Department of Biostatistics, University of Washington, Seattle, WA, U.S.A.

**Keywords:** exponential family, graphical model, positive data, score matching, sparsity

## Abstract

A common challenge in estimating parameters of probability density functions is the intractability of the normalizing constant. While in such cases maximum likelihood estimation may be implemented using numerical integration, the approach becomes computationally intensive. The score matching method of Hyvärinen (2005) avoids direct calculation of the normalizing constant and yields closed-form estimates for exponential families of continuous distributions over $R^{m}$. Hyvärinen (2007) extended the approach to distributions supported on the non-negative orthant, $R_{+}^{m}$. In this paper, we give a generalized form of score matching for non-negative data that improves estimation efficiency. As an example, we consider a general class of pairwise interaction models. Addressing an overlooked inexistence problem, we generalize the regularized score matching method of Lin et al. (2016) and improve its theoretical guarantees for non-negative Gaussian graphical models.

## 1. Introduction

Score matching was first developed in Hyvärinen (2005) for continuous distributions supported on all of $R^{m}$. Consider such a distribution *P*_0_, with density *p*_0_ and support equal to $R^{m}$. Let P be a family of distributions with twice continuously differentiable densities. The score matching estimator of *p*_0_ using P as a model is the minimizer of the expected squared *ℓ*_2_ distance between the gradients of log *p*_0_ and a log-density from P. So we minimize the loss $\int_{R^{m}}p_{0}(x)\|\nabla \;\log\;p(x)-\nabla \;\log\;p_{0}(x){\|}_{2}^{2}\;dx$ with respect to densities *p* from P. The loss depends on *p*_0_, but integration by parts can be used to rewrite it in a form that can be approximated by averaging over the sample without knowing *p*_0_. A key feature of score matching is that normalizing constants cancel in gradients of log-densities, allowing for simple treatment of models with intractable normalizing constants. For exponential families, the loss is quadratic in the canonical parameter, making optimization straightforward.

If the considered distributions are supported on a proper subset of $R^{m}$, then the integration by parts arguments underlying the score matching estimator may fail due to discontinuities at the boundary of the support. For data supported on the non-negative orthant $R_{+}^{m}$, Hyvärinen (2007) addresses this problem by modifying the loss to $\int_{R^{m}}p_{0}(x)\|\nabla \;\log\;p(x)\circ x-\nabla \;\log\;p_{0}(x)\circ x{\|}_{2}^{2}\;dx$, where ○ denotes entrywise multiplication. In this loss, boundary effects are dampened by multiplying gradients elementwise with the identity functions *x_j_*.

In this paper, we propose *generalized score matching* methods that are based on elementwise multiplication with functions other than *x_j_*. As we show, this can lead to drastically improved estimation accuracy, both theoretically and empirically. To demonstrate these advantages, we consider a family of graphical models on $R_{+}^{m}$, which does not have tractable normalizing constants and hence serves as a practical example.

*Graphical models* specify conditional independence relations for a random vector ***X*** = (*X_i_*)_*i*∈*V*_ indexed by the nodes of a graph (Lauritzen, 1996). For undirected graphs, variables *X_i_* and *X_j_* are required to be conditionally independent given (*X_k_*)_*k*≠*i*,*j*_ if there is no edge between *i* and *j*. The smallest undirected graph with this property is the *conditional independence graph* of ***X***. Estimation of this graph and associated interaction parameters has been a topic of continued research as reviewed by Drton and Maathuis (2017).

Largely due to their tractability, Gaussian graphical models (GGMs) have gained great popularity. The conditional independence graph of a multivariate normal vector X∼N(μ,Σ) is determined by the *inverse covariance matrix* **K** ≡ **Σ**^−1^, also termed *concentration* or *precision matrix*. Specifically, *X_i_* and *X_j_* are conditionally independent given all other variables if and only if the (*i*, *j*)-th and the (*j*, *i*)-th entries of **K** are both zero. This simple relation underlies a rich literature including Drton and Perlman (2004), Meinshausen and Bühlmann (2006), Yuan and Lin (2007) and Friedman et al. (2008), among others.

More recent work has provided tractable procedures also for non-Gaussian graphical models. This includes Gaussian copula models (Liu et al., 2009; Dobra and Lenkoski, 2011; Liu et al., 2012), Ising models (Ravikumar et al., 2010), other exponential family models (Chen et al., 2015; Yang et al., 2015), as well as semi- or non-parametric estimation techniques (Fellinghauer et al., 2013; Voorman et al., 2014). In this paper, we apply our method to a class of pairwise interaction models that generalizes non-negative Gaussian random variables, as recently considered by Lin et al. (2016) and Yu et al. (2016), as well as square root graphical models proposed by Inouye et al. (2016) when the sufficient statistic function is a pure power. However, our main ideas can also be applied for other classes of exponential families whose support is restricted to a rectangular set.

Our focus will be on *pairwise interaction power models* with probability distributions having (Lebesgue) densities proportional to

(1) $$\text{exp}\left\{-\frac{1}{2a}x^{a⊤}Kx^{a}+\eta^{⊤}\frac{x^{b}-1_{m}}{b}\right\}$$

on $R_{+}^{m}\equiv [0,\infty {)}^{m}$. Here *a* > 0 and *b* ≥ 0 are known constants, and $K\in R^{m\times m}$ and $\eta \in R^{m}$ are unknown parameters of interest. When *b* = 0 we define (*x*^*b*^ – 1)/*b* ≡ log *x* and $R_{+}^{m}\equiv (0,\infty {)}^{m}$. This class of models is motivated by the form of important univariate distributions for non-negative data, including gamma and truncated normal distributions. It provides a framework for pairwise interaction that is concrete yet rich enough to capture key differences in how densities may behave at the boundary of the non-negative orthant, $R_{+}^{m}$. Moreover, the conditional independence graph of a random vector ***X*** with distribution as in (1) is determined just as in the Gaussian case: *X_i_* and *X_j_* are conditionally independent given all other variables if and only if *κ_ij_* = *κ_ji_* = 0 in the interaction matrix **K**. Section 5.1 gives further details on these models. We will develop estimators of (***η***, **K**) in (1) and the associated conditional independence graph using the proposed *generalized score matching*.

A special case of (1) are truncated Gaussian graphical models, with *a* = *b* = 1. Let $\mu \in R^{m}$, and let **K** be a positive definite matrix. Then a non-negative random vector ***X*** follows a truncated normal distribution for mean parameter ***μ*** and inverse covariance parameter **K**, in symbols ***X*** ~ TN(***μ***, **K**), if it has density proportional to

(2) $$\text{exp}\left\{-\frac{1}{2}(x-\mu {)}^{⊤}K(x-\mu )\right\}$$

on $R_{+}^{m}$. We refer to **Σ** = **K**^−1^ as the covariance parameter of the distribution, and note that the ***η*** parameter in (1) is **K*μ***. Another special case of (1) is the exponential square root graphical models in Inouye et al. (2016), where *a* = *b* = 1/2.

Lin et al. (2016) estimate truncated GGMs based on Hyvärinen’s modification, with an *ℓ*_1_ penalty on the entries of **K** added to the loss. However, the paper overlooks the fact that the loss can be unbounded from below in the high-dimensional setting even with an *ℓ*_1_ penalty, such that no minimizer may exist. Since the unpenalized loss is quadratic in the parameter to be estimated, we propose modifying it by adding small positive values to the diagonals of the positive semi-definite matrix that defines the quadratic part, in order to ensure that the loss is bounded and strongly convex and admits a unique minimizer. We apply this to the estimator for GGMs considered in Lin et al. (2016), which uses score-matching on $R^{m}$, and to the *generalized score matching* estimator for pairwise interaction power models on $R_{+}^{m}$ proposed in this paper. In these cases, we show, both empirically and theoretically, that the consistency results still hold (or even improve) if the positive values added are smaller than a threshold that is readily computable.

The rest of the paper is organized as follows. Section 2 introduces score matching and our proposed *generalized score matching*. In Section 3, we apply generalized score matching to exponential families, with univariate truncated normal distributions as an example. *Regularized generalized score matching* for graphical models is formulated in Section 4. The estimators for pairwise interaction power models are shown in Section 5, while theoretical consistency results are presented in Section 6, where we treat the probabilistically most tractable case of truncated GGMs. Simulation results and applications to RNAseq data are given in Section 7. Proofs for theorems in Section 2-6 are presented in Appendices A and B. Additional experimental results are presented in Appendix C.

### 1.1 Notation

Constant scalars, vectors, and functions are written in lower-case (e.g., *a*, ***a***), random scalars and vectors in upper-case (e.g., *X*, ***X***). Regular font is used for scalars (e.g. *a*, *X*), and boldface for vectors (e.g. ***a***, ***X***). Matrices are in upright bold, with constant matrices in upper-case (**K**, **M**) and random matrices holding observations in lower-case (**x**, **y**). Subscripts refer to entries in vectors and columns in matrices. Superscripts refer to rows in matrices. So *X_j_* is the *j*-th component of a random vector ***X***. For a data matrix $x\in R^{n\times m}$, each row comprising one observation of *m* variables/features, $X_{j}^{\left(i\right)}$ is the *j*-th feature for the *i*-th observation. Stacking the columns of a matrix $K=[\kappa_{ij}{]}_{i,j}\in R^{q\times r}$ gives its vectorization vec(**K**) = (*κ*_11_,…, *κ*_*q*1_, *κ*_12_,…, *κ*_*q*2_,…, *κ*_1*r*_,…, *κ_qr_*)^⊤^. For a matrix $K\in R^{q\times q}$, $\text{diag}(K)\in R^{q}$ denotes its diagonal, and for a vector $v\in R^{q}$, diag(***v***) is the *q* × *q* diagonal matrix with diagonals *v*_1_,… ,*v*_*q*_.

For *a* ≥ 1, the *ℓ*_*a*_-norm of a vector $v\in R^{q}$ is denoted

$$\|v{\|}_{a}={\left(\sum_{j=1}^{q}|v_{j}{|}^{a}\right)}^{1∕a},$$

with $\|v{\|}_{\infty }=\max_{j=1,\ldots ,q}|v_{j}|$. A matrix $K=[\kappa_{ij}{]}_{i,j}\in R^{q\times r}$ has Frobenius norm

$$\||K\|{|}_{F}\equiv \|\text{vec}(K){\|}_{2}\equiv \sqrt{\sum_{i=1}^{q}\sum_{j=1}^{r}\kappa_{ij}^{2}},$$

and max $\|K{\|}_{\infty }\equiv \|\text{vec}(K){\|}_{\infty }\equiv \max_{i,j}|\kappa_{ij}|$. Its *ℓ*_*a*_-*ℓ*_*b*_ operator norm is

$$\||K\|{|}_{a,b}\equiv \max_{x\neq 0}\frac{\|Kx{\|}_{b}}{\|x{\|}_{a}}$$

with shorthand notation ⫼**K**⫼_*a*_ ≡ ⫼**K**⫼_*a*,*a*_; for instance, $|\|K\|{|}_{\infty }\equiv \max_{i=1.\ldots ,q}\sum_{j=1}^{r}|\kappa_{ij}|$.

For a function $f\;:R^{m}\to R$, we define *∂_j_f*(***x***) as the partial derivative with respect to *x_j_*, and *∂_jj_f*(***x***) = *∂_j_∂_j_f*(***x***). For $f\;:R\to R^{m}$, ***f***(*x*) = (*f*_1_(*x*),…, *f_m_*(*x*))^⊤^, we let $f^{\prime }(x)=(f_{1}^{\prime }(x),\ldots ,f_{m}^{\prime }(x){)}^{⊤}$ be the vector of derivatives. Likewise **f″**(*x*) is used for second derivatives. The symbol $1_{A}(\cdot )$ denotes the indicator function of the set *A*, while $1_{n}\in R^{n}$ is the vector of all 1’s. For $a,b\in R^{m}$, ***a*** ○ ***b*** ≡ (*a*_1_*b*_1_,…, *a*_*m*_*b*_*m*_)^⊤^. A density of a distribution is always a probability density function with respect to Lebesgue measure. When it is clear from the context, $E_{0}$ denotes the expectation under a true distribution *P*_0_.

## 2. Score Matching

In this section, we review the original score matching and develop our generalized score matching estimators.

### 2.1. Original Score Matching

Let ***X*** be a random vector taking values in $R^{m}$ with distribution *P*_0_ and density *p*_0_. Let P be a family of distributions of interest with twice continuously differentiable densities supported on $R^{m}$. Suppose $P_{0}\in P$. The *score matching loss* for P∈P, with density *p*, is given by

(3) $$J(P)=\int_{R^{m}}p_{0}(x)\|\nabla \;\log\;p(x)-\nabla \;\log\;p_{0}(x){\|}_{2}^{2}\;dx.$$

The gradients in (3) can be thought of as gradients with respect to a hypothetical location parameter, evaluated at the origin (Hyvärinen, 2005). The loss *J*(*P*) is minimized if and only if *P* = *P*_0_, which forms the basis for estimation of *P*_0_. Importantly, since the loss depends on *p* only through its log-gradient, it suffices to know *p* up to a normalizing constant. Under mild conditions, (3) can be rewritten as

(4) $$J(P)=\int_{R^{m}}p_{0}(x)\sum_{j=1}^{m}\left[\partial_{jj}\;\log\;p(x)+\frac{(\partial_{j}\;\log\;p(x){)}^{2}}{2}\right]dx,$$

plus a constant independent of *p*. The integral in (4) can be approximated by a sample average; this alleviates the need for knowing the true density *p*_0_, and provides a way to estimate *p*_0_.

### 2.2. Generalized Score Matching for Non-Negative Data

When the true density *p*_0_ is supported on a proper subset of $R^{m}$, the integration by parts underlying the equivalence of (3) and (4) may fail due to discontinuity at the boundary. For distributions supported on the non-negative orthant, $R_{+}^{m}$, Hyvärinen (2007) addressed this issue by instead minimizing the *non-negative score matching loss*

(5) $$J_{+}(P)=\int_{R_{+}^{m}}p_{0}(x)\|\nabla \;\log\;p(x)\circ x-\nabla \;\log\;p_{0}(x)\circ x{\|}_{2}^{2}\;dx.$$

This loss can be motivated via gradients with respect to a hypothetical scale parameter (Hyvärinen, 2007). Under mild conditions, *J*_+_(*P*) can again be rewritten in terms of an expectation of a function independent of *p*_0_, thus allowing one to form a sample loss.

In this work, we consider generalizing the non-negative score matching loss as follows.

**Definition 1** *Let* $P_{+}$ *be the family of distributions of interest, and assume every* $P\in P_{+}$ *has a twice continuously differentiable density supported on* $R_{+}^{m}$. *Suppose the m-variate random vector **X** has true distribution* $P_{0}\in P_{+}$, *and let p*_0_ *be its twice continuously differentiable density. Let h*_1_,…, *h*_*m*_ : $R_{+}\to R_{+}$ *be a.s. positive functions that are absolutely continuous in every bounded sub-interval of* $R_{+}$, *and set **h***(***x***) = (*h*_1_(*x*_1_),… ,*h_m_*(*x_m_*))^⊤^. *For* $P\in P_{+}$ *with density p, the* generalized ***h***-score matching loss *is*

(6) $$J_{h}(P)=\int_{R_{+}^{m}}\frac{1}{2}p_{0}(x)\|\nabla \;\log\;p(x)\circ h(x{)}^{1∕2}-\nabla \;\log\;p_{0}(x)\circ h(x{)}^{1∕2}{\|}_{2}^{2}\;dx,$$

*where* $h^{1∕2}(x)\equiv (h_{1}^{1∕2}(x_{1}),\ldots ,h_{m}^{1∕2}(x_{m}){)}^{⊤}$.

**Proposition 2** *The distribution P*_0_ *is the unique minimizer of J_**h**_*(*P*) *for* $P\in P_{+}$.

**Proof** First, observe that *J*_***h***_(*P*) ≥ 0 and *J_**h**_*(*P*_0_) = 0. For uniqueness, suppose *J*_***h***_(*P*_1_) = 0 for some $P_{1}\in P_{+}$. Let *p*_0_ and *p*_1_ be the respective densities. By assumption *p*_0_(***x***) > 0 a.s. and $h_{j}^{1∕2}(x)>0$ a.s. for all *j* = 1,…,*m*. Therefore, we must have ∇ log *p*_1_(***x***) = ∇ log *p*_0_(***x***) a.s., or equivalently, *p*_1_(***x***) = const × *p*_0_(***x***) almost surely in $R_{+}^{m}$. Since *p*_1_ and *p*_0_ are continuous densities supported on $R_{+}^{m}$, it follows that *p*_1_(***x***) = *p*_0_(***x***) for all $x\in R_{+}^{m}$. ■

Choosing all *h_j_*(*x*) = *x*^2^ recovers the loss from (5). In our generalization, we will focus on using functions *h_j_* that are increasing but are bounded or grow rather slowly. This will alleviate the need to estimate higher moments, leading to better practical performance and improved theoretical guarantees.

We will consider the following assumptions:

(A1) p0(x)hj(xj)∂jlogp(x)∣xj↘0+xj↗+∞=0,∀x−j∈R+m−1,∀p∈P+;∣

(A2) $$E_{p_{0}}\|\nabla \;\log\;p(X)\circ h^{1∕2}(X){\|}_{2}^{2}<+\infty ,\;\;E_{p_{0}}\|(\nabla \;\log\;p(X)\circ h(X){)}^{\prime }{\|}_{1}<+\infty ,\;\;\forall p\in P_{+},$$

where ∂jlogp(x)∣≡∂logp(y)∂yj∣y=x, f(x)∣xj↘0+xj↗+∞≡limxj↗+∞f(x)−limxj↘0f(x)∣, “$\forall p\in P_{+}$” is a shorthand for “for all *p* being the density of some $P\in P_{+}$”, and the prime symbol denotes component-wise differentiation. While the second half of (A2) was not made explicit in Hyvärinen (2005, 2007), (A1)-(A2) were both required for integration by parts and Fubini-Tonelli to apply.

Once the forms of *p*_0_ and *p* are given, sufficient conditions for ***h*** for Assumptions (A1)-(A2) to hold are easy to find. In particular, (A1) and (A2) are easily satisfied and verified for exponential families.

Integration by parts yields the following theorem which shows that *J_**h**_* from (6) is an expectation (under *P*_0_) of a function that does not depend on *p*_0_, similar to (4). The proof is given in Appendix A.1.

**Theorem 3** *Under (A1) and (A2), the loss from (6) equals*

(7) Jh(P)=∫R+mp0(x)∑j=1m[hj′(xj)∂j(logp(x))+hj(xj)∂jj(logp(x))]+[12hj(xj)(∂j(logp(x)))2]dx

*plus a constant independent of p*.

Given a data matrix $x\in R^{n\times m}$ with rows ***X***^(*i*)^, we define the sample version of (7) as

(8) J^h(P)=1n∑i=1n∑j=1m{hj′(Xj(i))∂j(logp(X(i)))}+hj(xj(i)){[∂jj(logp(X(i)))+12(∂j(logp(X(i))))2]}.

Subsequently, for a distribution *P* with density *p*, we let *J_**h**_*(*p*) ≡ *J_**h**_*(*P*). Similarly, when a distribution *P_**θ**_* with density *p_**θ**_* is associated to a parameter vector ***θ***, we write *J_**h**_*(*θ*) ≡ *J_**h**_*(*p_**θ**_*) ≡ *J_**h**_*(*P_θ_*). We apply similar conventions to the sample version ${\hat{J}}_{h}(P)$. We note that this type of loss is also treated in slightly different settings in Parry (2016) and Almeida and Gidas (1993).

**Remark 4** In the one-dimensional case, using the notation in Parry et al. (2012), *J_**h**_*(*P*) and ${\hat{J}}_{h}(P)$ correspond to *d*(*P*_0_, *P*) and *S*(*x*, *P*), respectively, and can be generated by $\phi (x,p,p_{1})\equiv -h(x)p_{1}^{2}∕(2p)$ (c.f. Equations (39), (51), (53) and Section 10.1 therein). Thus Theorem 3 follows from this correspondence. While (A1) is equivalent to the condition implied by the boundary divergence *d_b_* = 0 in that paper, (A2), which we assume for invoking Fubini-Tonelli due to multi-dimensionality, is not present. On the other hand, while Parry (2016) treats the multivariate case, it does not cover the connection between our *J_**h**_* and ${\hat{J}}_{h}$. Since *ϕ* is concave but not strictly concave in (*p*, *p*_1_), the results in Parry (2016) only imply that *P*_0_ is *a* minimizer, a weaker conclusion than Proposition 2.

## 3. Exponential Families

In this section, we study the case where $P_{+}\equiv \{p_{\theta }\;:\theta \in \Theta \}$ is an exponential family comprising continuous distributions with support $R_{+}^{m}$. More specifically, we consider densities that are indexed by the canonical parameter $\theta \in R^{r}$ and have the form

(9) $$\log\;p_{\theta }(x)=\theta^{⊤}t(x)-\psi (\theta )+b(x),\;\;\;x\in R_{+}^{m},$$

where $t(x)\in R_{+}^{r}$ comprises the sufficient statistics, *ψ*(***θ***) is a normalizing constant depending on ***θ*** only, and *b*(***x***) is the base measure, with ***t*** and *b* a.s. differentiable with respect to each component. Define $t_{j}^{\prime }(x)\equiv (\partial_{j}t_{1}(x),\ldots ,\partial_{j}t_{r}(x){)}^{⊤}$ and $b_{j}^{\prime }(x)\equiv \partial_{j}b(x)$.

**Theorem 5** *Under Assumptions (A1)-(A2) from Section 2.2, the empirical generalized **h**-score matching loss (8) can be rewritten as a quadratic function in* $\theta \in R^{r}$:

(10) $${\hat{J}}_{h}(p_{\theta })=\frac{1}{2}\theta^{⊤}\Gamma (x)\theta -g(x{)}^{⊤}\theta +\text{const},\;\;\;where$$

(11) $$\Gamma (x)=\frac{1}{n}\sum_{i=1}^{n}\sum_{j=1}^{m}h_{j}(X_{j}^{\left(i\right)})t_{j}^{\prime }(X^{\left(i\right)})t_{j}^{\prime }(X^{\left(i\right)}{)}^{⊤}\;\;\;and$$

(12) $$g(x)=-\frac{1}{n}\sum_{i=1}^{n}\sum_{j=1}^{m}\left[h_{j}(X_{j}^{\left(i\right)})b_{j}^{\prime }(X^{\left(i\right)})t_{j}^{\prime }(X^{\left(i\right)})+h_{j}(X_{j}^{\left(i\right)})t_{j}^{\prime \prime }(X^{\left(i\right)})+h_{j}^{\prime }(X_{j}^{\left(i\right)})t_{j}^{\prime }(X_{i})\right]$$

*are sample averages of functions of the data matrix* **x** *only*.

Define $\Gamma_{0}\equiv E_{p_{0}}\Gamma (x)$, $g_{0}\equiv E_{p_{0}}g(x)$, and $\Sigma_{0}\equiv E_{p_{0}}[(\Gamma (x)\theta_{0}-g(x))(\Gamma (x)\theta_{0}-g(x){)}^{⊤}]$.

**Theorem 6** *Suppose that*

(C1) Γisa.s.invertible,and

(C2) $$\Gamma_{0},\Gamma_{0}^{-1},\;g_{0}\;and\;\Sigma_{0}\;exist\;and\;are\;entry\text{-}wise\;finite.$$

*Then the minimizer of (10) is a.s. unique with closed-form solution* $\hat{\theta }\equiv \Gamma (x{)}^{-1}g(x)$. *Moreover*,

$$\hat{\theta }\to_{a.s.}\theta_{0}\;\;and\;\;\sqrt{n}(\hat{\theta }-\theta_{0})\to_{d}\;N_{r}\;(0,\Gamma_{0}^{-1}\Sigma_{0}\Gamma_{0}^{-1})\;\;as\;n\to \infty .$$

Theorems 5 and 6 are proved in Appendix A.2. Theorem 5 clarifies the quadratic nature of the loss, and Theorem 6 provides a basis for asymptotically valid tests and confidence intervals for the parameter ***θ***. Note that Condition (C1) holds if and only if *h_j_*(*X_j_*) > 0 a.s. and $[t_{j}^{\prime }(X^{\left(1\right)}),\ldots ,t_{j}^{\prime }(X^{\left(n\right)})]\in R^{r\times n}$ has rank *r* a.s. for some *j* = 1,…, *m*.

The conclusion in Theorem 6 indicates that, similar to the estimator in Hyvärinen (2007) with *h_j_*(*x*) = *x*^2^, the closed-form solution for our generalized $\hat{\theta }$ allows one to consistently estimate the canonical parameter in an exponential family distribution without needing to calculate the often complicated normalizing constant *ψ*(***θ***) or resort to numerical methods. Computational details are explicated in Section 5.3.

Below we illustrate the estimator $\hat{\theta }$ in the case of univariate truncated normal distributions. We assume (A1)-(A2) and (C1)-(C2) throughout.

**Example 3.1** *Univariate* *(m* = *r* = 1*) truncated normal distributions for mean parameter μ and variance parameter σ*^2^ *have density*

(13) $$p_{\mu ,\sigma^{2}}(x)\propto \text{exp}\left\{-\frac{(x-\mu {)}^{2}}{2\sigma^{2}}\right\},\;\;x\in R_{+}.$$

*If σ*^2^ *is known but μ unknown, then writing the density in canonical form as in (9) yields*

$$p_{\theta }(x)\propto \text{exp}\;\{\theta t(x)+b(x)\},\;\;\theta \equiv \frac{\mu }{\sigma^{2}},\;\;t(x)\equiv x,\;\;b(x)=-\frac{x^{2}}{2\sigma^{2}}.$$

*Given an i.i.d. sample X*_1_,… ,*X_n_* ~ *p*_*μ*_0_,*σ*^2^_*, the generalized h-score matching estimator of μ is*

$${\hat{\mu }}_{h}\equiv \frac{\sum_{i=1}^{n}h(X_{i})X_{i}-\sigma^{2}h^{\prime }(X_{i})}{\sum_{i=1}^{n}h(X_{i})}.$$

*If* lim_*x*↘0+_ *h*(*x*) = 0, lim_*x*↗+∞_ *h*^2^(*x*)(*x* – *μ*_0_)*p*_*μ*_0_,*σ*^2^_(*x*) = 0 *and the expectations are finite* (*for example, when h*(*x*) = *o*(exp(*Mx*^2^)) *for* $M<\frac{1}{4\sigma^{2}}$*), then*

$$\sqrt{n}({\hat{\mu }}_{h}-\mu_{0})\to_{d}N\left(0,\frac{E_{0}[\sigma^{2}h^{2}(X)+\sigma^{4}h^{\prime 2}(X)]}{E_{0}^{2}[h(X)]}\right).$$

*We recall that the* Cramér-Rao lower bound *(i.e. the lower bound on the variance of any unbiased estimator) for estimating μ is*

$$\frac{\sigma^{4}}{\text{var}(X-\mu_{0})}.$$

**Example 3.2** *Consider the univariate truncated normal distributions from (13) in the setting where the mean parameter μ is known but the variance parameter σ*^2^ > *0 is unknown. In canonical form as in (9), we write*

$$p_{\theta }(x)\propto \text{exp}\;\{\theta t(x)+b(x)\},\;\;\theta \equiv \frac{1}{\sigma^{2}},\;\;t(x)\equiv -(x-\mu {)}^{2}∕2,\;\;b(x)=0.$$

*Given an i.i.d. sample* $X_{1},\ldots ,X_{n}∼p_{\mu ,\sigma_{0}^{2}}$*, the generalized h-score matching estimator of σ*^2^ *is*

$${\hat{\sigma }}_{h}^{2}\equiv \frac{\sum_{i=1}^{n}h(X_{i})(X_{i}-\mu {)}^{2}}{\sum_{i=1}^{n}h(X_{i})+h^{\prime }(X_{i})(X_{i}-\mu )}.$$

*If, in addition to the assumptions in Example 3.1,* $\underset{x↗+\infty }{\text{lim}}h^{2}(x)(x-\mu {)}^{3}p_{\mu ,\sigma_{0}^{2}}(x)=0$, *then*

$$\sqrt{n}({\hat{\sigma }}_{h}^{2}-\sigma_{0}^{2})\to_{d}N\left(0,\frac{2\sigma_{0}^{6}E_{0}[h^{2}(X)(X-\mu {)}^{2}]+\sigma_{0}^{8}E_{0}[h^{\prime 2}(X)(X-\mu {)}^{2}]}{E_{0}^{2}[h(X)(X-\mu {)}^{2}]}\right).$$

*Moreover, the* Cramér-Rao lower bound *for estimating σ*^2^ *is*

$$\frac{4\sigma_{0}^{8}}{\text{var}(X-\mu {)}^{2}}.$$

**Remark 7** In Example 3.2, if *μ*_0_ = 0, then *h*(*x*) ≡ 1 also satisfies (A1)-(A2) and (C1)-(C2) and one recovers the sample variance $\frac{1}{n}\sum_{i}X_{i}^{2}$, which obtains the Cramér-Rao lower bound.

In these examples, there is a benefit in using a bounded function *h*, which can be explained as follows. When *μ* ≫ *σ*, there is effectively no truncation to the Gaussian distribution, and our method adapts to using low moments in (6), since a bounded and increasing *h*(*x*) becomes almost constant as it reaches its asymptote for *x* large. Hence, we effectively revert to the original score matching (recall Section 2.1). In the other cases, the truncation effect is significant and our estimator uses higher moments accordingly.

**Figure 1:**
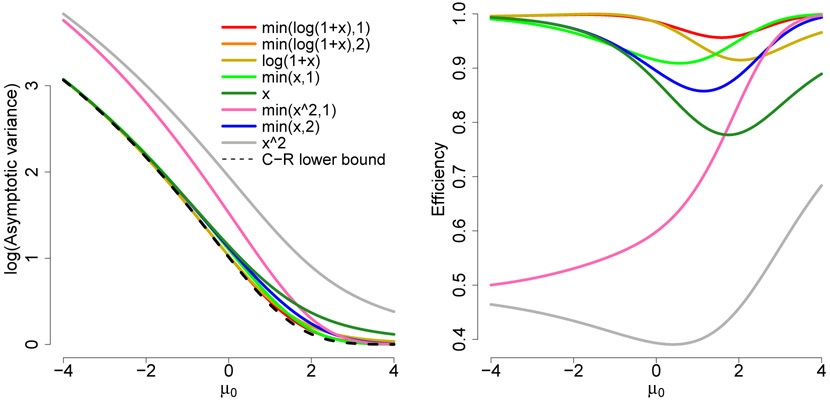
Log of asymptotic variance and efficiency with respect to the Cramér-Rao bound for ${\hat{\mu }}_{h}(\sigma^{2}=1\;\text{known})$.

Figure 1 plots the asymptotic variance of ${\hat{\mu }}_{h}$ from Example 3.1, with *σ* = 1 known. Efficiency as measured by the Cramér-Rao lower bound divided by the asymptotic variance is also shown. We see that two truncated versions of log(1 + *x*) have asymptotic variance close to the Cramér-Rao bound. This asymptotic variance is also reflective of the variance for smaller finite samples.

**Figure 2:**
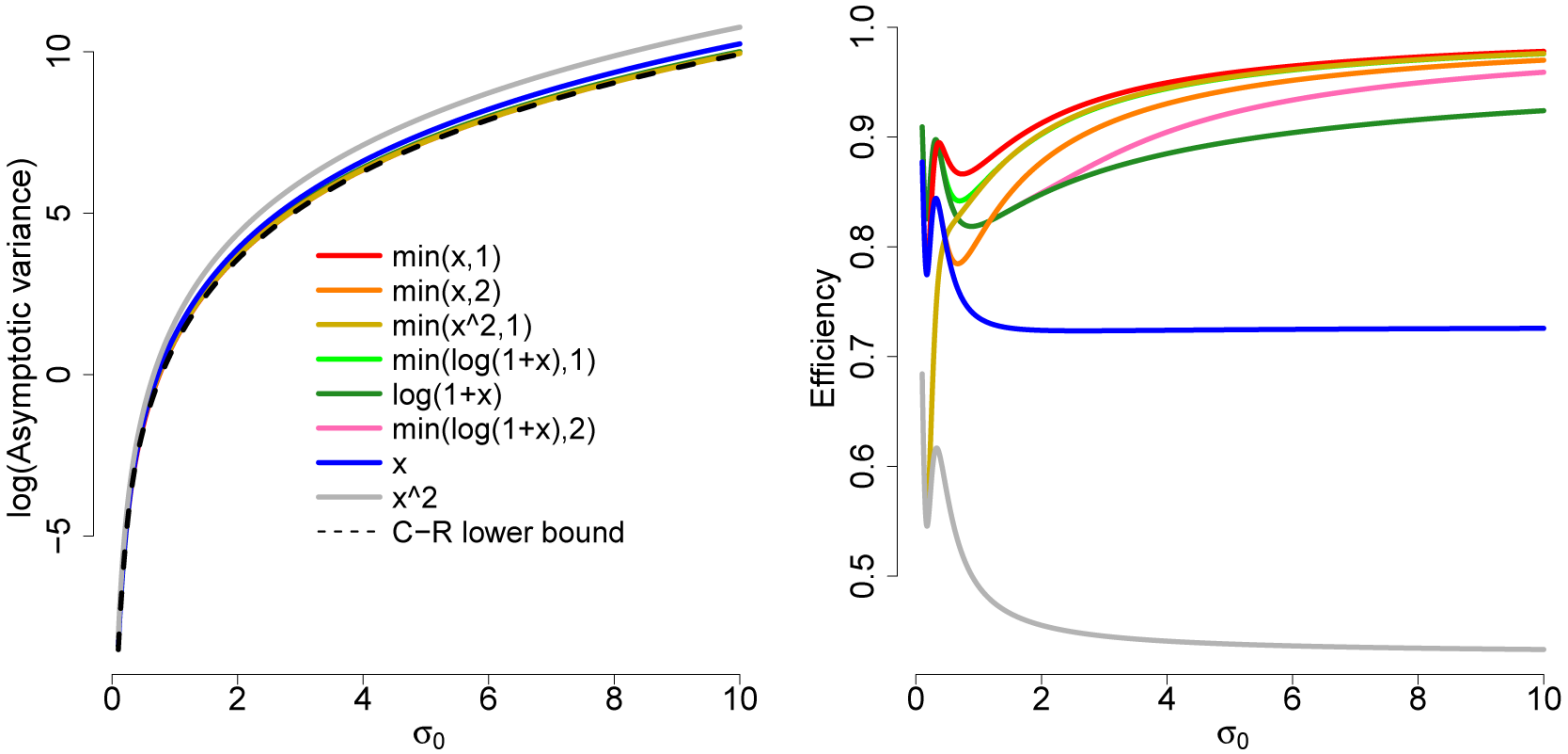
Log of asymptotic variance and efficiency with respect to the Cramér-Rao bound for ${\hat{\sigma }}_{h}^{2}(\mu =0.5\;\text{known})$.

Figure 2 is the analog of Figure 1 for ${\hat{\sigma }}_{h}^{2}$ from Example 3.2 with *μ* = 0.5 known. While the specifics are a bit different the benefits of using bounded or slowly growing *h* are again clear. We note that when *σ* is small, the effect of truncation to the positive part of the real line is small.

In both plots we order/color the curves based on their overall efficiency, so they have different colors in one from the other, although the same functions are presented. For all functions presented here (A1)-(A2) and (C1)-(C2) are satisfied.

## 4. Regularized Generalized Score Matching

In high-dimensional settings, when the number *r* of parameters to estimate may be larger than the sample size *n*, it is hard, if not impossible, to estimate the parameters consistently without turning to some form of regularization. More specifically, for exponential families, condition (C1) in Section 3 fails when *r* > *n*. A popular approach is then the use of *ℓ*_1_ regularization to exploit possible sparsity.

Let the data matrix $x\in R^{n\times m}$ comprise *n* i.i.d. samples from distribution *P*_0_. Assume *P*_0_ has density *p*_0_ belonging to an exponential family $P_{+}\equiv \{p_{\theta }\;:\theta \in \Theta \}$, where $\Theta \subseteq R^{r}$. Adding an *ℓ*_1_ penalty to (10), we obtain the regularized generalized score matching loss

(14) $$\frac{1}{2}\theta^{⊤}\Gamma (x)\theta -g(x{)}^{⊤}\theta +\lambda \|\theta {\|}_{1}$$

as in Lin et al. (2016). The loss in (14) involves a quadratic smooth part as in the familiar lasso loss for linear regression. However, although the matrix **Γ** is positive semidefinite, the regularized loss in (14) is not guaranteed to be bounded unless the tuning parameter *λ* is sufficiently large—a problem that does not occur in lasso. We note that here, and throughout, we suppress the dependence on the data **x** for **Γ**(**x**), ***g***(**x**) and derived quantities.

For a more detailed explanation, note that that by (11), **Γ** = **H**^⊤^**H** for some $H\in R^{nm\times r}$. In the high-dimensional case, the rank of **Γ**, or equivalently **H**, is at most *nm* < *r*. Hence, **Γ** is not invertible and ***g*** does not necessarily lie in the column span of **Γ**. Let Ker(**Γ**) be the kernel of **Γ**. Then there may exist ***ν*** ∈ Ker(**Γ**) with ***g***^⊤^***ν*** ≠ 0. In this case, if

$$0\leq \lambda <\underset{\nu \in \text{Ker}(\Gamma )}{\text{sup}}|g^{⊤}\nu |∕\|\nu {\|}_{1},$$

there exists ***ν*** ∈ Ker(**⊤**) with $\frac{1}{2}\nu^{⊤}\Gamma \nu =0$ and −***g***^⊤^***ν*** + *λ*∥***ν***∥_1_ < 0. Evaluating at ***θ***(*a*) = *a*·***ν*** for scalar *a* > 0, the loss becomes *a*(−***g***^⊤^***ν*** + *λ*∥***ν***∥_1_), which is negative and linear in *a*, and thus unbounded below. In this case no minimizer of (14) exists for small values of *λ*. This issue also exists for the estimators from Zhang and Zou (2014) and Liu and Luo (2015), which correspond to score matching for GGMs. We note that in the context of estimating the interaction matrix in pairwise models, *r* = *m*^2^; thus, the condition *nm* < *r* reduces to *n* < *m*, or *n* < *m* + 1 when both **K** and ***η*** are estimated.

To circumvent the unboundedness problem, we add small values *γ*_*ℓ*_ > 0 to the diagonal entries of **Γ**, which become **Γ**_*ℓ*,*ℓ*_ + *γ*_*ℓ*_, *ℓ* = 1,…, *r*. This is in the spirit of work such as Ledoit and Wolf (2004) and corresponds to an elastic net-type penalty (Zou and Hastie, 2005) with weighted *ℓ*_2_ penalty $\sum_{\ell =1}^{r}\gamma_{\ell }\theta_{\ell }^{2}$. After this modification, **Γ** is positive definite, our regularized loss is strongly convex in ***θ***, and a unique minimizer exists for all *λ* ≥ 0. For the special case of truncated GGMs, we will show that a result on consistent estimation holds if we choose *γ_ℓ_* = *δ*_0_**Γ**_*ℓ*,*ℓ*_ for a suitably small constant *δ*_0_ > 0, for which we propose a particular choice to avoid tuning. This choice of *γ*_*ℓ*_ depends on the data through **Γ**_*ℓ*,*ℓ*_.

**Definition 8** *For* $\gamma \in R_{+}^{r}\setminus \{0\}$, *let* **Γ_*γ*_** ≡ **Γ** + diag(*γ*). *The* regularized generalized ***h***-score matching estimator *with tuning parameter λ* ≥ 0 *and* amplifier ***γ*** *is the estimator*

(15) $$\hat{\theta }\in \underset{\theta \in \Theta }{\text{argmin}}{\hat{J}}_{h,\lambda ,\gamma }(\theta )\equiv \underset{\theta \in \Theta }{\text{argmin}}\frac{1}{2}\;\theta^{⊤}\Gamma_{\gamma }(x)\theta -g(x{)}^{⊤}\theta +\lambda \|\theta {\|}_{1}.$$

In the case where ***γ*** = (*δ* – 1)diag(**Γ**) for some *δ* > 1, we also call *δ* the *multiplier*. We note that $\hat{\theta }$ from (15) is a *piecewise linear* function of *λ* (Lin et al., 2016).

## 5. Score Matching for Graphical Models for Non-negative Data

In this section we apply our generalized score matching estimator to a general class of graphical models for non-negative data.

### 5.1. A General Framework of Pairwise Interaction Models

We consider the class of pairwise interaction power models with density introduced in (1). We recall the form of the density:

(16) $$p_{\eta ,K}(x)\propto \text{exp}\left(-\frac{1}{2a}x^{a⊤}Kx^{a}+\eta^{⊤}\frac{x^{b}-1_{m}}{b}\right)1_{R_{+}^{m}}(x),$$

where *a* and *b* are known constants, and the interaction matrix **K** and the vector ***η*** are parameters. When *b* = 0, we use the convention that $\frac{x^{0}-1}{0}$ ≡ log *x* and apply the logarithm element-wise. Our focus will be on the interaction matrix **K** that determines the conditional independence graph through its support *S*(**K**) ≡ {(*i*, *j*) : *κ_ij_* ≠ 0}. However, unless ***η*** is known or assumed to be zero, we also need to estimate ***η*** as a nuisance parameter. In the case where we assume ***η*** ≡ **0** is known (i.e. the linear part (***x***^*b*^ – **1**_*m*_)/*b* is not present), we call the distribution (and the corresponding estimator) a *centered* distribution (estimator), in contrast to the general case termed *non-centered* when we assume ***η*** ≠ **0** or unknown.

We first give a set of sufficient conditions for the density to be valid, i.e., the right-hand side of (16) to be integrable. The proof is given in Appendix A.3.

**Theorem 9** *Define conditions*

(CC1) $$K\;is\;\text{strictly co-positive},\;i.e.,\;v^{⊤}Kv>0\;for\;all\;v\in R_{+}^{m}\setminus \{0\};$$

(CC2) 2a>b>0;

(CC3) $$a>0,\;b=0,\;and\;\eta_{j}>-1\;for\;j=1,\ldots ,m\;(\eta ≻-1_{m}).$$

*In the non-centered case, if (CC1) and one of (CC2) and (CC3) holds, then the function on the right-hand side of (16) is integrable over* $R_{+}^{m}$. *In the centered case, (CC1) and a* > 0 *are sufficient*.

We emphasize that (CC1) is a weaker condition than positive definiteness. Criteria for strict co-positivity are discussed in Väliaho (1986).

### 5.2. Implementation for Different Models

In this section we give some implementation details for the regularized generalized ***h***-score matching estimator defined in (15) applied to the pairwise interaction models from (16). We again let $\Psi \equiv (K^{⊤},\eta {)}^{⊤}\in R^{(m+1)\times m}$. The unregularized loss is then

$${\hat{J}}_{h}(P)=\frac{1}{2}\text{vec}(\Psi {)}^{⊤}\Gamma (x)\text{vec}(\Psi )-g(x{)}^{⊤}\text{vec}(\Psi ).$$

The general form of the matrix **Γ** and the vector ***g*** in the loss were given in equations (10)-(12). Here $\Gamma \in R^{(m+1)m\times (m+1)m}$ is block-diagonal, with the *j*-th $R^{\left(m+1)\times (m+1\right)}$ block

(17) $$\Gamma_{j}(x)\equiv \left[\begin{array}{cc} \Gamma_{11,j} & \Gamma_{12,j}\\ \Gamma_{12,j}^{⊤} & \Gamma_{22,j} \end{array}\right]\;\;\equiv \frac{1}{n}\sum_{i=1}^{n}\left[\begin{array}{cc} h_{j}\left(X_{j}^{\left(i\right)}\right)X_{j}^{(i{)}^{2a-2}}X^{(i{)}^{a}}X^{(i{)}^{a⊤}} & -h_{j}\left(X_{j}^{\left(i\right)}\right)X_{j}^{(i{)}^{a+b-2}}X^{(i{)}^{a}}\\ -h_{j}\left(X_{j}^{\left(i\right)}\right)X_{j}^{(i{)}^{a+b-2}}X^{(i{)}^{a⊤}} & h_{j}\left(X_{j}^{\left(i\right)}\right)X_{j}^{(i{)}^{2b-2}} \end{array}\right]$$

(18) $$=\frac{1}{n}y^{⊤}y,\;\;y\equiv \left[-(\sqrt{h_{j}(X_{j})}\circ X_{j}^{a-1})\circ x^{a}\;\;\sqrt{h_{j}(X_{j})}\circ X_{j}^{b-1}\right]\in R^{n,m+1},$$

where the ○ product between a vector and a matrix means an elementwise multiplication of the vector with each *column* of the matrix, and $h_{j}(X_{j})\equiv [h_{j}(X_{j}^{\left(1\right)}),\ldots ,h_{j}(X_{j}^{\left(n\right)}{)}^{⊤}]\in R^{m}$.

Furthermore, $g\equiv \left[\begin{array}{c} \text{vec}(g_{1})\\ g_{2} \end{array}\right]\in R^{(m+1)m}$, where **g**_1_ and ***g***_2_ correspond to each entry of **K** and ***η***, respectively. The *j*-th column of $g_{1}\in R^{m\times m}$, written as ***g***_1,*j*_(**x**), is

$$\frac{1}{n}\sum_{i=1}^{n}\left(h_{j}^{\prime }\left(X_{j}^{\left(i\right)}\right)X_{j}^{(i{)}^{a-1}}+(a-1)h_{j}\left(X_{j}^{\left(i\right)}\right)X_{j}^{(i{)}^{a-2}}\right)X^{(i{)}^{a}}+ah_{j}\left(X_{j}^{\left(i\right)}\right)X_{j}^{(i{)}^{2a-2}}e_{j,m},$$

where ***e***_*j*,*m*_ is the *m*-vector with 1 at the *j*-th position and 0 elsewhere, and the *j*-th entry of $g_{2}\in R^{m}$ is

$$g_{2,j}=\frac{1}{n}\sum_{i=1}^{n}-h_{j}^{\prime }\left(X_{j}^{\left(i\right)}\right)X_{j}^{(i{)}^{b-1}}-(b-1)h_{j}\left(X_{j}^{\left(i\right)}\right)X_{j}^{(i{)}^{b-2}}.$$

These formulae also hold for *b* = 0 since **Γ** and ***g*** only depend on the gradient of the log density, and $\frac{d(x^{b}-1)∕b}{dx}=x^{b-1}$ also holds for *b* = 0. In the centered case where we know ***η***_0_ ≡ **0**, we only estimate $K\in R^{m\times m}$, and $\Gamma \in R^{m^{2}\times m^{2}}$ is still block-diagonal, with the *j*-th block being the **Γ**_11,*j*_ submatrix in (17), while ***g*** is just vec(**g**_1_). Since *b* only appears in the ***η*** part of the density, the formulae only depend on *a* in the centered case.

We emphasize that it is indeed necessary to introduce amplifiers ***γ*** ≻ **0** or a multiplier *δ* > 1 in addition to the *ℓ*_1_ penalty. It is clear from (18) that rank(**Γ**_*j*_) ≤ min{*n*, *m* + 1} (or min{*n*, *m*} if centered). Thus, **Γ** is non-invertible when *n* ≤ *m* (or *n* < *m* if centered) and ***g*** need not lie in its column span.

We claim that including amplifiers/multipliers for the submatrices **Γ**_11,*j*_ only is sufficient for unique existence of a solution for all penalty parameters *λ* ≥ 0. To see this, consider any nonzero vector $\nu \in R^{m+1}$. Partition it as ***ν*** ≡ (***ν***_1_, ***ν***_2_) with $\nu_{1}\in R^{m}$. Let **Γ**_*j*,***γ***_ be our amplified version of the matrix **Γ**_*j*_ from (21), so

$$\Gamma_{j,\gamma }=\left(\begin{array}{cc} \Gamma_{11,j}+\text{diag}(\gamma_{1},\ldots ,\gamma_{m}) & \Gamma_{12,j}\\ \Gamma_{12,j}^{⊤} & \Gamma_{22,j} \end{array}\right).$$

As **Γ**_*j*_ itself is positive semidefinite, we find that if at least one of the first *m* entries of ***ν*** is nonzero then

$$\nu^{⊤}\Gamma_{j,\gamma }\nu \;\geq \;\nu^{⊤}\Gamma_{j}\nu +\sum_{k=1}^{m}\nu_{k}^{2}\gamma_{k}\;\geq \;\sum_{k=1}^{m}\nu_{k}^{2}\gamma_{k}>0.$$

If only the last entry of ***ν*** is nonzero then

$$\nu^{⊤}\Gamma_{j,\gamma }\nu \;=\;\nu_{m+1}^{2}\Gamma_{22,j}\;>\;0$$

almost surely; recall that $\Gamma_{22,j}=\frac{1}{n}\sum_{i=1}^{n}h_{j}\left(X_{j}^{\left(i\right)}\right)X_{j}^{2b-2}$. We conclude that **Γ**_*j*,***γ***_ (and thus the entire amplified **Γ**) is a.s. positive definite, which ensures unique existence of the loss minimizer.

Given the formulae for **Γ** and ***g***, one adds the *ℓ*_1_ penalty on **Ψ** to get the regularized loss (24). Our methodology readily accommodates two different choices of the penalty parameter *λ* for **K** and ***η***. This is also theoretically supported for truncated GGMs, since if the ratio of the respective values *λ*_**K**_ and *λ*_***η***_ is fixed, the proof of the theorems in Section 6 can be easily modified by replacing ***η*** by (*λ*_***η***_/*λ*_**K**_)***η***. To avoid picking two tuning parameters, one may also choose to remove the penalty on ***η*** altogether by profiling out ***η*** and solve for $\hat{\eta }\equiv \Gamma_{22}^{-1}\left(g_{2}-\Gamma_{12}^{⊤}\text{vec}(\hat{K})\right)$, with $\hat{K}$ the minimizer of the profiled loss

(19) $${\hat{J}}_{h,\lambda ,\gamma ,\text{profile}}(K)\equiv \frac{1}{2}\text{vec}(K{)}^{⊤}\Gamma_{\gamma ,11.2}\text{vec}(K)-(g_{1}-\Gamma_{12}\Gamma_{22}^{-1}g_{2}{)}^{⊤}\text{vec}(K)+\lambda \|K{\|}_{1},$$

where the Schur complement $\Gamma_{\gamma ,11.2}\equiv \Gamma_{\gamma ,11}-\Gamma_{12}\Gamma_{22}^{-1}\Gamma_{12}^{⊤}$ is a.s. positive definite such that the profiled estimator exists a.s. for all *λ* ≥ 0. This profiled approach corresponds to choosing *λ*_***η***_/*λ*_**K**_ = 0. A detailed theoretical analysis of the profiled estimator is beyond the scope of this paper, however. We note that in the other extreme, with *λ*_***η***_/*λ*_**K**_ = +∞, the non-centered estimator reduces to the estimator from the centered case.

**Example 5.3** *The truncated normal model comprises the density*

(20) $$p_{\mu ,K}(x)\propto \text{exp}\left\{-\frac{1}{2}(x-\mu {)}^{⊤}K(x-\mu )\right\}1_{[0,\infty {)}^{m}}(x).$$

*This corresponds to (16) with a* = *b* = 1*, and* ***η*** = K***μ***. *The j-th* (*m* + 1) × (*m* + 1) *block of* **Γ**(**x**) *is*

(21) $$\frac{1}{n}\left[\begin{array}{cc} x^{⊤}\text{diag}(h_{j}(X_{j}))x & -x^{⊤}h_{j}(X_{j})\\ -h_{j}(X_{j}{)}^{⊤}x & h_{j}(X_{j}{)}^{⊤}\;1_{n} \end{array}\right].$$

*Partitioning the vector **g***(**x**) *into m subvectors* $g_{j}(x)\in R^{m+1}$*, where the entries of **g**_j_*(**x**) *correspond to column* **Ψ***_j_, the k-th entry of **g**_j_*(**x**) *is*

(22) gjk(x)≡{1n∑i=1nhj′(Xj(i))Xk(i)ifk≤m,k≠j,1n∑i=1nhj′(Xj(i))Xk(i)+hj(Xj(i))ifk=j,−1n∑i=1nhj′(Xj(i))ifk=m+1.}

**Example 5.4** *The exponential square-root graphical model in Inouye et al. (2016) has*

$$p_{\eta ,K}(x)\propto \text{exp}\left(-{\sqrt{x}}^{⊤}K\sqrt{x}+2\eta^{⊤}\sqrt{x}\right)1_{[0,\infty {)}^{m}}(x),$$

*which corresponds to (16) with a* = *b* = 1/2. *We refer to this as the* exponential *model. In this case, the j-th* $R^{\left(m+1)\times (m+1\right)}$ *block of* **Γ** *is*

$$\Gamma_{j}(x)\equiv \frac{1}{n}\sum_{i=1}^{n}\frac{h_{j}\left(X_{j}^{\left(i\right)}\right)}{X_{j}^{\left(i\right)}}\left(\begin{array}{c} -\sqrt{X^{\left(i\right)}}\\ 1 \end{array}\right)\left(-{\sqrt{X^{\left(i\right)}}}^{⊤},1\right)$$

*and **g*** = vec(**g**_0_)*, where the j-th column of* $g_{0}\in R^{(m+1)\times m}$ *is*

$$g_{j}(x)\equiv \frac{1}{n}\sum_{i=1}^{n}\frac{2h_{j}^{\prime }\left(X_{j}^{\left(i\right)}\right)X_{j}^{\left(i\right)}-h_{j}\left(X_{j}^{\left(i\right)}\right)}{2X_{j}^{(i{)}^{3∕2}}}\left(\begin{array}{c} \sqrt{X^{\left(i\right)}}\\ -1 \end{array}\right)+\frac{h_{j}\left(X_{j}^{\left(i\right)}\right)}{2X_{j}^{\left(i\right)}}e_{j,m+1}.$$

**Example 5.5** *If a* = 1/2 *and b* = 0, *then (16) becomes*

(23) $$p_{\eta ,K}(x)\propto \text{exp}\left(-{\sqrt{x}}^{⊤}K\sqrt{x}+\eta^{⊤}\;\log(x)\right)1_{(0,\infty {)}^{m}}(x).$$

*If* **K** *is diagonal in this case, then **X*** ~ *p*_***η***,**K**_ *has independent entries with X_j_ following the gamma, distribution with rate κ_jj_ and shape η_j_* + 1*, which gives an intuition for condition (CC3) η_j_* > −1 *in Theorem 9. We can thus view (23) as a multivariate gamma, distribution with pairwise interactions among the covariates, and call this the gamma model. For this model, the j-th block of* **Γ** *is*

$$\Gamma_{j}(x)\equiv \frac{1}{n}\sum_{i=1}^{n}\frac{h_{j}\left(X_{j}^{\left(i\right)}\right)}{X_{j}^{(i{)}^{2}}}\left(\begin{array}{c} -\sqrt{X_{j}^{\left(i\right)}X^{\left(i\right)}}\\ 1 \end{array}\right)\left(-{\sqrt{X_{j}^{\left(i\right)}X^{\left(i\right)}}}^{⊤},1\right)$$

*and the part of **g** corresponding to* **K**_*j*_ *is*

$$g_{1,j}(x)\equiv \frac{1}{n}\sum_{i=1}^{n}\frac{2h_{j}^{\prime }\left(X_{j}^{\left(i\right)}\right)X_{j}^{\left(i\right)}-h_{j}\left(X_{j}^{\left(i\right)}\right)}{2X_{j}^{(i{)}^{3∕2}}}\sqrt{X^{\left(i\right)}}+\frac{h_{j}\left(X_{j}^{\left(i\right)}\right)}{2X_{j}^{\left(i\right)}}e_{j,m},$$

*while the part for η_j_ is*

$$g_{2.j}(x)=\frac{1}{n}\sum_{i=1}^{n}\frac{h_{j}\left(X_{j}^{\left(i\right)}\right)}{X_{j}^{(i{)}^{2}}}-\frac{h_{j}^{\prime }\left(X_{j}^{\left(i\right)}\right)}{X_{j}^{\left(i\right)}}.$$

We note that the **Γ**_11,*j*_ sub-matrix of **Γ**_*j*_ and the ***g***_1,*j*_ sub-vector of ***g**_j_* for the gamma model are the same as those for the exponential model, since *a* = 1/2 in both cases and the parts involving **K** in the densities are the same.

### 5.3. Computational Details

In the most general exponential family setting, as in Eq. (10)-(12) in Theorem 5, the time complexity for forming $\Gamma \in R^{r\times r}$ and $g\in R^{r}$ is $O\left(nm(f_{b^{\prime }}(m)+r^{2}+r(f_{t^{\prime }}(m)+f_{t^{\prime \prime }}(m)))\right)$. Here *f_b′_*(*m*) is the average time complexity for calculating *∂_j_b*(***x***) over *j* = 1,…, *m*, and similarly *f*_*t*′_(*m*) for *∂_j_t_ℓ_*(***x***) and *f*_*t*″_(*m*) for *∂_jj_t_ℓ_*(***x***) over *j* = 1,…, *m* and *ℓ* = 1…, *r*. In many applications, however, these three functions would be constant in *m*, thus giving an $O(nmr^{2})$ computational complexity, with the dominating term coming from the operations for $t_{j}^{\prime }{t_{j}^{\prime }}^{⊤}$ in **Γ** since **Γ** is of dimension *r* × *r*.

For pairwise interaction power models, *r* = *m*^2^ and the formula above becomes $O(nm^{5})$. However, since **Γ** is block-diagonal with only *m*^3^ nonzero entries and by the special form of ***t***(***x***) = ***x**^a^**x***^*a*⊤^, the true complexity is in fact $O(nm^{3})$.

While the introduction of the *ℓ*_1_ penalty inevitably precludes the estimator from having a closed-form solution and introduces non-differentiability, state-of-art numerical optimization algorithms, such as coordinate-descent (Friedman et al., 2007), can be applied for fast estimation. To speed up estimation, one can usually use warm starts using the solution from the previous *λ*’s, as well as lasso-type strong screening rules (Tibshirani et al., 2012) to eliminate components of $\hat{\theta }$ that are known a priori to have zero estimates.

In our implementation for pairwise interaction models of Section 5.1 (that will become available in an R package), we optimize our loss functions with respect to a symmetric matrix $\hat{K}$; in the non-centered case the vector $\hat{\eta }$ is also included. We use a coordinate-descent method analogous to Algorithm 2 in Lin et al. (2016), where in each step we update each element of $\hat{K}$ and $\hat{\eta }$ based on the other entries from the previous steps, while maintaining symmetry. In our simulations in Section 7 we always scale the data matrix by column *ℓ*_2_ norms before proceeding to estimation. Note that estimation of $\hat{K}$ without symmetry can be parallelized as the loss can be decomposed into a sum over the columns.

### 5.4. Choice of the Function *h*

In this subsection we discuss the requirements on the function ***h*** as well as some reasonable choices of ***h***.

#### 5.4.1. Requirements on *h*

In Section 2.2, we presented two assumptions (A1) and (A2) under which the generalized score-matching loss is valid, i.e., the integration by parts is justified and Theorem 3 holds. In this section, we present some sufficient (and nearly necessary) requirements on ***h*** such that (A1) and (A2) are satisfied.

**Definition 10** *Suppose **h*** : $R_{+}^{m}\to R_{+}^{m}$ *with* ***h***(***x***) = (*h*_1_(*x*_1_),…, *h*_*m*_(*x*_*m*_))^⊤^*. We write that* $h\in H_{a,b}$ *(for simplicity we omit the dependency on m) if for all j* = 1,…, *m:*

- *h_j_ is absolutely continuous in every bounded sub-interval of* $R_{+}$*, and thus has derivative* $h_{j}^{\prime }$ *a.s.;*
- *h_j_*(*x*) > 0 *a.s. on* $R_{+}$;
- *h_j_ and* $h_{j}^{\prime }$ *are both bounded by some piecewise powers of x a.s. on* $R_{+}$;
- $\underset{x↘0+}{\text{lim}}h_{j}(x)∕x_{j}^{q}=0$, *where* q≡{max{1−a,1−b}ifb>0,1−η0,jifb=0.}.

**Theorem 11** *Assume every P in the family of distribution* $P_{+}$ *satisfies (CC1)-(CC3) and thus has finite normalizing constants. If* $h\in H_{a,b}$*, then (A1) and (A2) are satisfied*.

In centered models, where ***η*** ≡ **0**, we can assume *b* = 2*a* and iv) in the definition of $H_{a,2a}$ has *q* = 1 – *a*. For truncated GGMs, *a* = *b* = 1, so iv) in Definition 10 is simply ${\text{lim}}_{x_{j}↘0^{+}}\;h_{j}(x_{j})=0$.

In the case of *b* = 0, ***η*** is an unknown parameter, and (CC3) requires each of its component to be greater than −1. If one has prior information on ***η*** or restricts the parameter space for ***η***, the requirement reduces to $h_{j}(x_{j})=o(x_{j}^{1-\eta_{0,j}})$. as *x_j_* ↘ 0^+^. Otherwise, it suffices to require $h_{j}(x_{j})=o(x_{j}^{2})$. Note that this is only a condition for *x_j_* ↘ 0^+^, and the globally quadratic behavior of $h_{j}(x_{j})=x_{j}^{2}$ from the original score matching is not needed on the entire $R_{+}$, leaving opportunities for improvements.

#### 5.4.2. Reasonable Choices of *h*

Assume a common univariate *h* for all components in ***h***. Inspired by Theorem 11, we consider *h* that behaves like a power of *x* both as *x* ↗ +∞ and as *x* ↘ 0^+^. Since the requirements on the two tails are separate, we can choose *h* to be a piecewise defined function that joins two powers with possibly different degrees. In other words, *h*(*x*) = min(*x*^*p*_1_^, *cx*^*p*_2_^) for some powers *p*_1_ ≥ *p*_2_ ≥ 0 and constant *c* > 0. Only one constant *c* is required since generalized score matching is invariant to scaling of *h*. In determining the exact power of *p*_1_ we have the following considerations:

- In the centered case:
  (i) (A1) and (A2): Theorem 11 requires that *p*_1_ ≥ 1 – *a*.
  (ii) “Controlled **Γ** and ***g*** for ***x***^*a*^”: We propose avoiding poles at the origin for the entries of **Γ** and ***g***. The formula for **Γ**_11_ in (18) shows that to this end $\sqrt{h(x)}x^{a-1}$ needs to have a non-negative degree. This requires *p*_1_ ≥ 2 – 2*a*. The formula for ***g***_1_ similarly shows that *h*′(*x*)*x*^*a*–1^, *h*(*x*)*x*^*a*–2^ and *h*(*x*)*x*^2*a*–2^ all need to have a non-negative degree for small *x*. This requires *p*_1_ ≥ 2 – *a*.
- In the non-centered case, in addition to (i) and (ii),
  (iii) (A1) and (A2): Theorem 11 requires *p*_1_ ≥ max{1 – *a*, 1 – *b*} for *b* > 0, or 1 – min_*j*_ *η*_0,*j*_ for *b* = 0.
  (iv) “Controlled **Γ** and ***g*** for ***x***^*b*^”: From the definition of **Γ**_22_ and ***g***_2_ and by the same reasoning as above, $\sqrt{h(x)}x^{b-1}$, *h*′(*x*)*x*^*b*–1^ and *h*(*x*)*x*^*b*–2^ need to be non-negative powers of *x*, thus requiring *p*_1_ ≥ max{2 – *b*, 2 – 2*b*} = 2 – *b*.

The choice of *p*_2_, is only relevant for large data points. Our main consideration is then merely how well **Γ** and ***g*** concentrate on their true population values (Theorem 13). From this perspective, our intuition is that *p*_2_ should be chosen small so that the tails of the distributions of the entries of **Γ** and ***g*** are well-behaved. Thus, we can choose *p*_2_ = 0, in which case *h*(*x*) = min(*x*^*p*_1_^, *c*) is a truncated power.

### 5.5. Tuning Parameter Selection

By treating the unpenalized loss (i.e., *λ* = 0, ***γ*** = 0) as a negative log-likelihood, we may use the extended Bayesian Information Criterion (eBIC) to choose the tuning parameter (Chen and Chen, 2008; Foygel and Drton, 2010). Consider the centered case as an example. Let ${\hat{S}}^{\lambda }\equiv \{(i,j)\;:{\hat{\kappa }}_{ij}^{\lambda }\neq 0,i<j\}$, where ${\hat{K}}^{\lambda }$ be the estimate associated with tuning parameter *λ*. The eBIC is then

$$\text{eBIC}(\lambda )=-n\text{vec}(\hat{K}{)}^{⊤}\Gamma (x)\text{vec}(\hat{K})+2ng(x{)}^{⊤}\text{vec}(\hat{K})+|{\hat{S}}^{\lambda }|\;\log\;n+2\;\log\left(\begin{array}{c} p(p-1)∕2\\ |{\hat{S}}^{\lambda }| \end{array}\right),$$

where $\hat{K}$ can be either the original estimate associated with *λ*, or a refitted solution obtained by restricting the support to ${\hat{S}}^{\lambda }$.

We use the eBIC instead of the ordinary BIC (Bayesian Information Criterion) since the BIC tends to choose an overly complex model when the model space is large, as encountered in the high-dimensional setting. The extension in eBIC comes from the last term in the above display which can be motivated by a prior distribution under which the number of edges in the conditional independence graph is uniformly distributed; see also Żak-Szatkowska and Bogdan (2011) and Barber and Drton (2015).

## 6. Theory for Graphical Models

In our regularized generalized score matching framework, we introduced the amplifiers/multipliers to address the inexistence problem. We also proposed using a general function ***h*** in place of ***x***^2^ as a means to improve estimation accuracy. This section provides a theoretical analysis of these two aspects.

In Section 6.1, we present the theory for our regularized generalized score matching estimators for general pairwise interaction models before going into the details for the special cases of (truncated) GGMs. Next, we show that a specific choice of amplifiers/multipliers yields consistent estimation without the need for tuning. This point is important even in the case of Gaussian models on all of $R^{m}$. Therefore, in Section 6.2 we digress from non-negative data and consider the original score matching of Hyvärinen (2005) for centered Gaussian distributions. Finally, in Section 6.3, we derive probabilistic results for $\hat{\Psi }$ based on Theorem 13, justifying the benefits of using a general bounded ***h*** over ***x***^2^ in the non-negative setting. As the most important models from the class of pairwise interaction power models over $R_{+}^{m}$, we only treat truncated GGMs since they have the most tractable concentration bounds; this case also provides a comparison to Corollary 2 in Lin et al. (2016), which uses ***x***^2^.

### 6.1. Theory for Pairwise Interaction Models

The graphical models we treat are parametrized by the interaction matrix **K** and the coefficients ***η*** on (***x***^*b*^ – **1**_*m*_)/*b*. It is convenient to accommodate this setting with a matrix-valued parameter $\Psi \in R^{r_{1}\times r_{2}}$ (in place of ***θ***) and specify our regularized ***h***-score matching loss as

(24) $${\hat{J}}_{h,\lambda ,\gamma }(\Psi )\equiv \underset{\Psi \in R^{r_{1}\times r_{2}}}{\text{argmin}}\frac{1}{2}\text{vec}(\Psi {)}^{⊤}\Gamma_{\gamma }(x)\text{vec}(\Psi )-g(x{)}^{⊤}\text{vec}(\Psi )+\lambda \|\Psi {\|}_{1}.$$

In the non-centered case we thus take $\Psi =[K,\eta {]}^{⊤}\in R^{m(m+1)\times m}$. in the centered case, **Ψ** is simply the *m* × *m* interaction matrix **K**. Following related prior work such as Lin et al. (2016), for ease of proof we allow the matrix **K** to be nonsymmetric, which allows us to decouple optimization over the different columns of **K** or **Ψ**, while in our implementations we ensure that **K** is symmetric.

**Definition 12** *Let* $\Gamma_{0}\equiv E_{0}\Gamma (x)$ *and* $g_{0}\equiv E_{0}g(x)$ *be the population versions of* **Γ**(**x**) *and* ***g***(**x**) *under the distribution given by a true parameter matrix* **Ψ**_0_. *The support of a matrix* **Ψ** *is S*(**Ψ**) = {(*i*,*j*) : *ψ*_*ij*_ ≠ 0}, *and we let S*_0_ = *S*(**Ψ**_0_). *For a matrix* **Ψ**_0_, *we define d*_**Ψ**_0__ *to be the maximum number of non-zero entries in any column, and c*_**Ψ**_0__ ≡ ⫼**Φ**_0_⫼_∞,∞_. *Writing* **Γ**_0,*AB*_ *for the A* × *B submatrix of* **Γ**_0_*, we define*

(25) $$c_{\Gamma_{0}}\equiv \||(\Gamma_{0,S_{0}S_{0}}{)}^{-1}|{\|}_{\infty ,\infty }.$$

*Finally*, **Γ**_0_ *satisfies* the irrepresentability condition with incoherence parameter *α* ∈ (0, 1] and edge set *S*_0_ *if*

(26) $$\||\Gamma_{0,S_{0}^{c}S_{0}}(\Gamma_{0,S_{0}S_{0}}{)}^{-1}|{\|}_{\infty ,\infty }\leq (1-\alpha ).$$

Our analysis of the regularized generalized ***h***-score matching estimator builds on the following theorem taken from Lin et al. (2016, Theorem 1).

**Theorem 13** *Suppose* **Γ**_0_ *has* **Γ**_0,*S*_0_*S*_0__ *invertible and satisfies the irrepresentability condition (26) with incoherence parameter α* ∈ (0, 1]. *Assume that*

(27) $$\|\Gamma_{\gamma }(x)-\Gamma_{0}{\|}_{\infty }<\epsilon_{1},\;\;\;\;\;\|g(x)-g_{0}{\|}_{\infty }<\epsilon_{2},$$

*with d*_**Ψ**_0__ *ϵ*_1_ ≤ α/(6*c*_**Γ**_0__). *If*

$$\lambda >\frac{3(2-\alpha )}{\alpha }\;\max\{c_{\Psi_{0}}\epsilon_{1},\epsilon_{2}\},$$

*then the following holds:*

- *The regularized generalized **h**-score matching estimator* $\hat{\Psi }$ *minimizing (24) is unique, with support* $\hat{S}\equiv S(\hat{\Psi })\subseteq S_{0}$*, and satisfies*
$$\|\hat{\Psi }-\Psi_{0}{\|}_{\infty }\leq \frac{c_{\Gamma_{0}}}{2-\alpha }\;\lambda .$$
- If

$$\underset{1\leq j<k\leq m}{\text{min}}|\Psi_{0,jk}|>\frac{c_{\Gamma_{0}}}{2-\alpha }\;\lambda ,$$

*then* $\hat{S}=S_{0}$ *and* $\text{sign}({\hat{\Psi }}_{jk})=\text{sign}(\Psi_{0.jk})$ *for all* (*j*, *k*) ∈ *S*_0_.

This result is deterministic, and the improvement of our generalized estimator over the one in Lin et al. (2016) is in its probabilistic guarantees, as shown for truncated GGMs in Theorems 16 and 17 in Section 6.3. Before going into these examples, we state a general corollary.

**Corollary 14** *Under the assumptions of Theorem 13, the matrix* $\hat{\Psi }$ *minimizing (24) satisfies*

$$\|\hat{\Psi }-\Psi_{0}{\|}_{F}\leq \frac{c_{\Gamma_{0}}}{2-\alpha }\;\lambda \sqrt{|S_{0}|}\leq \frac{c_{\Gamma_{0}}}{2-\alpha }\;\lambda \sqrt{d_{\Psi_{0}},m},\;\|\hat{\Psi }-\Psi_{0}{\|}_{2}\leq \frac{c_{\Gamma_{0}}}{2-\alpha }\;\lambda \;\text{min}(\sqrt{|S_{0}|},d_{\Psi_{0}}).$$

### 6.2. Revisiting Gaussian Score Matching

In this section we consider estimating the inverse covariance matrix **K** of a centered Gaussian distribution N(**0**, **K**), which of course has density proportional to (2) on all of $R^{m}$. As shown, e.g., in Example 1 of Lin et al. (2016), the *ℓ*_1_-regularized score matching loss then takes the form

(28) $$\frac{1}{2}\text{tr}(\mathrm{KK}{\mathrm{xx}}^{⊤})-\text{tr}(K)+\lambda \|K{\|}_{1},$$

which can be written as (14) with ***θ*** = vec(**K**), **Γ** = diag(**xx**^⊤^,…, **xx**^⊤^) and ***g*** = vec(**I**_*m*_). Thus, in general, the kernel of **Γ** need not be orthogonal to ***g***, and for *λ* small the loss can be unbounded below as discussed above. Hence, an amplifier/multiplier on the diagonals of **Γ** is needed. We have the following theorem on the estimator using the amplification.

**Theorem 15** *Suppose the data matrix* **x** *holds n i.i.d. copies of **X*** ~ N(**0**, **K**_0_). *Adopt the amplifying in Section 4 and redefine the loss in (28) as*

(29) $$\frac{1}{2}\text{tr}(\mathrm{KKG})-\text{tr}(K)+\lambda \|K{\|}_{1},\;\;G_{jk}=({\mathrm{xx}}^{⊤}{)}_{jk}\;(1_{\left\{j\neq k\right\}}+(\delta -1)1_{\left\{j=k\right\}}),$$

*where* $1<\delta <2-{\left(1+80\sqrt{\log\;m∕n}\right)}^{-1}$. *Let* $\hat{K}$ *be the resulting estimator. Let* c* ≡ 12800 (max_*j*_ **Σ**_0,*jj*_)^2^ *and c*_1_ = 4*c*_**Γ**_0__/*α*. *If for some τ* > 2*, the regularization parameter and the sample size satisfy*

$$\lambda >(2c_{K_{0}}(2-\alpha )\sqrt{c^{*}(\tau \;\log\;m+\log\;4)∕n)}∕\alpha ,\;n>\max(c^{*}c_{1}^{2}d_{K_{0}}^{2},2)(\tau \;\log\;m+\log\;4),$$

*then* $\|\hat{K}-K_{0}{\|}_{\infty }\leq \frac{c_{\Gamma_{0}}}{2-\alpha }\lambda$ *with probability* 1 – *m*^2–*τ*^.

In Corollary 1 of Lin et al. (2016) the same results were shown with c* ≡ 3200 (max_*j*_ **Σ**_0,*jj*_)^2^ when a unique minimizer exists, but the existence was not guaranteed.

### 6.3. Generalized Score Matching for Truncated GGMs

Next, we provide theory for the regularized generalized ***h***-score matching estimator $\hat{\Psi }$ in the special case of truncated GGMs. Again, assume a common *h* for all components in ***h***.

**Theorem 16** *Suppose the data matrix* **x** *holds n i.i.d. copies of **X*** ~ TN(**0**, **K**_0_)*, where the mean parameter is known to be zero. Assume that* $h\in H_{1,1}$ *and that* 0 ≤ *h* ≤ *M*, 0 ≤ *h*′ ≤ *M*′ *a.s. for constants M, M′, and choose γ* = (*δ* – 1)diag(**Γ**) *with*

$$1<\delta <C(n,m)\equiv 2-{\left(1+4e\;\max\{6\;\log\;m∕n,\sqrt{6\;\log\;m∕n}\}\right)}^{-1}.$$

*Suppose that the* **Γ**_0,*S*_0_*S*_0__ *block of* **Γ**_0_ *is invertible and* **Γ**_0_ *satisfies the irrepresentability condition (26) with α* ∈ (0, 1] *and true edge set S*_0_. *Define* $c_{X}\equiv 2\;\max_{j}\left(2\sqrt{(K_{0}^{-1}{)}_{jj}}+\sqrt{e}\;E_{0}X_{j}\right)$. *If for τ* > 3 *the sample size and the regularization parameter satisfy*

(30) $$n>O\left(\tau \;\log\;m\;\max\left\{\frac{M^{2}c_{\Gamma_{0}}^{2}c_{X}^{4}d_{K_{0}}^{2}}{\alpha^{2}},\frac{Mc_{\Gamma_{0}}c_{X}^{2}d_{K_{0}}}{\alpha }\right\}\right),$$

(31) $$\lambda >O\left((Mc_{K_{0}}c_{X}^{2}+M^{\prime }c_{X}+M)\left(\sqrt{\frac{\tau \;\log\;m}{n}}+\frac{\tau \;\log\;m}{n}\right)\right],$$

*then the following statements hold with probability* 1 – *m*^3–*τ*^:

- *The regularized generalized **h**-score matching estimator* $\hat{K}$ *that minimizes (24) is unique, has its support included in the true support,* $\hat{S}\equiv S(\hat{K})\subseteq S_{0}$*, and satisfies*
$$\|\hat{K}-K_{0}{\|}_{\infty }\leq \frac{c_{\Gamma_{0}}}{2-\alpha }\;\lambda ,$$
$$\||\hat{K}-K_{0}|{\|}_{F}\leq \frac{c_{\Gamma_{0}}}{2-\alpha }\;\lambda \sqrt{|S_{0}|},\;\||\hat{K}-K_{0}|{\|}_{2}\leq \frac{c_{\Gamma_{0}}}{2-\alpha }\;\lambda \;\text{min}(\sqrt{|S_{0}|},d_{K_{0}}),$$
  *where c*_**Γ**_0__ *is defined in* (25).
- *Moreover, if*
$$\underset{j,k:(j,k)\in S_{0}}{\text{min}}|\kappa_{0,jk}|>\frac{c_{\Gamma_{0}}}{2-\alpha }\;\lambda ,$$
  *then* $\hat{S}=S_{0}$ *and* $\text{sign}({\hat{\kappa }}_{jk})=\text{sign}(\kappa_{0,jk})$ *for all* (*j*, *k*) ∈ *S*_0_.

The theorem is proved in Appendix A.4, where details on the dependencies on constants are provided. A key ingredient of the proof is a tail bound on ∥**Γ**_***γ***_ – **Γ**_0_∥_∞_, which features products of the $X_{j}^{\left(i\right)}$. In Lin et al. (2016), the products are up to fourth order. Using bounded ***h***, our products automatically calibrates to a quadratic polynomial when the observed values are large, and resort to higher moments only when they are small. This leads to improved bounds and convergence rates, underscored in the new requirement on the sample size *n*, which should be compared to $n\geq O(d_{K_{0}}^{2}(\log\;m^{\tau }{)}^{8})$ in Lin et al. (2016).

For the non-centered case, by definition, *c*_**Ψ**_0__ ≡ ⫼**Ψ_0_**^⊤^⫼_∞,∞_ ≤ *c*_**K**_0__ + ∥***η***_0_∥∞, *d*_**Ψ**_0__ ≤ *d*_**K**_0__ + 1. The proof given for Theorem 16 goes through again here, and we have the following consistency results.

**Theorem 17** *Suppose the data matrix holds n i.i.d. copies of **X*** ~ TN(***μ***_0_, **K**_0_). *Assume that* $h\in H_{1,1}$ *and that* 0 ≤ *h* ≤ *M*, 0 ≤ *h*′ ≤ *M*′ *a.s. for constants M, M′. Let **γ** be a vector of amplifiers that are non-zero only for the diagonal entries of the matrices* **Γ**_11,*j*_*, amplifying those by* (*δ* – 1)diag(**Γ**_11,*j*_) *with*

$$1<\delta <C(n,m)\equiv 2-{\left(1+4e\;\max\{6\;\log\;m∕n,\sqrt{6\;\log\;m∕n}\}\right)}^{-1}.$$

*Suppose further that* **Γ**_0,*S*_0_*S*_0__ *is invertible and satisfies the irrepresentability condition (26) with α* ∈ (0, 1]. *Define* $c_{X}\equiv 2\;\max_{j}\left(2\sqrt{(K_{0}^{-1}{)}_{jj}}+\sqrt{e}\;E_{0}X_{j}\right)$. *Suppose for τ* > 3 *the sample size and the regularization parameter satisfy*

(32) $$n>O\left(\tau \;\log\;m\;\max\left\{\frac{M^{2}c_{\Gamma_{0},\Psi_{0}}^{2}c_{X}^{4}d_{\Psi_{0}}^{2}}{\alpha^{2}},\frac{Mc_{\Gamma_{0},\Psi_{0}}c_{X}^{2}d_{\Psi_{0}}}{\alpha }\right\}\right),$$

(33) $$\lambda >O\left((Mc_{\Psi_{0}}c_{X}^{2}+M^{\prime }c_{X}+M)\left(\sqrt{\frac{\tau \;\log\;m}{n}}+\frac{\tau \;\log\;m}{n}\right)\right],$$

*where* *c*_**Γ**_0_,**Ψ**_0__ *is c*_**Γ**_0__ *as in (25) but with notation* **Ψ**_0_ *to differentiate it from the centered case. Then the following statements hold with probability* 1 – *m*^3–*τ*^:

- *The regularized generalized **h**-score matching estimator* $\hat{\Psi }$ *that minimizes (24) is unique, has its support included in the true support,* $\hat{S}\equiv S(\hat{\Psi })\subseteq S_{0}$, *and satisfies*
$$\|\hat{K}-K_{0}{\|}_{\infty }\leq \frac{c_{\Gamma_{0},\Psi_{0}}}{2-\alpha }\;\lambda \;\;\;\;\;\;\;\;\|\hat{\eta }-\eta_{0}{\|}_{\infty }\leq \frac{c_{\Gamma_{0},\Psi_{0}}}{2-\alpha }\;\lambda ,$$
$$\||\hat{K}-K_{0}|{\|}_{F}\leq \frac{c_{\Gamma_{0},\Psi_{0}}}{2-\alpha }\;\lambda \sqrt{|S_{0}|},\;\;\;\;\;\;\;\||\hat{\eta }-\eta_{0}|{\|}_{F}\leq \frac{c_{\Gamma_{0},\Psi_{0}}}{2-\alpha }\;\lambda \sqrt{|S_{0}|},\;\||\hat{K}-K_{0}|{\|}_{2}\leq \frac{c_{\Gamma_{0},\Psi_{0}}}{2-\alpha }\;\lambda \;\text{min}\left(\sqrt{|S_{0}|},d_{\Psi_{0}}\right),\;\;\||\hat{\eta }-\eta_{0}|{\|}_{2}\leq \frac{c_{\Gamma_{0},\Psi_{0}}}{2-\alpha }\;\lambda \;\text{min}\left(\sqrt{|S_{0}|},d_{\Psi_{0}}\right).$$
- *Moreover, if*
$$\underset{j,k:(j,k)\in S_{0}}{\text{min}}|\kappa_{0,jk}|>\frac{c_{\Gamma_{0}}}{2-\alpha }\;\lambda \;\;\;and\;\;\;\underset{j:(m+1,j)\in S_{0}}{\text{min}}|\eta_{0,j}|>\frac{c_{\Gamma_{0}}}{2-\alpha }\;\lambda ,$$
  *then* $\hat{S}=S_{0}$ *and* $\text{sign}({\hat{\kappa }}_{jk})=\text{sign}(\kappa_{0,jk})$ *for all* (*j*, *k*) ∈ *S*_0_ *and* $\text{sign}({\hat{\eta }}_{j})=\text{sign}(\eta_{0j})$ *for* (*m* + 1, *j*) ∈ *S*_0_.

**Remark 18** The quantity *c_**X**_* in Theorem 17 depends on $E_{0}X_{j}$, which in turn depends on the structure of both ***μ***_0_ and **K**_0_. If *μ*_0,*j*_ is large compared to $(K_{0}{)}_{jj}^{-1}$, then *c*_***X***_ seems to scale as ***μ***_0_, which negatively impacts the guarantees stated in Theorem 17. However, as in the one-dimensional case for estimation of *μ*_0_ (Example 3.1), our estimator should automatically adapt to the large mean parameter. This suggests that it might be possible to improve our analysis involving *c*_***X***_.

## 7. Numerical Experiments

In this section, we compare the performance of our estimator with different choices of ***h*** to the existing approaches for pairwise interaction power models. In our simulation experiments, we consider *m* = 100 variables and *n* = 80 and *n* = 1000 samples, corresponding to high- and low-dimensional settings. We also tried intermediate sample sizes between these two extremes, but found no interesting result worth reporting. For *n* = 80, amplification is necessary. Except in Section 7.2.2, the amplifier is set based on Theorem 16 to *δ* = *C*(*n*, *m*) = 1.8647 for truncated GGMs. The same amplifier is also used for settings with other *a* and *b*. For *n* = 1000, we consider *δ* = 1, i.e., no amplification, and *δ* = *C*(*n*, *m*) = 1.6438 (again, based on Theorem 16). Throughout, we assume a common univariate *h* for all components in ***h***.

### 7.1. Structure of K

The underlying interaction matrices are selected as follows: Proceeding as in Section 4.2 of Lin et al. (2016), the graph is chosen to have 10 disconnected subgraphs, each containing *m*/10 nodes. Thus, **K**_0_ is block-diagonal. In each block, each lower-triangular element is set to 0 with probability 1 – *π* for some *π* ∈ (0, 1), and is otherwise drawn from Uniform[0.5, 1]. The upper triangular elements are determined by symmetry. The diagonal elements of **K**_0_ are chosen as a common positive value such that the minimum eigenvalue of **K**_0_ is 0.1.

We generate 5 different true precision matrices **K**_0_, and run 10 trials with each of these precision matrices. For *n* = 1000, we choose *π* = 0.8, which is in accordance with Lin et al. (2016). For *n* = 80, we set *π* = 0.2. This way $n∕(d_{K_{0}}^{2}\;\log\;m)$ is roughly constant; recall Theorems 16 and 17 for truncated GGMs.

In Appendix C, we report results on *Erdös-Rényi graphs*, which lead to similar conclusions.

### 7.2. Truncated GGMs

Given our focus on truncated GGMs and their relevance in graphical modeling applications, we start with experiments for these models.

#### 7.2.1. Choice of *h*

Our estimator requires choosing a function ***h*** : $R_{+}^{m}\to R_{+}^{m}$. For simplicity, we will always specify ***h***(***x***) = (*h*(*x*_1_),…, *h*(*x_m_*)) for a single non-decreasing univariate function *h* : $R_{+}\to R_{+}$, i.e. all coordinates share the same *h* function.

As previously explained, $h\in H_{a,b}$ is a sufficient condition for assumptions (A1)-(A2), as well as (C1)-(C2) in the case of unregularized estimators. Only in the proofs of our theoretical guarantees in Section 6 for truncated GGMs, did we require *h* to be bounded and to have bounded derivatives. As motivated by the discussion in Section 5.4.2, we consider truncated and untruncated powers, min(*x*, *c*) and *x* (since 2 – *a* = 2 – *b* = 1); we evaluate this choice by contrasting them with powers *x*^1.5^ and *x*^2^. We also explore functions like log(1 + *x*) that seem natural and are linear near 0. In particular, we make a further comparison to functions linear near 0 with a finite asymptote as *x* ↗ +∞ but differentiable everywhere: MCP- (Fan and Li, 2001) and SCAD-like (Zhang, 2010) functions defined below. The results we report are based on selections of best performing choices of *h*.


SCAD(x;λ,γ)≡{λxif0≤x≤λ,2γλx−x2−λ22(γ−1)ifλ<x<γλ,MCP(x;λ,γ)≡{λx−x22γif0≤x≤γλ,12γλ2ifx>γλ.}λ2(γ+1)2ifx≥γλ;}

We do not observe any clear relationship between features such as convexity, differentiability or the slope of *h* at 0, and performance of the estimator. Nonetheless, for many choices of rather simple functions *h*, our estimator provides a significant improvement over existing methods. In particular, most *h* functions that behave linearly for small *x*, namely log(1 + *x*) and *x* and their truncations, and additionally MCP and SCAD, always perform better than *x*^1.5^ and *x*^2^. This agrees with our discussion in Section 5.4.2, where 2 – *a* = 1 is a reasonable choice of the power for small *x*; also see Section 7.3. However, we conclude that there is no real gain from making the function smoother by using MCP or SCAD.

##### Truncated Centered GGMs:

For data from a truncated centered Gaussian distribution, we compare our generalized score matching estimator with various choices of *h*, to *SpaCE JAM* (SJ, Voorman et al., 2014), which estimates graphs using additive models for conditional means, a pseudo-likelihood method *SPACE* (Peng et al., 2009) in the reformulation of Khare et al. (2015), *graphical lasso* (GLASSO, Yuan and Lin, 2007; Friedman et al., 2008), the *neighborhood selection* estimator (NS) of Meinshausen and Bühlmann (2006), and *nonparanormal SKEPTIC* (Liu et al., 2012) with Kendall’s *τ*. Recall that the choice of *h*(*x*) = *x*^2^ corresponds to the estimator from Lin et al. (2016).

**Figure 3:**
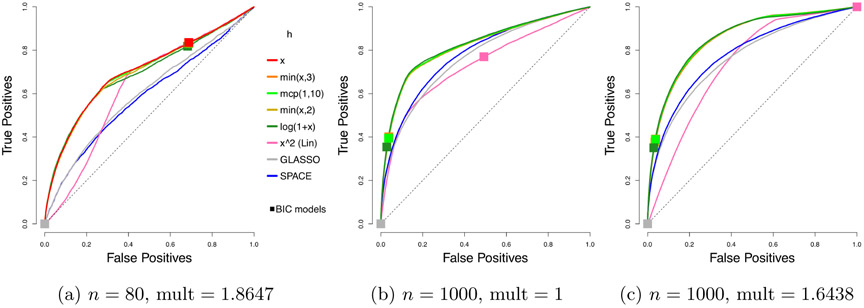
Average ROC curves of our centered estimator with various choices of *h*, compared to SPACE and GLASSO, for the truncated centered GGM case; *m* = 100 variables and *n* = 80 or 1000 samples are considered. Squares indicate average true positive rate (TPR) and false positive rate (FPR) of models picked by eBIC with refitting for the estimator in the same color.

The ROC (*receiver operating characteristic*) curves for different estimators are shown in Figure 3 on Page 26. Each plotted curve corresponds to the average of 50 ROC curves, where the averaging is based on the vertical averaging from Algorithm 3 in Fawcett (2006), and is mean AUC-preserving. The *x* and *y* axes of each ROC curve represent the false positive and true positive rates at varying levels of penalty parameter *λ*, defined as

$$\text{FPR}\equiv \frac{|{\hat{S}}_{\text{off}}\setminus S_{0,\text{off}}|}{m(m-1)-|S_{0,\text{off}}|}\;\;\;\text{and}\;\;\;\text{TPR}\equiv \frac{|{\hat{S}}_{\text{off}}\cap S_{0,\text{off}}|}{|S_{0,\text{off}}|},$$

where *S*_0,off_ = {(*i*, *j*) : *i* ≠ *j* ⋀ *κ*_0,*ij*_ ≠ 0}, and ${\hat{S}}_{\text{off}}\equiv \{(i,j)\;:i\neq j\wedge {\hat{\kappa }}_{ij}\neq 0\}$.

To reduce clutter, we only report the results for the top performing competing methods. In particular, results for nonparanormal SKEPTIC are omitted, as the method always performs the worst in our experiments. The corresponding means and standard deviations of AUCs (*areas under the curves*) over 50 curves are given in Table 1.

Looking at the mean AUCs, with the standard deviations in mind, all choices of *h* considered here perform better than *h*(*x*) = *x*^2^ from Hyvärinen (2007) and Lin et al. (2016) and the competing methods. The results for *n* = 1000 in Table 1 also show that the multiplier does help improve the AUCs, a matter to be discussed in Section 7.2.2.

##### Truncated Non-Centered GGMs:

We generate data from a truncated non-centered Gaussian distribution with both parameters ***μ*** and **K** unknown. In each trial, we form the true **K**_0_ as in Section 7.1, and generate each component of ***μ***_0_ independently from the normal distribution with mean 0 and standard deviation 0.5.

**Figure 4:**
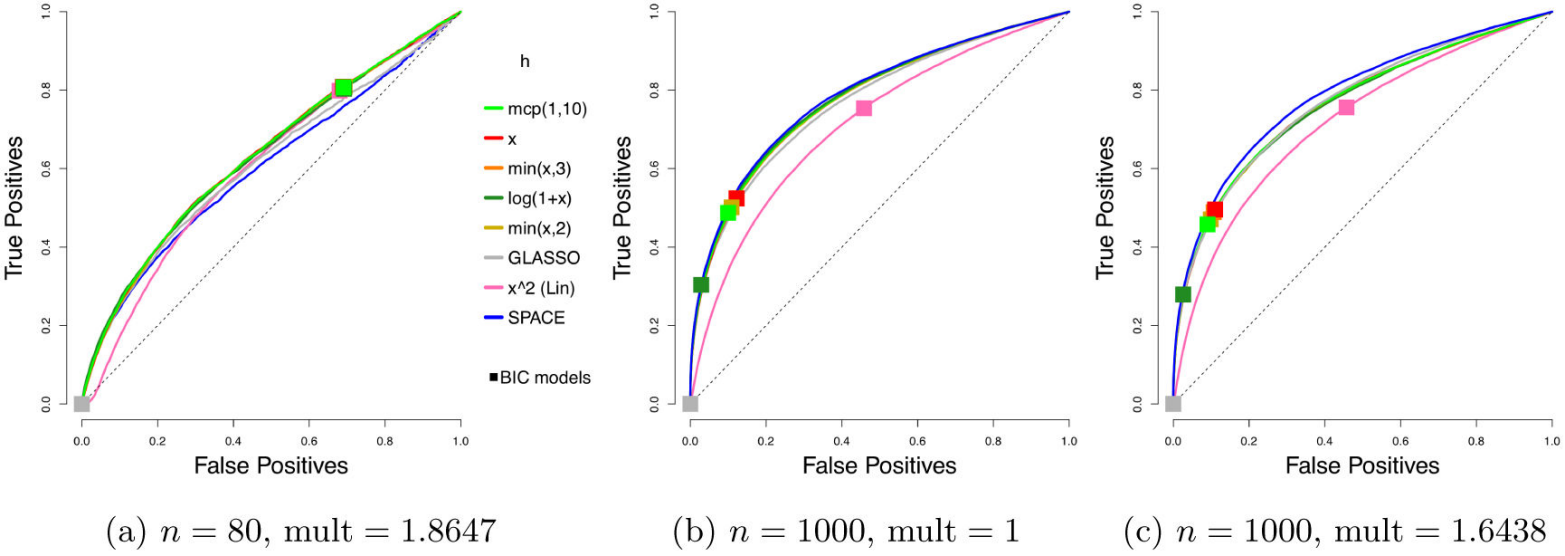
Average ROC curves of our non-centered profiled estimator with various choices of *h*, compared to SPACE and GLASSO, for the truncated non-centered GGM case; *m* = 100 variables and *n* = 80 or 1000 samples are considered. Squares indicate average true positive rate (TPR) and false positive rate (FPR) of models picked by eBIC with refitting for the estimator in the same color.

We compare the performance of our *profiled* estimator based on (19), with different *h* functions, but with no penalty on ***η*** ≡ **K*μ***, to SPACE, SpaCE JAM (SJ), GLASSO, and neighborhood selection (NS). As before, we consider 50 trials. Representative ROC curves are plotted in Figure 4, and the corresponding AUCs are summarized in Table 2.

Even without tuning the extra penalty parameter on ***η*** ≡ **K*μ***, our profiled estimator beats the competing methods by a large margin when *n* = 80. With multipliers 1 and *n* = 1000, our estimators still do better than Space JAM and GLASSO, and have performance comparable to other competing methods. It might appear that the performance of our estimators deteriorate with a multiplier larger than 1; however, as we will see, there can be significant improvement in AUCs if we tune an additional parameter for the multiplier. As in the centered case, the leading *h* functions in each category perform similarly, and the exact choice is not crucial. Subsequently, we will simply use *h*(*x*) = min(*x*, 3).

#### 7.2.2. Choice of multiplier

##### Truncated Centered GGMs:

**Figure 5:**
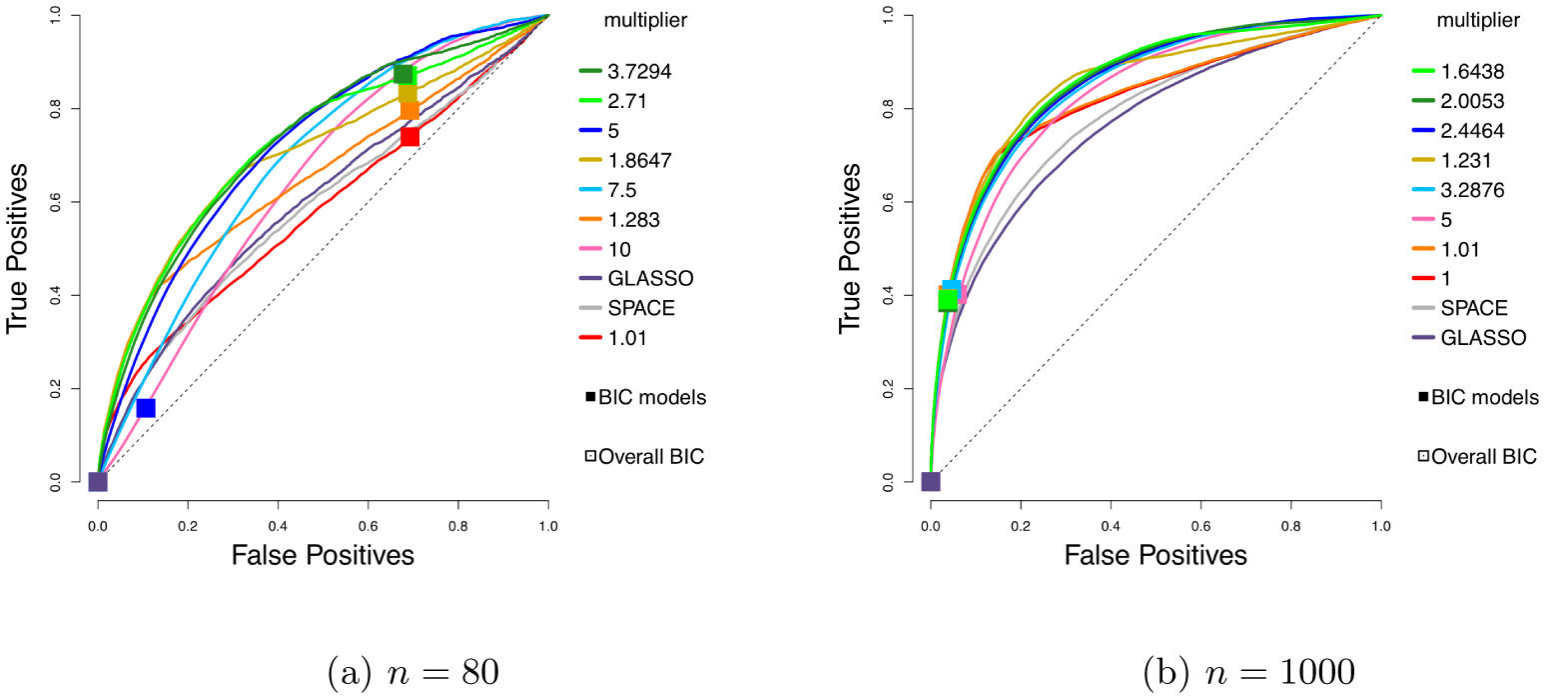
Performance of min(*x*, 3) for truncated centered GGMs using different multipliers, compared to GLASSO and SPACE, in the centered setting, *n* = 80 or 1000.

In Figure 5, the ROC curves for GLASSO, SPACE, and our estimator with *h*(*x*) = min(*x*, 3), but with different levels of amplification, via different choices of multipliers *δ*, are compared for the centered case of Section 7.2.1.

While Theorem 16 guarantees consistency only for *δ* < *C*(*n*, *m*), we observe that there can be a gain from going beyond the *upper-bound multiplier C*(*n*, *m*), which is 1.8647 for *n* = 80 and 1.6438 for *n* = 1000 (when *n* = 1000, *C*(*n*, *m*) turns out to be the best-performing multiplier). However, the effect deteriorates fast as the multiplier grows larger. The figure suggests that while some additional gains are possible by tuning over the choice of multiplier, the *upper-bound multiplier* is a good default.

##### Truncated Non-Centered GGMs:

**Figure 6:**
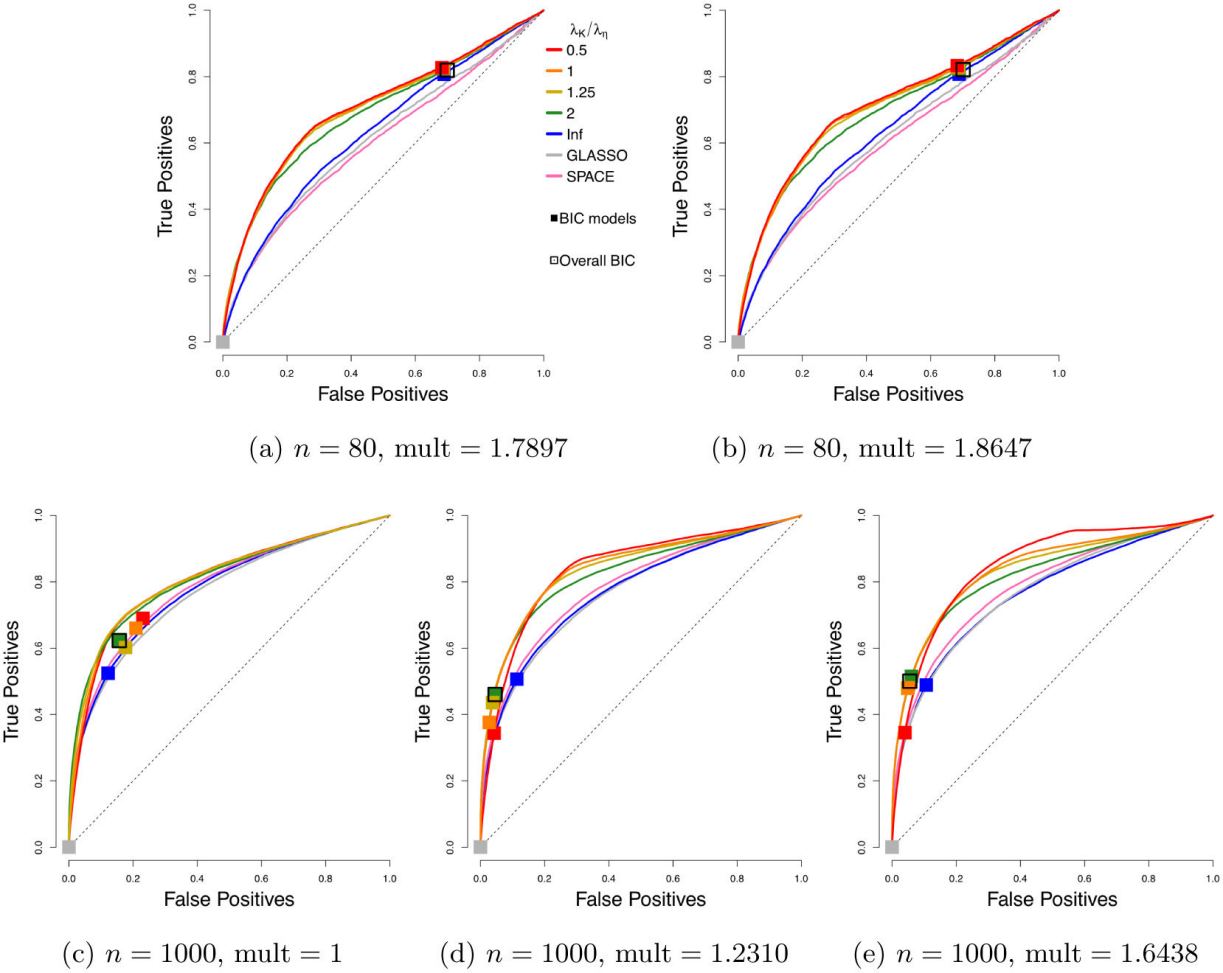
Performance of the non-centered estimator with *h*(*x*) = min(*x*, 3). Each curve corresponds to a different choice of *λ*_**K**_/*λ*_***η***_. Squares indicate models picked by eBIC with refit. The square with black outline has the highest eBIC among all models (combinations of *λ*_**K**_, *λ*_***η***_). Multipliers correspond to medium or high for *n* = 80, and low, medium or high for *n* = 1000, respectively.

In Figure 6, we consider the non-centered case of Section 7.2.1, and use the non-profiled estimator; that is, the non-centered estimator with *ℓ*_1_ penalty on both **K** and ***η*** ≡ **K*μ***. The ROC curves are compared to competing methods GLASSO and SPACE. For the choice of amplification in our estimator, we consider the upper-bound multiplier *C*(*n*, *m*) from Theorem 17 as the default. We refer to this as *high* amplification. We also consider lower amplification, with *δ* = 2 – (1 + 24*e* log *m*/*n*)^−1^, re ferred to as *medium*. For *n* = 1000, we also consider a *low* multiplier 1, which corresponds to no amplification. We compare these possible defaults to a finer grid of multipliers of which we show some representatives in the plots.

We see that among our defaults, the upper-bound choice *C*(*n*, *m*) performs best. Some additional gains are possible by tuning the multiplier over a grid of values containing this choice. Moreover, we see that it can be beneficial to tune over both *λ*_**K**_ and *λ*_**K**_/*λ*_**η**_.

We remark that while for each run, the best model picked by BIC falls on the ROC curve, a few squares are off the curve in Figure 6 (c). This is because these squares correspond to the average of the true and false positive rates of the chosen BIC models over 50 runs, potentially due to multimodality of the distribution of the models. Nonetheless, in all cases, the average of the models picked by BIC tuned over both *λ*_**K**_ and *λ*_**K**_/*λ*_***η***_ looks reasonable.

### 7.3. Other *a/b* Models

We now turn to the non-Gaussian (*a* ≠ 1 or *b* ≠ 1) setting. Based on the observations in Section 5.4.2, we focus on functions of type min(*x^p^*, *c*) for some power *p* > 0 and truncation point *c* > 0. For simplicity, for the non-centered models we use the profiled estimator (19) (i.e., *λ*_***η***_ = 0) and use the multiplier *C*(*n*, *m*) in Theorem 16 for truncated GGMs as a guidance. We note that tuning over the *λ*_***η***_ parameter and the multiplier can potentially give a significant improvement as seen in Section 7.2.

These simulations suggest that among the class of functions of the form min(*x^p^*, *c*), *x*^2–*a*^ or min(*x*^2–*a*^, *c*) with a moderately large *c* can be used as the default choice of *h*(*x*). This agrees with our findings in Section 7.2.1. We note that bounded *h* functions were only used in the proof for truncated GGMs, and picking a moderately large truncation point can correspond to having an untruncated power.

#### 7.3.1. Exponential Setting

For the exponential models, *a* = *b* = 1/2. Since *a* = *b*, for both centered and non-centered settings, based on the principle in Section 5.4.2, choosing *h*(*x*) = min(*x*^3/2^, *c*) satisfies (A1) and (A2) and also ensures that entries in **Γ** and ***g*** are bounded (for small *x*), while choosing $h(x)=\text{min}(\sqrt{x},c)$ only guarantees (A1) and (A2).

**Figure 7:**
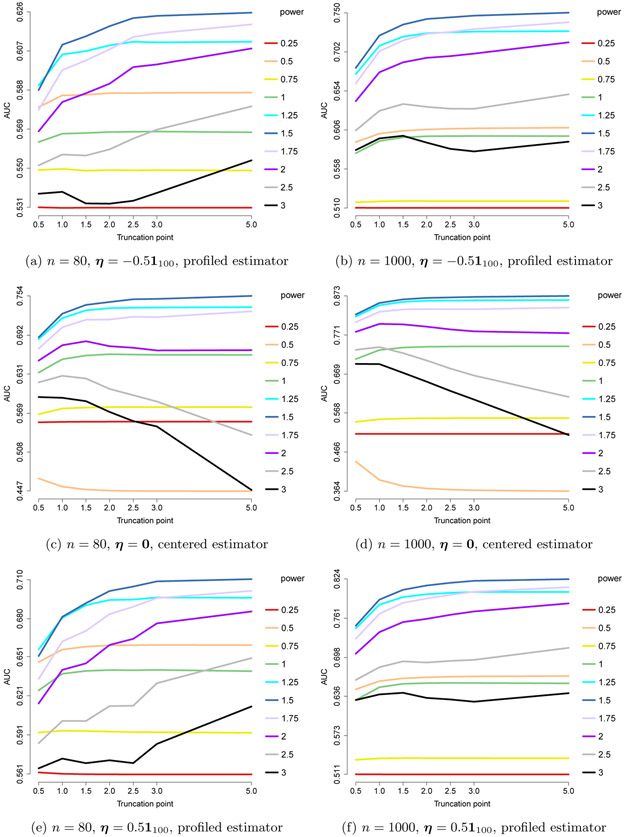
AUCs for edge recovery using generalized score mulching for the exponential models. Each curve represents a different choice of power *p* in *h*(*x*) = min(*x^p^*, *c*), and the *x* axis marks the Iruncalion point *c*. Colors are sorted by *p*.

In Figure 7, we present the AUCs for the ROC curves of edge recovery with different choices of *h*(*x*) = min(*x*^pow^, *c*). As before, we set *n* = 80 or 1000 and *m* = 100, but we use an ***η***_0_ with each component uniformly equal to −0.5, 0 or 0.5; for ***η***_0_ ≡ **0**, we assume this information is known and use the centered estimator. The results suggest that pow = 3/2 = 2 – *a* is the best choice of power. For this optimal choice, the performance improves with larger *c*, so *x*^2–*a*^ gives the best results. For sub-optimal powers, including truncation gives better results.

#### 7.3.2. Gamma Setting

The centered gamma models reduce to the centered exponential models. Thus, in this section, we only consider the non-centered settings, with *a* = 1/2, *b* = 0. From Section 5.4.2, we have the following choices:

- min(*x*^2^, *c*) both satisfies (A1)-(A2) and ensures **Γ** and ***g*** are bounded;
- min(*x*^max{3/2,1–min_*j*_ *η*_0,*j*_}^, *c*) ensures (A1)-(A2) and bounds **Γ**_11_ and ***g***_1_; by default without prior information on ***η***_0_ this is min(*x*^2^, *c*);
- min(*x*^3/2^, *c*) satisfies both conditions on the interaction part only (***x***^*a*^), but does not guarantee (A1)-(A2);
- min(*x*^1/2^, *c*) satisfies the sufficient conditions for (A1)-(A2) on the interaction only.

**Figure 8:**
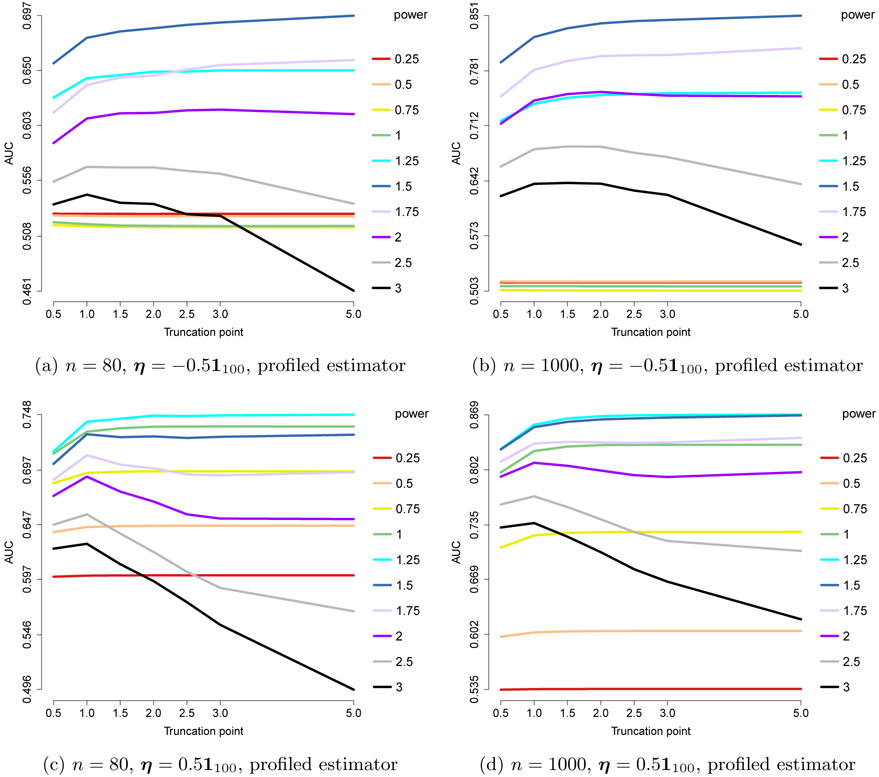
AUCs for edge recovery using generalized score matching for the gamma models. Each curve represents a different choice of power *p* in *h*(*x*) = min(*x^p^*, *c*), and the *x* axis marks the truncation point *c*. Colors are sorted by *p*.

The results are shown in Figure 8, where we consider *n* = 80, 1000, and ***η*** = ±0.5**1**_100_. They suggest that pow = 2 – *a* = 1.5 works consistently well, although slightly outperformed by 1 and 1.25 in one case. As in the exponential case, with the optimal power it is beneficial to choose a large truncation point, or work with an untruncated power *x*^1.5^. We conclude that the performance is likely only dependent on the (2 – *a*) power requirement for the ***x***^*a*⊤^**K*x***^*a*^ part or 2 – min_*j*_ *η*_0,*j*_; simulations in the next section rule out the possibility of the latter.

#### 7.3.3. Other Choices of *a* and *b*

In this section, we consider other choices of *a* and *b*. Specifically, *a* = 3/2 and *b* = 1/2 or 0. These combinations are chosen to confirm, in a more extreme setting, that the performance is mainly determined by requirements on the power based on *a*, which correspond to choosing a power of 1 – *a* or 2 – *a*, but not those on *b* (or on ***η*** when *b* = 0) that correspond to 1 – *b* and 2 – *b*. The relationship between these two settings is analogous to that between the exponential and gamma models (same *a, b* nonzero/zero).

**Figure 9:**
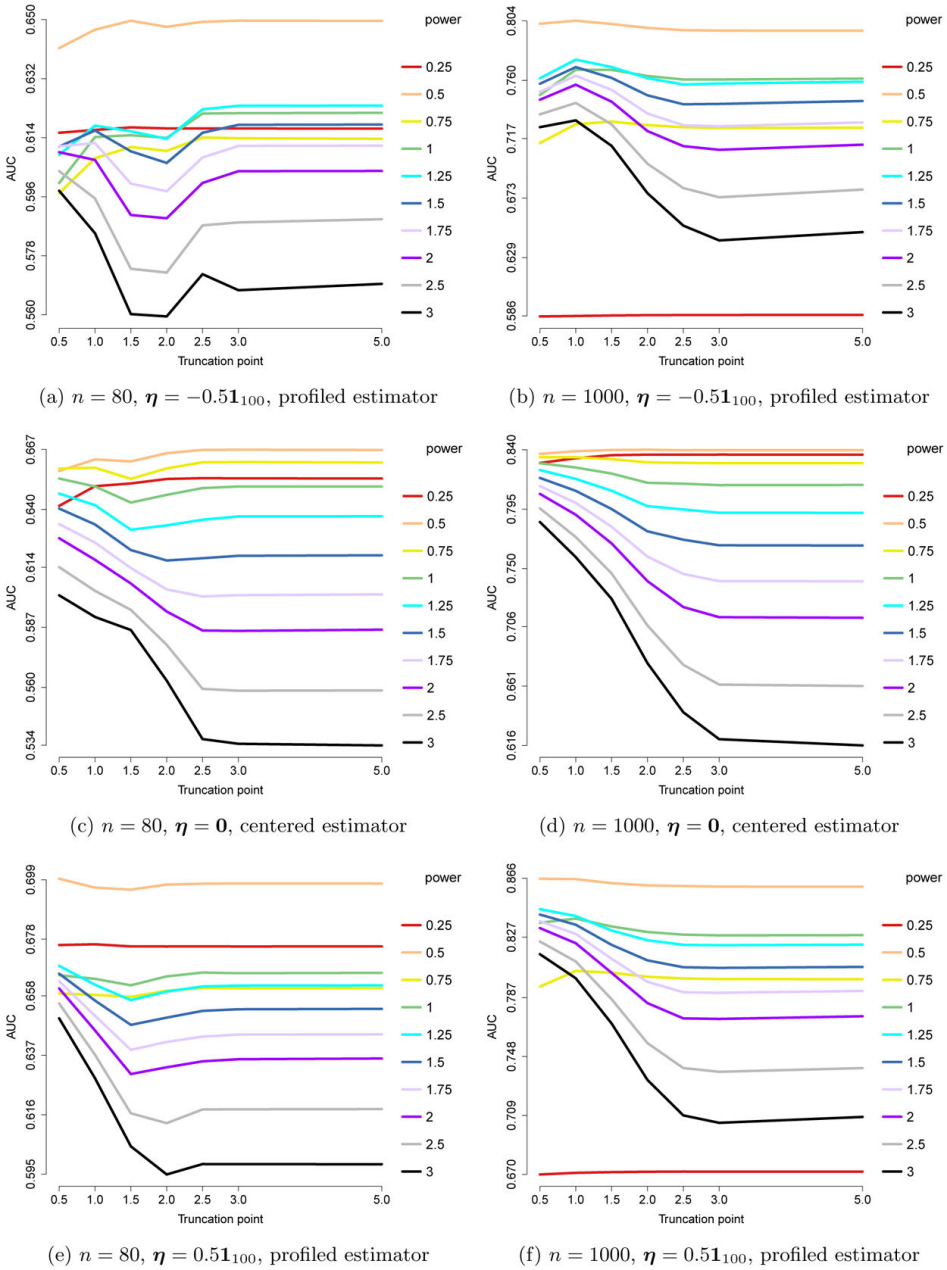
AUCs for edge recovery using generalized score matching for *a* = 3/2, *b* = 1/2. Each curve represents a different choice of power *p* in *h*(*x*) = min(*x^p^*, *c*), and the *x* axis marks the truncation point *c*. Colors are sorted by *p*.

**Figure 10:**
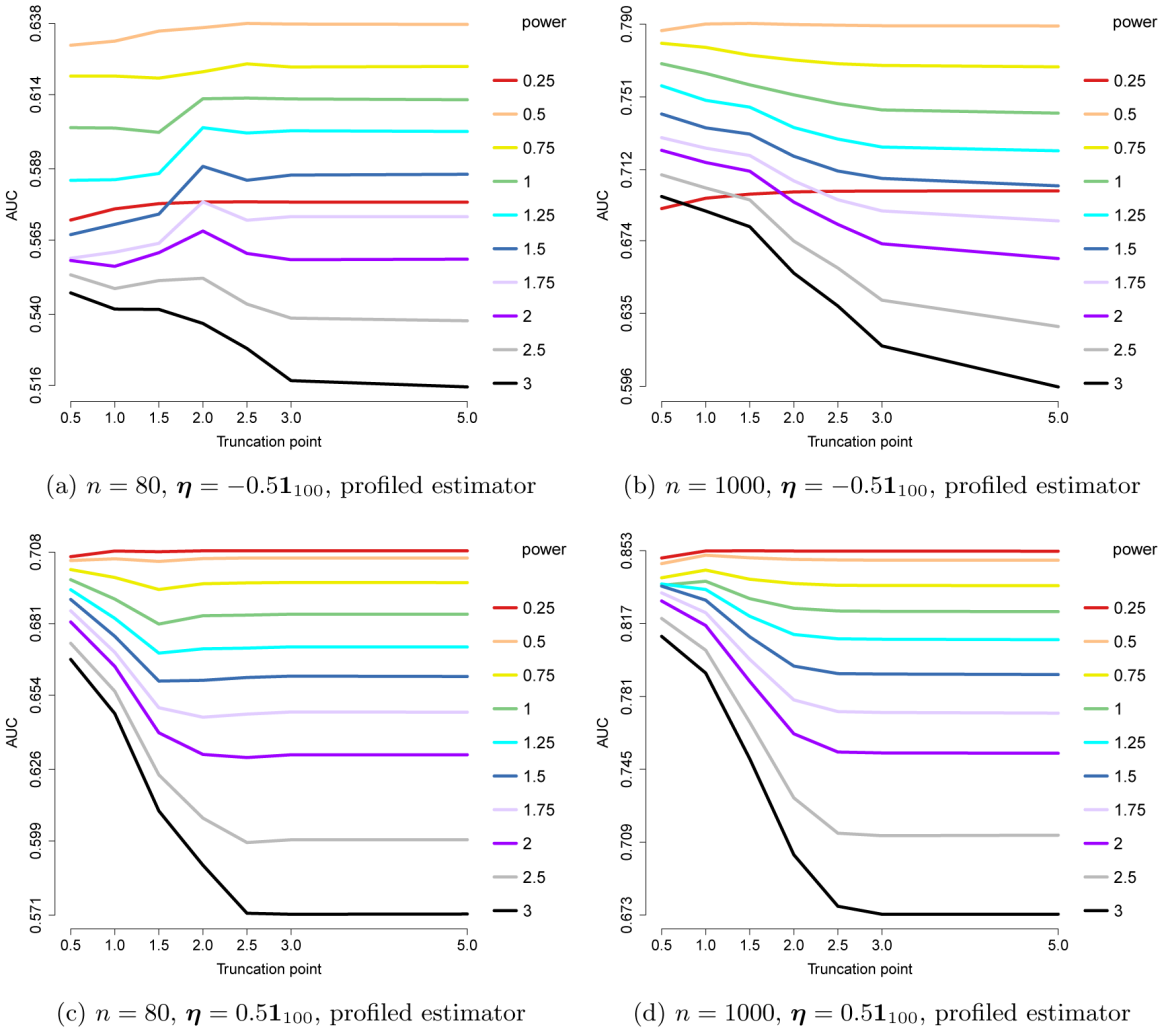
AUCs for edge recovery using generalized score matching for *a* = 3/2, *b* = 0. Each curve represents a different choice of power *p* in *h*(*x*) = min(*x^p^*, *c*), and the *x* axis marks the truncation point *c*. Colors are sorted by *p*.

The results are shown in Figures 9 and 10, and indeed confirm that *x*^2–*a*^ = *x*^0.5^ consistently gives the optimal results, even though ***η***^⊤^***x***_*b*_ is in favor of *x*^2–*b*^ = *x*^1.5^ for *b* = 0.5, and ***η***^⊤^ log(***x***) is in favor of *x*^2^ or at least *x*^1–min_*j*_ *η*_0,*j*_^ when *b* = 0. There are two possible explanations for the optimality of 2 – *a* over max{2 – *a*, 2 – *b*} or max{2 – *a*, 1 – min_j_ *η*_0,*j*_}: (1) The AUC metric is measured only on our interest, edge recovery for the interaction matrix, which only depends on ***x***^*a*^; (2) using the profiled estimator weakens the effect of *b*.

### 7.4. RNAseq Data

In this section we apply our regularized generalized ***h***-score matching estimator for truncated non-centered GGMs to RNAseq data also studied in Lin et al. (2016), since the same model is considered therein. The data consists of *n* = 487 prostate adenocarcinoma samples from The Cancer Genome Atlas (TCGA) data set. Following Lin et al. (2016), we focus on *m* = 333 genes that belong to the known cancer pathways in the Kyoto Encyclopedia of Genes and Genomes (KEGG) and that have no more than 10% missing values. Missing values are set to 0. We choose *h*(*x*) = min(*x*, 3) and use the upper-bound multiplier (*high*), as discussed in Section 7.2.2. For simplicity, we use the profiled estimator, and choose the regularization parameter *λ* so that the estimated graph has exactly *m* = 333 edges, all these choices being as in Lin et al. (2016).

**Figure 11:**
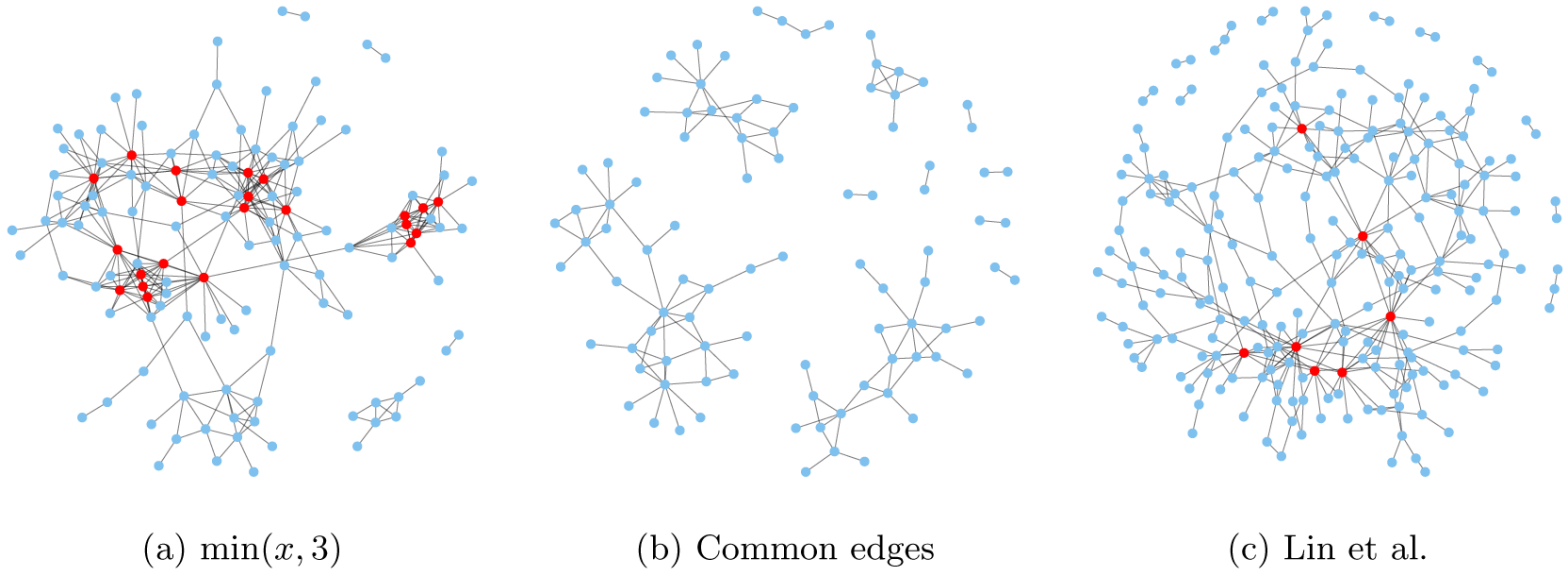
Graphs estimated by regularized generalized score matching estimator with *h*(*x*) = min(*x*, 3) with upper-bound multiplier (left) and *h*(*x*) = *x*^2^ with no multiplier (Lin et al., 2016, right), and their intersection graph (middle). Isolated nodes with no edges are removed, and the layout is optimized for each plot. In (a) and (c), red points indicate nodes with degree at least 10 (“hub nodes”).

**Figure 12:**
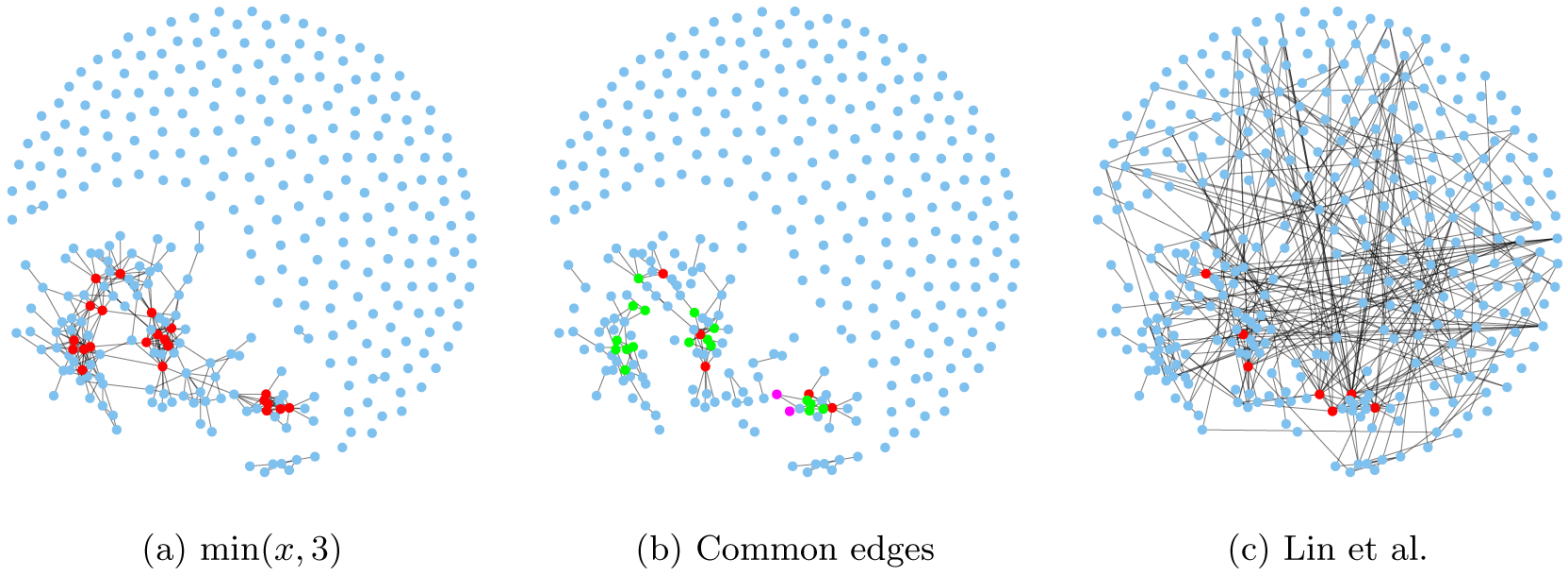
Graphs estimated by regularized generalized score matching estimator with *h*(*x*) = min(*x*, 3) with upper-bound multiplier (left) and *h*(*x*) = *x*^2^ with no multiplier (Lin et al., 2016, right), and their intersection graph (middle). Isolated nodes are included and the layout is fixed across plots and optimized for graph (a). In (b) the red nodes are hub nodes shared by both graphs, the green ones are hub nodes in graph (a) only, and the magenta ones are hub nodes in graph (c) only.

We compare our graph to the one in Lin et al. (2016), which corresponds to *h*(*x*) = *x*^2^ with no multiplier. Shown in Figure 11 are the estimated graphs, with their intersection in the middle. To improve visualization, isolated nodes are removed and the layouts are optimized for each plot. Red-colored points are the “hub nodes”, namely nodes with degree at least 10. In Figure 12, we plot the same graphs in a fixed layout optimized for the graph corresponding to *h* = min(*x*, 3), and include the isolated nodes.

Out of 333 edges, the two estimated graphs share 117 edges in common. Assuming that edges are placed at random between nodes and the two graphs are independent, the distribution of the number *R* of common edges follow a hypergeometric distribution, so $P(R=r)=\frac{\left(\begin{array}{c} m\\ r \end{array}\right)\left(\begin{array}{c} m(m-1)∕2-m\\ m-r \end{array}\right)}{\left(\begin{array}{c} m(m-1)∕2\\ m \end{array}\right)}$. For *m* = 333 the probability of at least 117 common edges is essentially zero. The large number of shared edges between the two methods can be explained by the fact that they both minimize the same underlying score-matching loss.

The graph using *h*(*x*) = min(*x*, 3) has much more isolated nodes (204) than the other (108), and has a slightly smaller max degree (16 versus 19). Table 3 provides another way of comparing between the two graphs by listing the genes with the highest node degrees.

In Table 3 we list the top ten genes in terms of node degree for both estimated graphs. Due to ties, 13 genes are listed for *h*(*x*) = min(*x*, 3) and 11 for Lin et al. (2016). As noted in Lin et al. (2016), genes with high node-degrees are known to be important in biological networks (Carter et al., 2004; Jeong et al., 2001; Han et al., 2004). Among these top genes, six are common in both graphs, and are discussed in Lin et al. (2016). We next elaborate on the evidence supporting the first four of the newly discovered genes.

- MMP2 (Matrix metalloproteinase 2): According to Trudel et al. (2003), increased MMP-2 expression is an independent predictor of decreased prostate cancer disease-free survival. Morgia et al. (2005) state that activity of MMP-2 can be useful in diagnosis, therapy, and assessment of malignant progression in prostate cancer.
- GLI2 (GLI family zinc finger 2): GLI2 is a primary mediator of the hedgehog signaling pathway, which has been reported in prostate cancer, and plays a critical role in the malignant phenotype of prostate cancer cells (Thiyagarajan et al., 2007). Its increased level of expression is also related to AI prostate cancer, and may be a therapeutic target in castrate-resistant prostate cancer (Narita et al., 2008).
- LAMA4 (Laminin subunit alpha 4): LAMA4 is consistently upregulated in benign prostatic hyperplasia when compared to normal prostate tissues (Luo et al., 2002).
- RASSF5 (RAS association domain family member 5): The combination of RASSF5 along with four other DNA methylation markers can effectively differentiate between benign prostate biopsy cores from non-cancer patients and cancer cores, and can be used to identify patients at risk without repeat biopsies (Brikun et al., 2014).

We note that the two methods indeed use different estimators (different *h* functions and multipliers), and it is thus not surprising to see that some of the top genes by one method are not among those for the other. In particular, CCNE2, BRCA2, SKP2 and STAT5B, while previously reported as newly discovered in Lin et al. (2016), are dropped by our new analysis. Testing and inference (potentially using bootstrapping) is an important problem but is beyond the scope of this paper.

## 8. Discussion

In this paper, we proposed a generalization of the score matching estimator of Hyvärinen (2007), based on scaling the log-gradients to be matched with a suitably chosen function ***h***. The generalization retains the advantages of Hyvärinen’s method: Estimates can be computed without knowledge of normalizing constants, and for canonical parameters of exponential families, the estimation loss is a quadratic function.

For high-dimensional exponential family graphical models, following Lin et al. (2016), we add an *ℓ*_1_ penalty to regularize the generalized score matching loss. One practical issue that is overlooked in Lin et al. (2016) is the fact that the score matching loss can be unbounded below for a small tuning parameter, when the dimension *m* exceeds the sample size *n*. We fix this issue by amplifying the diagonal entries in the quadratic matrix in the definition of the generalized score matching loss by a factor/multiplier, and we give an upper bound on that multiplier that guarantees consistency.

As examples we consider *pairwise interaction power models* on the non-negative orthant $R_{+}^{m}$. Specifically, the considered models are exponential families in which the log density is the sum of pairwise interactions between entries in of powers ***x***^*a*^ plus linearly weighted effects ***x***^*b*^, or log(***x***) when *b* = 0. Our main interest is in the matrix of interaction parameters whose support determines the distributions’ conditional independence graph. The considered framework covers truncated normal distributions (*a* = *b* = 1), exponential square root graphical models (*a* = *b* = 1/2) from Inouye et al. (2016), as well as a class of multivariate gamma distributions (*a* = 1/2, *b* = 0).

In the case of multivariate truncated normal distributions, where the conditional independence graph is given by the underlying Gaussian inverse covariance matrix, the sample size required for the consistency of our method using bounded ***h*** is Ω(*d*^2^ log *m*), where *d* is the degree of the graph. This matches the rates for Gaussian graphical models in Ravikumar et al. (2011) and Lin et al. (2016). In contrast, the sample complexity for truncated Gaussian models given in Lin et al. (2016) is Ω(*d*^2^ log^8^ *m*).

For the considered class of pairwise interaction models, we recommend using the function ***h*** with coordinates *h_j_*(*x*) = min (*x*^2–*a*^, *c*) for some moderately large *c*, or simply *h_j_*(*x*) = *x*^2–*a*^. While this choice is effective, it would be an interesting problem for future work to develop a method that adaptively chooses an optimized function ***h*** from data.

## Figures and Tables

**Figure 13:**
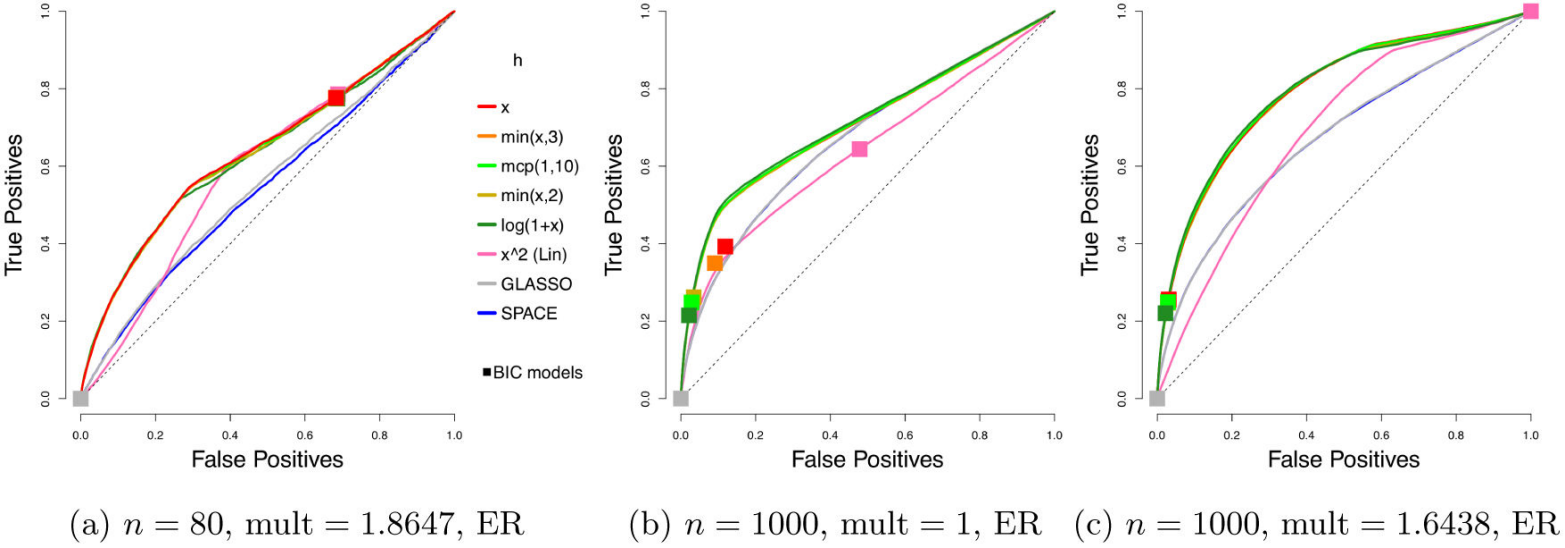
Average ROC curves of our *centered* estimator for *m* = 100 variables and two sample sizes *n* under various choices of *h*, compared to SPACE and GLASSO, for the *truncated centered GGM* case. Squares indicate average true positive rate (TPR) and false positive rate (FPR) of models picked by eBIC with refitting for the estimator in the same color.

**Figure 14:**
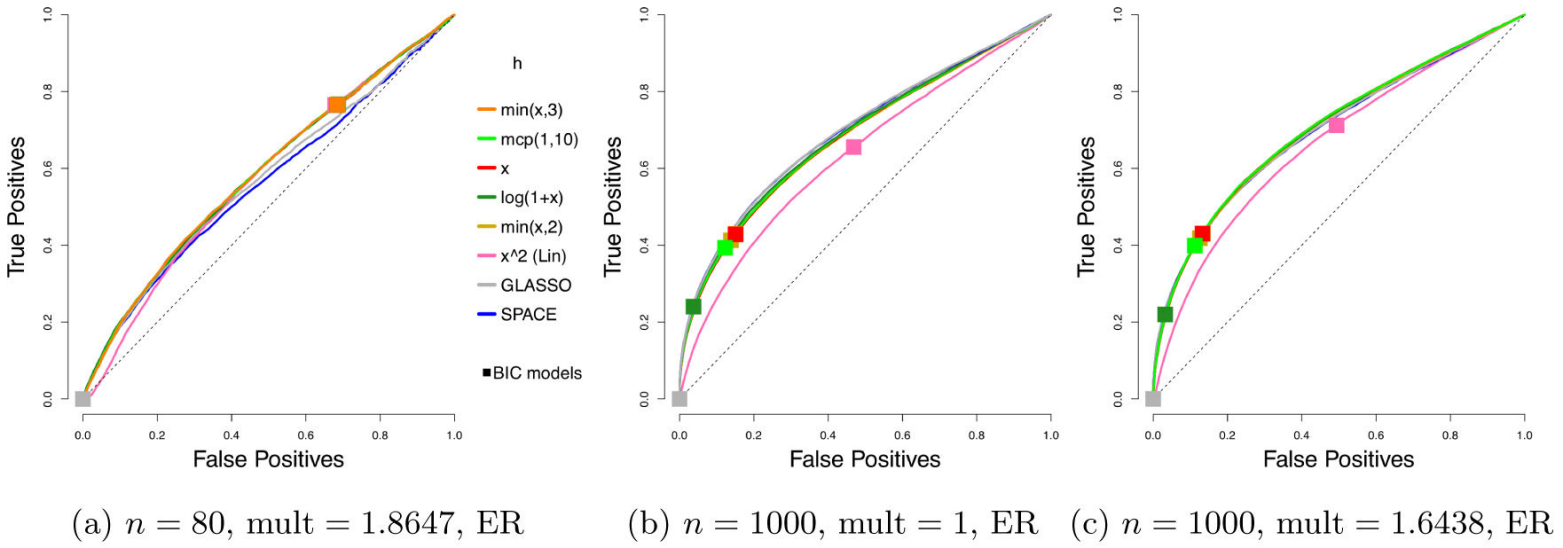
Average for the truncated non-centered GGM case. *n* = 80 or 1000, *m* = 100.

**Figure 15:**
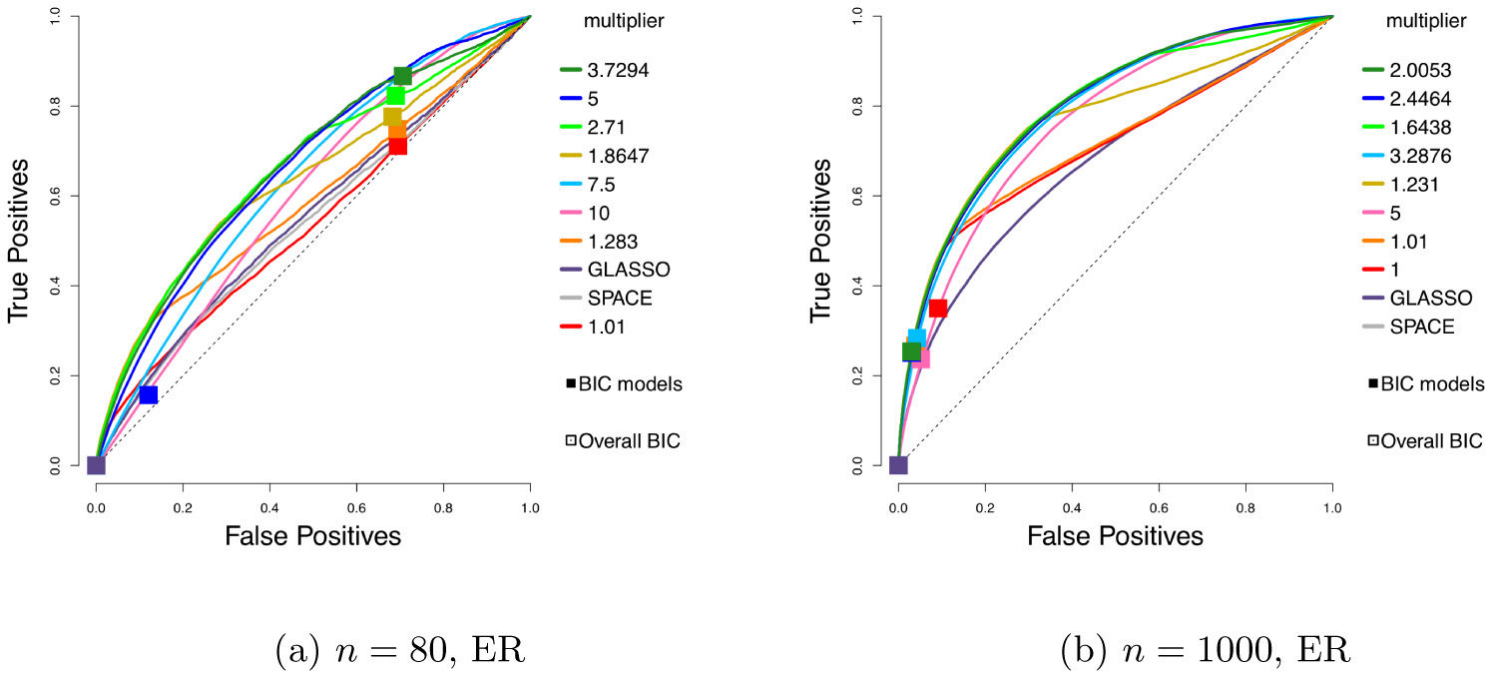
Performance of min(*x*, 3) for truncated centered GGMs with different multipliers, compared to GLASSO and SPACE, in the centered setting, *n* = 80 or 1000.

**Figure 16:**
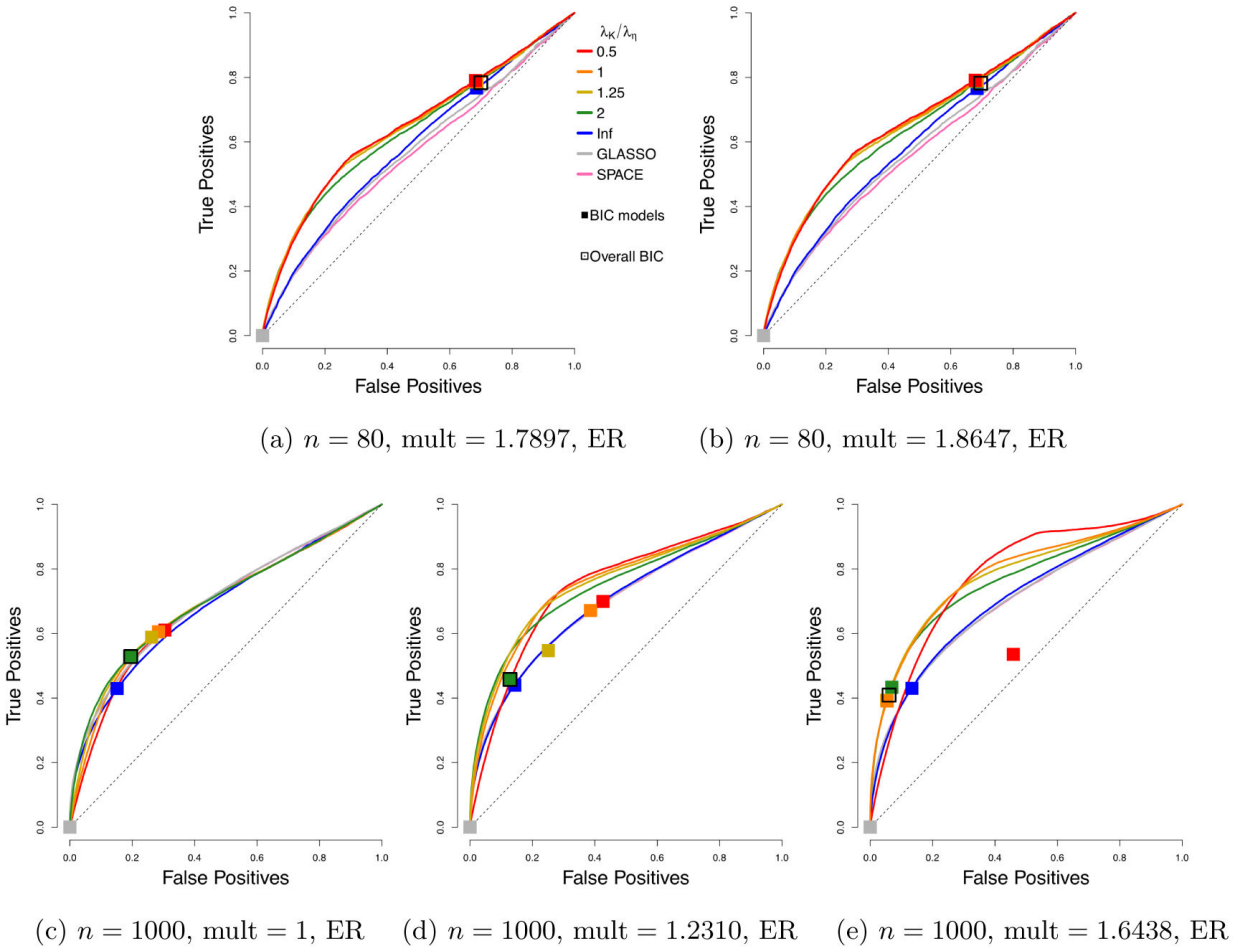
Performance of the non-centered estimator with *h*(*x*) = min(*x*, 3). Each curve corresponds to a different choice of *λ*_**K**_/*λ*_***η***_. Squares indicate models picked by eBIC with refit. The square with black outline has the highest eBIC among all models (combinations of *λ*_**K**_, *λ*_***η***_). The multipliers correspond to medium or high for *n* = 80, and low, medium and high for *n* = 1000, respectively.

**Figure 17:**
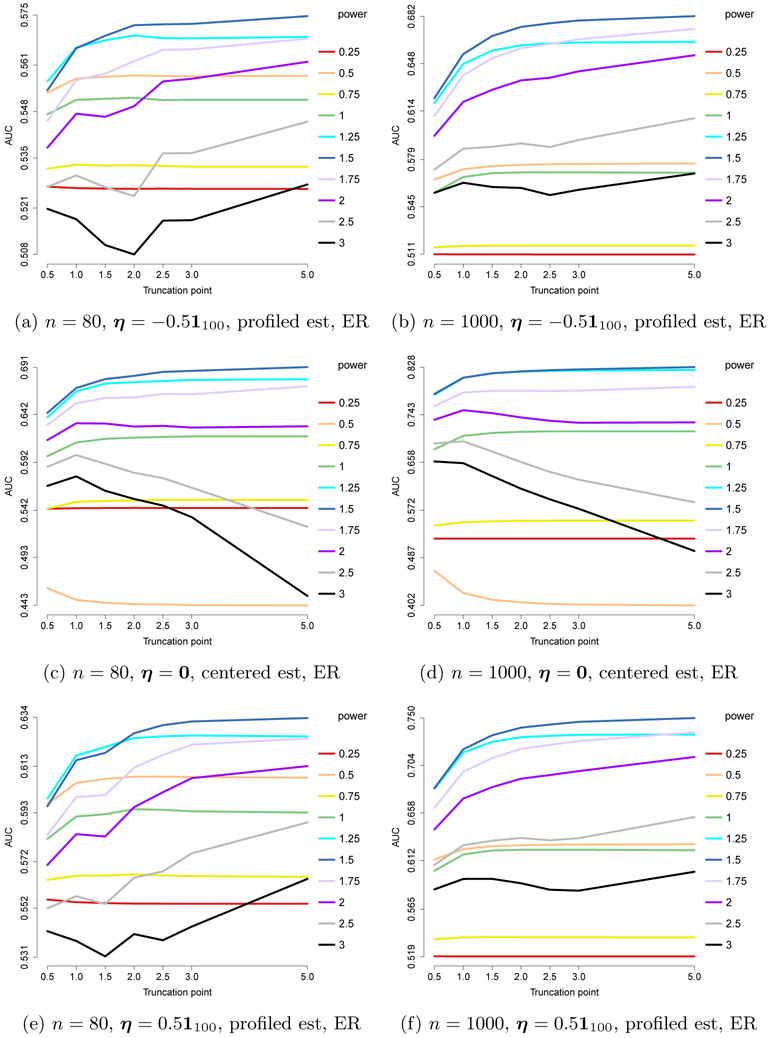
AUCs for edge recovery using generalized score matching for the exponential models. Each curve represents a different choice of power *p* in *h*(*x*) = min(*x^p^*, *c*), and the *x* axis marks the truncation point *c*. Colors are sorted by *p*.

**Figure 18:**
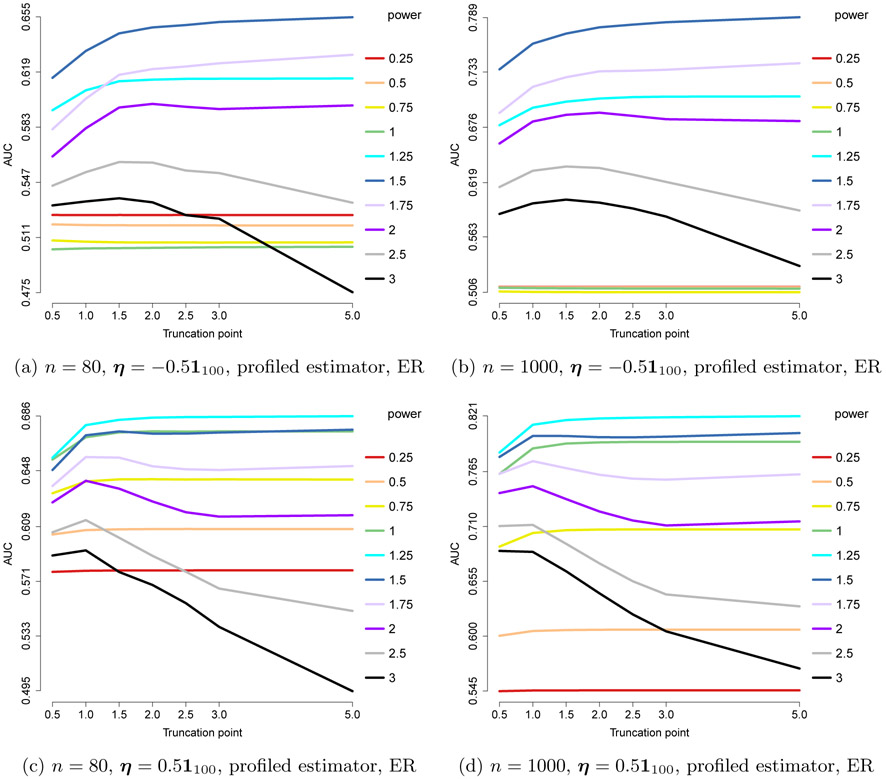
AUCs for edge recovery using generalized score matching for the gamma models. Each curve represents a different choice of power *p* in *h*(*x*) = min(*x^p^*, *c*), and the *x* axis marks the truncation point *c*. Colors are sorted by *p*.

**Figure 19:**
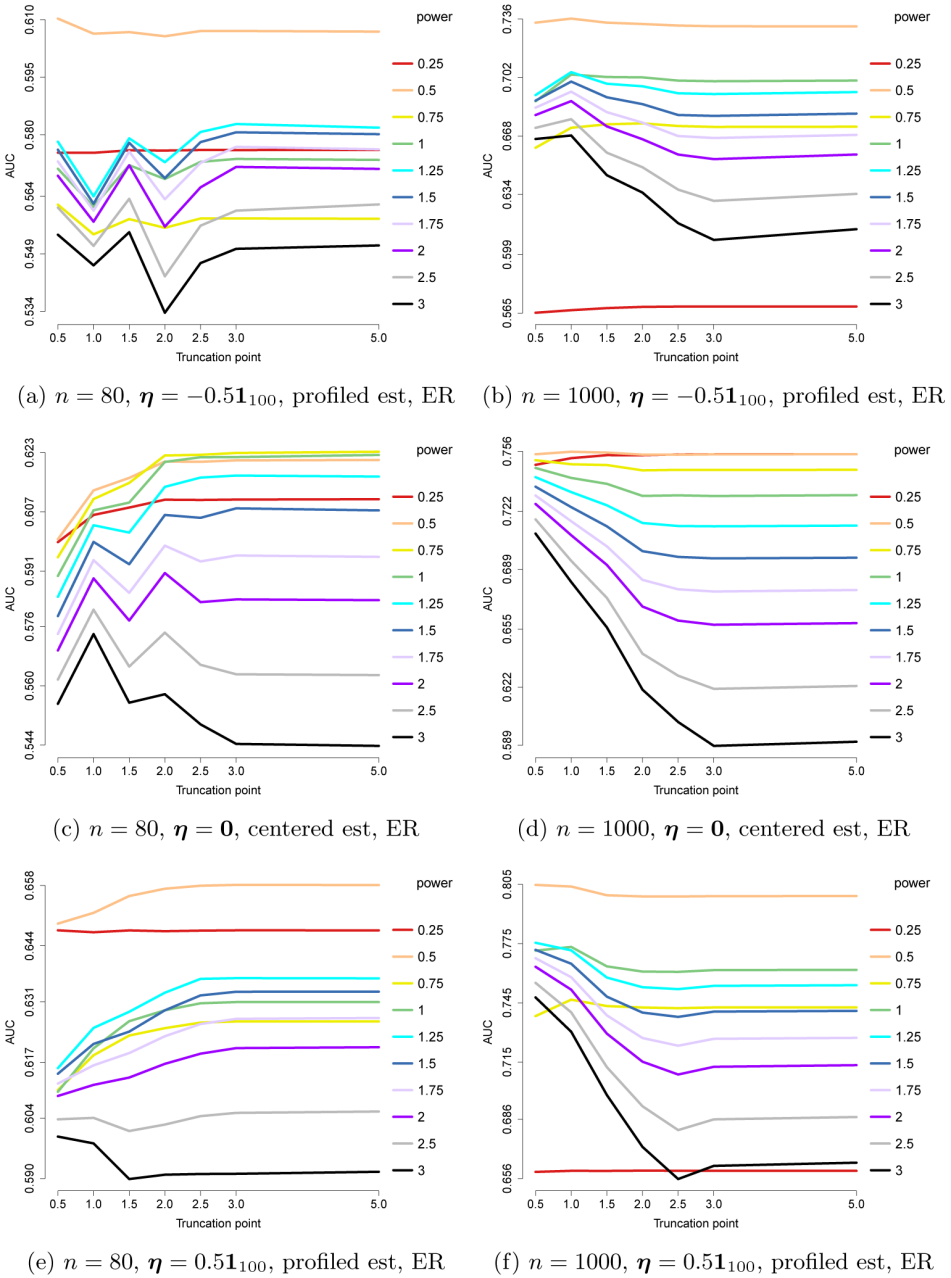
AUCs for edge recovery using generalized score matching for *a* = 3/2, *b* = 1/2. Each curve represents a different choice of power *p* in *h*(*x*) = min(*x^p^*, *c*), and the *x* axis marks the truncation point *c*. Colors are sorted by *p*.

**Figure 20:**
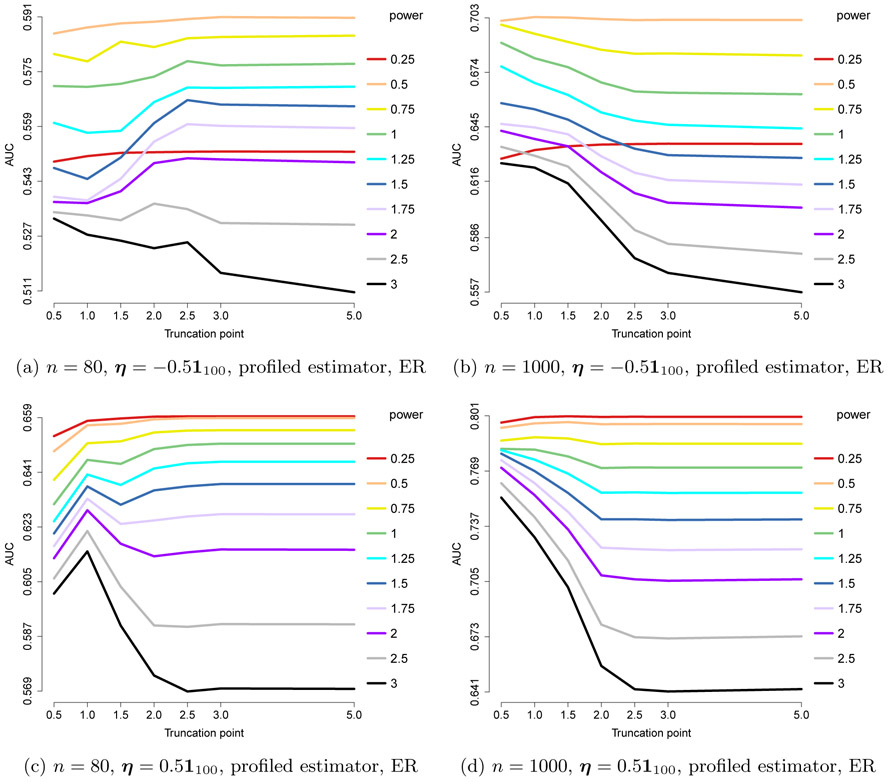
AUCs for edge recovery using generalized score matching for *a* = 3/2, *b* = 0. Each curve represents a different choice of power *p* in *h*(*x*) = min(*x^p^*, *c*), and the *x* axis marks the truncation point *c*. Colors are sorted by *p*.

**Table 1:** Mean and standard deviation of areas under the ROC curves (AUC) using different estimators in the centered setting, with *n* = 80 and multiplier 1.8647, or *n* = 1000 and multiplier 1 and 1.6438. Methods include our estimator with different choices of *h*, GLASSO, SPACE, neighborhood selection (NS), and Space JAM (SJ).

Centered, *n* = 80, multiplier 1.8647					
min(log(1 + *x*), *c*)			min(*x*, *c*)		
*c*	Mean	sd	*c*	Mean	sd
∞	0.694	0.033	∞	0.702	0.033
2	0.694	0.033	3	0.702	0.031
1	0.692	0.033	2	0.698	0.033
0.5	0.664	0.038	1	0.686	0.030
MCP(1, *c*)			SCAD(1, *c*)		
*c*	Mean	sd	*c*	Mean	sd
10	0.701	0.032	10	0.702	0.031
5	0.700	0.032	5	0.701	0.032
1	0.672	0.036	2	0.696	0.033
*x*^1.5^: (0.683, 0.030)			*x*^2^: (0.630, 0.029)		
GLASSO (0.600,0.032)			SPACE: (0.587, 0.031)		
NS: (0.587,0.031)			SJ: (0.540,0.036)		
Centered, *n* = 1000, multiplier 1					
min(log(1 + *x*), *c*)			min(*x*, *c*)		
*c*	Mean	sd	*c*	Mean	sd
2	0.826	0.015	2	0.820	0.014
∞	0.826	0.015	3	0.820	0.015
1	0.824	0.014	∞	0.819	0.015
0.5	0.804	0.015	1	0.817	0.014
MCP(1, *c*)			SCAD(1, *c*)		
*c*	Mean	sd	*c*	Mean	sd
5	0.824	0.015	2	0.823	0.014
10	0.822	0.015	5	0.822	0.015
1	0.810	0.015	10	0.821	0.015
*x*^1.5^: (0.782,0.014)			*x*^2^: (0.732,0.015)		
SPACE: (0.780,0.015)			NS: (0.779,0.015)		
GLASSO (0.764,0.014)			SJ: (0.703,0.015)		
Centered, *n* = 1000, multiplier 1.6438					
min(log(1 + *x*), *c*)			min(*x*, *c*)		
*c*	Mean	sd	*c*	Mean	sd
∞	0.857	0.011	3	0.855	0.011
2	0.857	0.011	∞	0.855	0.011
1	0.855	0.011	2	0.854	0.011
0.5	0.833	0.012	1	0.847	0.011
MCP(1, *c*)			SCAD(1, *c*)		
*c*	Mean	sd	*c*	Mean	sd
5	0.857	0.011	5	0.856	0.011
10	0.856	0.011	10	0.855	0.011
1	0.840	0.012	2	0.855	0.011
*x*^1.5^: (0.812,0.011)			*x*^2^: (0.736,0.011)		
SPACE: (0.780,0.015)			NS: (0.779,0.015)		
GLASSO (0.764,0.014)			SJ: (0.703,0.015)		

**Table 2:** Mean and standard deviation of AUC using different profiled estimators in the non-centered setting, with *n* = 80 and multiplier 1.8647, or *n* = 1000 and multipliers 1 and 1.6438. Methods include our estimator with different choices of *h*, GLASSO, SPACE, neighborhood selection (NS), and Space JAM (SJ).

Non-centered profiled, *n* = 80, multiplier 1.8647					
min(log(1 + *x*), *c*)			min(*x*, *c*)		
*c*	Mean	sd	*c*	Mean	sd
∞	0.632	0.032	∞	0.634	0.032
2	0.632	0.032	3	0.634	0.032
1	0.631	0.032	2	0.632	0.032
0.5	0.619	0.033	1	0.628	0.032
MCP(1, *c*)			SCAD(1, *c*)		
*c*	Mean	sd	*c*	Mean	sd
10	0.634	0.032	5	0.634	0.032
5	0.634	0.032	10	0.634	0.032
1	0.622	0.032	2	0.634	0.032
*x*^1.5^: (0.623,0.031)			*x*^2^: (0.607,0.030)		
GLASSO: (0.614,0.029)			NS: (0.604,0.028)		
SPACE: (0.602,0.029)			SJ: (0.561,0.036)		
Non-centered profiled, *n* = 1000, multiplier 1					
min(log(1 + *x*), *c*)			min(*x*, *c*)		
*c*	Mean	sd	*c*	Mean	sd
∞	0.783	0.020	2	0.779	0.020
2	0.783	0.020	∞	0.779	0.020
1	0.782	0.020	3	0.779	0.020
0.5	0.767	0.021	0.5	0.758	0.020
MCP(1, *c*)			SCAD(1, *c*)		
*c*	Mean	sd	*c*	Mean	sd
5	0.782	0.020	2	0.780	0.020
10	0.780	0.020	5	0.780	0.020
1	0.771	0.021	10	0.779	0.020
*x*^1.5^: (0.751,0.019)			*x*^2^: (0.713,0.018)		
SPACE: (0.786,0.020)			NS: (0.785,0.02)		
GLASSO (0.770,0.019)			SJ: (0.720,0.019)		
Non-centered profiled, *n* = 1000, multiplier 1.6438					
min(log(1 + *x*), *c*)			min(*x*, *c*)		
*c*	Mean	sd	*c*	Mean	sd
∞	0.764	0.018	∞	0.766	0.019
2	0.764	0.018	3	0.765	0.019
1	0.762	0.018	2	0.764	0.018
0.5	0.738	0.018	1	0.753	0.018
MCP(1, *c*)			SCAD(1, *c*)		
*c*	Mean	sd	*c*	Mean	sd
10	0.766	0.019	10	0.766	0.019
5	0.766	0.019	5	0.766	0.019
1	0.745	0.018	2	0.763	0.018
*x*^1.5^: (0.748,0.018)			*x*^2^: (0.718,0.017)		
SPACE: (0.786,0.020)			NS: (0.785,0.020)		
GLASSO (0.770,0.019)			SJ: (0.720,0.019)		

**Table 3:** List of genes with the highest node degrees in each estimated graph.

min(*x*, 3) with multiplier 1.63	Lin et al.
**LAMB3 (16)**	CCNE2 (19)
**PIK3CG (16)**	**PIK3CG (16)**
MMP2 (15)	BRCA2 (13)
GLI2 (13)	**BIRC5 (12)**
LAMA4 (13)	**LAMB3 (10)**
**PDGFRB (13)**	**PIK3CD (10)**
**PIK3CD (13)**	SKP2 (10)
RASSF5 (13)	HRAS (9)
**BIRC5 (12)**	STAT5B (9)
FLT3 (12)	**GSTP1(8)**
**GSTP1 (12)**	**PDGFRB (8)**
LAMA2 (12)	
RAC2 (12)	

**Table 4:** Mean and standard deviation of areas under the ROC curves (AUC) using different estimators in the centered setting, with *n* = 80 and multiplier 1.8647, or *n* = 1000 and multipliers 1 and 1.6438. Methods include our estimator with different choices of *h*, GLASSO, SPACE, neighborhood selection (NS), and Space JAM (SJ).

Centered, *n* = 80, multiplier 1.8647, ER					
min(log(1 + *x*), *c*)			min(*x*, *c*)		
*c*	Mean	sd	*c*	Mean	sd
∞	0.632	0.036	∞	0.638	0.035
2	0.632	0.036	3	0.638	0.035
1	0.630	0.035	2	0.635	0.035
0.5	0.613	0.033	1	0.623	0.033
MCP(1, *c*)			SCAD(1, *c*)		
*c*	Mean	sd	*c*	Mean	sd
10	0.637	0.035	10	0.638	0.035
5	0.636	0.036	5	0.637	0.035
1	0.617	0.033	2	0.632	0.035
*x*^1.5^: (0.627, 0.032)			*x*^2^: (0.595,0.028)		
GLASSO (0.553,0.029)			SPACE: (0.544, 0.026)		
NS: (0.543,0.028)			SJ: (0.519,0.028)		
Centered, *n* = 1000, multiplier 1, ER					
min(log(1 + *x*), *c*)			min(*x*, *c*)		
*c*	Mean	sd	*c*	Mean	sd
∞	0.716	0.016	2	0.710	0.016
2	0.716	0.016	3	0.710	0.016
1	0.715	0.016	1	0.710	0.017
0.5	0.694	0.017	∞	0.709	0.016
MCP(1, *c*)			SCAD(1, *c*)		
*c*	Mean	sd	*c*	Mean	sd
5	0.714	0.016	2	0.713	0.016
10	0.711	0.016	5	0.711	0.016
1	0.707	0.017	10	0.710	0.016
*x*^1.5^: (0.678,0.016)			*x*^2^: (0.64,0.017)		
GLASSO: (0.675,0.016)			SPACE: (0.675,0.016)		
NS: (0.675,0.016)			SJ: (0.624,0.017)		
Centered, *n* = 1000, multiplier 1.6438, ER					
min(log(1 + *x*), *c*)			min(*x*, *c*)		
*c*	Mean	sd	*c*	Mean	sd
∞	0.796	0.014	∞	0.795	0.014
2	0.796	0.014	3	0.794	0.014
1	0.794	0.014	2	0.792	0.014
0.5	0.772	0.015	1	0.784	0.015
MCP(1, *c*)			SCAD(1, *c*)		
*c*	Mean	sd	*c*	Mean	sd
5	0.796	0.014	5	0.795	0.014
10	0.796	0.014	10	0.795	0.014
1	0.778	0.015	2	0.793	0.014
*x*^1.5^: (0.757,0.015)			*x*^2^: (0.693,0.016)		
GLASSO: (0.675,0.016)			SPACE: (0.675,0.016)		
NS: (0.675,0.016)			SJ: (0.624,0.017)		

**Table 5:** Mean and standard deviation of AUC using different profiled estimators in the non-centered setting, with *n* = 80 and multiplier 1.8647, or *n* = 1000 and multipliers 1 and 1.6438. Methods as for Table 4.

Non-centered profiled, *n* = 80, multiplier 1.8647, ER					
min(log(1 + *x*), *c*)			min(*x*, *c*)		
*c*	Mean	sd	*c*	Mean	sd
1	0.588	0.034	3	0.588	0.033
∞	0.588	0.034	∞	0.588	0.033
2	0.588	0.034	2	0.588	0.033
0.5	0.576	0.033	1	0.583	0.033
MCP(1, *c*)			SCAD(1, *c*)		
*c*	Mean	sd	*c*	Mean	sd
5	0.588	0.033	5	0.588	0.033
10	0.588	0.033	10	0.588	0.033
1	0.581	0.033	2	0.587	0.033
*x*^1.5^: (0.582,0.028)			*x*^2^: (0.576,0.028)		
GLASSO: (0.572,0.033)			SPACE: (0.562,0.031)		
NS: (0.560,0.032)			SJ: (0.535,0.027)		
Non-centered profiled, *n* = 1000, multiplier 1, ER					
min(log(1 + *x*), *c*)			min(*x*, *c*)		
*c*	Mean	sd	*c*	Mean	sd
2	0.692	0.022	1	0.687	0.022
∞	0.692	0.022	∞	0.686	0.022
1	0.691	0.022	3	0.685	0.022
0.5	0.684	0.02	2	0.685	0.022
MCP(1, *c*)			SCAD(1, *c*)		
*c*	Mean	sd	*c*	Mean	sd
5	0.689	0.022	2	0.687	0.022
1	0.689	0.020	5	0.687	0.022
10	0.687	0.022	10	0.686	0.022
*x*^1.5^: (0.663,0.020)			*x*^2^: (0.638,0.019)		
GLASSO (0.700,0.022)			SPACE: (0.699,0.022)		
NS: (0.699,0.022)			SJ: (0.655,0.021)		
Non-centered profiled, *n* = 1000, multiplier 1.6438, ER					
min(log(1 + *x*), *c*)			min(*x*, *c*)		
*c*	Mean	sd	*c*	Mean	sd
2	0.705	0.021	∞	0.705	0.022
∞	0.705	0.021	3	0.705	0.021
1	0.703	0.021	2	0.702	0.022
0.5	0.683	0.019	1	0.695	0.021
MCP(1, *c*)			SCAD(1, *c*)		
*c*	Mean	sd	*c*	Mean	sd
5	0.706	0.021	10	0.705	0.022
10	0.706	0.022	5	0.705	0.022
1	0.690	0.019	2	0.703	0.022
*x*^1.5^: (0.689,0.021)			*x*^2^: (0.664,0.019)		
GLASSO (0.700,0.022)			SPACE: (0.699,0.022)		
NS: (0.699,0.022)			SJ: (0.655,0.021)		

## Appendix

### Appendix A. Proofs

#### A.1. Proof of Theorem 3

The following integration by parts lemma is used in the proof of Theorem 3.

**Lemma 19** *Let f, g* : $R_{+}\to R$ *be functions that are absolutely continuous in every bounded sub-interval of* $R_{+}$. *Then*

$$\underset{x↗+\infty }{\text{lim}}f(x)g(x)-\underset{x↘0^{+}}{\text{lim}}f(x)g(x)=\int_{0}^{\infty }f(x)\frac{dg(x)}{dx}\;dx+\int_{0}^{\infty }g(x)\frac{df(x)}{dx}\;dx.$$

**Proof** This is an analog of Lemma 4 from Hyvärinen (2005) that can be proved by integrating the partial derivatives, and follows from the fundamental theorem of calculus for absolutely continuous functions and the product rule. In particular, we work on integrals in bounded [0, *c*], where the product of two absolutely continuous functions in a bounded interval is again absolutely continuous, and the result is then obtained by letting *c* ↗ +∞. ■

**Proof** [Proof of Theorem 3] Recall the following assumptions from Section 2.2:

(A1)
p0(x)hj(xj)∂jlogp(x)∣xj↘0+xj↗+∞=0,∀x−j∈R+m−1,∀p∈P+;∣

(A2)
$$E_{p_{0}}\|\nabla \;\log\;p(X)\;\circ \;h^{1∕2}(X){\|}_{2}^{2}<+\infty ,\;E_{p_{0}}\|(\nabla \;\log\;p(X)\;\circ \;h(X){)}^{\prime }{\|}_{1}<+\infty ,\;\forall p\in P_{+}.$$

Without explicitly writing the domains $R_{+}$ or $R_{+}^{m}$ in all integrals, by (6) we have

Jh(p)=12∫p0(x)[‖∇logp(x)∘h1∕2(x)‖22]−[2(∇logp(x)∘h1∕2(x))⊤(∇logp0(x)∘h1∕2(x))+‖∇logp0(x)∘h1∕2(x)‖22]dx=12∫p0(x)∑j=1mhj(xj)(∂logp(x)∂xj)2dx︸≡A+12∫p0(x)∑j=1mhj(xj)(∂logp0(x)∂xj)2dx︸≡C−∫p0(x)∑j=1mhj(xj)∂logp(x)∂xj∂logp0(x)∂xjdx︸≡B,

where *A* will simply appear in the final display as is, *C* is a constant as it only involves the true pdf *p*_0_, and we wish to simplify *B* by integration by parts. We can split the integral into these three parts since *A* and *C* are assumed finite in the first part of (A2), and the integrand in *B* is integrable since ∣2*ab*∣ ≤ *a*^2^ + *b*^2^. Thus, by linearity and Fubini’s theorem, we can write

$$B=-\sum_{j=1}^{m}\int p_{0}(x)h_{j}(x_{j})\frac{\partial \;\log\;p(x)}{\partial x_{j}}\frac{\partial \;\log\;p_{0}(x)}{\partial x_{j}}\;dx\;\;=-\sum_{j=1}^{m}\int \left[\int p_{0}(x)h_{j}(x_{j})\frac{\partial \;\log\;p(x)}{\partial x_{j}}\frac{\partial \;\log\;p_{0}(x)}{\partial x_{j}}\;dx_{j}\right]dx_{-j}.$$

By the fact that $\frac{\partial \;\log\;p_{0}(x)}{\partial x_{j}}=\frac{1}{p_{0}(x)}\frac{\partial p_{0}(x)}{\partial x_{j}}$, this can be simplified to

$$B=-\sum_{j=1}^{m}\int \left[\int \frac{\partial p_{0}(x)}{\partial x_{j}}h_{j}(x_{j})\frac{\partial \;\log\;p(x)}{\partial x_{j}}\;dx_{j}\right]dx_{-j}.$$

But, we assume *p*_0_ and *p* are twice continuously differentiable, for every *j* = 1,…,*m* and fixed $x_{-j}\in R_{+}^{m-1}$. Hence, in every bounded sub-interval of $R_{+}$, *p*_0_(***x***_−*j*_; *x_j_*) is an absolutely continuous function of *x_j_*, *∂_j_* log *p*(*x*_−*j*_, *x_j_*) = *∂_j_p*(*x*_−*j*_, *x_j_*)/*p*(***x***_−*j*_, *x_j_*) is a continuously differentiable (and hence absolutely continuous) function of *x_j_* by the quotient rule. Thus *h_j_*(*x_j_*)*∂_j_* log *p*(***x***_−*j*_; *x_j_*) is also absolutely continuous by the absolute continuity assumption on *h_j_*. Then, by Lemma 19, where we take *f* ≡ *p*_0_(***x***_−*j*_; *x_j_*) and *g* ≡ *h_j_*(*x_j_*)_∂_j__ log *p*(***x***_−*j*_; *x_j_*) as functions of *x_j_*, followed by assumption (A1),

B=−∑j=1m∫[lima↗+∞,b↘0+[p0(x−j;a)hj(a)∂jlogp(x−j,a)−p0(x−j;b)hj(b)∂jlogp(x−j,b)]]−[∫p0(x)∂(hj(xj)∂jlogp(x))∂xjdxj]dx−j=∑j=1m∫[∫p0(x)∂(hj(xj)∂jlogp(x))∂xjdxj]dx−j.

Justified by the second half of (A2), by Fubini-Tonelli and linearity again

$$B=\sum_{j=1}^{m}\int p_{0}(x)\frac{\partial (h_{j}(x_{j})\partial_{j}\;\log\;p(x))}{\partial x_{j}}\;dx,\;\;=\sum_{j=1}^{m}\int h_{j}^{\prime }(x_{j})\frac{\partial \;\log\;p(x)}{\partial x_{j}}p_{0}(x)\;dx+\sum_{j=1}^{m}\int h_{j}(x_{j})\frac{\partial^{2}\log\;p(x)}{\partial x_{j}^{2}}p_{0}(x)dx.$$

Thus

$$\;J_{h}(p)\;=B+A+C\;=\int_{R_{+}^{m}}p_{0}(x)\sum_{j=1}^{m}\left[h_{j}^{\prime }(x_{j})\frac{\partial \;\log\;p(x)}{\partial x_{j}}+h_{j}(x_{j})\frac{\partial^{2}\log\;p(x)}{\partial x_{j}^{2}}+\frac{1}{2}h_{j}(x_{j}){\left(\frac{\partial \;\log\;p(x)}{\partial x_{j}}\right)}^{2}\right]dx+C,$$

where *C* is a constant that does not depend on *p*. ■

#### A.2. Proof of Theorems and Examples in Section 3

**Proof** [Proof of Theorem 5] For exponential families and under the assumptions, the empirical loss ${\hat{J}}_{h}(p_{\theta })$ in (8) becomes (up to an additive constant)

J^h(pθ)=1n∑i=1n∑j=1m[hj′(Xj(i))∂logpθ(X(i))∂Xj(i)+hj(Xj(i))∂2logpθ(X(i))∂(Xj(i))2][+12hj(Xj(i))(∂logpθ(X(i))∂Xj(i))2]=1n∑i=1n∑j=1m[hj′(Xj(i))(θ⊤tj′(X(i))+bj′(X(i)))+hj(Xj(i))(θ⊤tj″(X(i))+bj″(X(i)))][+12hj(Xj(i))(θ⊤tj′(X(i))+bj′(X(i)))2]=1n{12θ⊤[∑i=1n∑j=1mhj(Xj(i))tj′(X(i))tj′(X(i))⊤]θ+}{[∑i=1n∑j=1mhj(Xj(i))bj′(X(i))tj′(X(i))+hj(Xj(i))tj″(X(i))+hj′(Xj(i))tj′(X(i))]⊤θ}+const,

which is quadratic in ***θ***. Let

(34)
$$\Gamma (x)=\frac{1}{n}\sum_{i=1}^{n}\sum_{j=1}^{m}h_{j}(X_{j}^{\left(i\right)})t_{j}^{\prime }(X^{\left(i\right)})t_{j}^{\prime }(X^{\left(i\right)}{)}^{⊤},$$

(35)
$$g(x)=-\frac{1}{n}\sum_{i=1}^{n}\sum_{j=1}^{m}\left[h_{j}(X_{j}^{\left(i\right)})b_{j}^{\prime }(X^{\left(i\right)})t_{j}^{\prime }(X^{\left(i\right)})+h_{j}(X_{j}^{\left(i\right)})t_{j}^{\prime \prime }(X^{\left(i\right)})+h_{j}^{\prime }(X_{j}^{\left(i\right)})t_{j}^{\prime }(X_{i})\right].$$

Then we can write ${\hat{J}}_{h}(p_{\theta })=\frac{1}{2}\theta^{⊤}\Gamma (x)\theta -g(x{)}^{⊤}\theta +\text{const}$. ■

**Proof** [Proof of Theorem 6] By Theorem 5, ${\hat{J}}_{h}(p_{\theta })=\frac{1}{2}\theta^{⊤}\Gamma \theta -g^{⊤}\theta +\text{const}$. The minimizer of ${\hat{J}}_{h}(p_{\theta })$ is thus available in the unique closed form $\hat{\theta }\equiv \Gamma (x{)}^{-1}g(x)$ as long as **Γ** is invertible (C1). Since **Γ** and ***g*** are sample averages, the weak law of large numbers yields that $\Gamma \to_{p}E_{p_{0}}\Gamma \equiv \Gamma_{0}$ and $g\to_{p}E_{p_{0}}g\equiv g_{0}$, where existence of **Γ**_0_ and ***g***_0_ is assumed in (C2). Since $J_{h}(p_{\theta })=E[{\hat{J}}_{h}(p_{\theta })]=E[\frac{1}{2}\theta^{⊤}\Gamma (x)\theta -g(x{)}^{⊤}\theta ]=\frac{1}{2}\theta^{⊤}\Gamma_{0}\theta -g_{0}\theta$ and we know ***θ***_0_ minimizes *J*_***h***_(*p_**θ**_*) by definition, by the first-order condition we must have **Γ**_0_***θ***_0_ = ***g***_0_. Then by the Lindeberg-Lévy central limit theorem,

$$\sqrt{n}(g(x)-\Gamma (x)\theta_{0})\to_{d}N_{m}(0,\Sigma_{0}),$$

where $\Sigma_{0}\equiv E_{p_{0}}[(\Gamma (x)\theta_{0}-g(x))(\Gamma (x)\theta_{0}-g(x){)}^{⊤}]$, as long as **Σ**_0_ exists (C2). Thus, by Slutsky’s theorem,

$$\sqrt{n}(\hat{\theta }-\theta_{0})\equiv \sqrt{n}(\Gamma (x{)}^{-1}(g(x)-\Gamma (x)\theta_{0}))\to_{d}N_{r}(0,\Gamma_{0}^{-1}\Sigma \Gamma_{0}^{-1}),$$

as long as **Γ**_0_ is invertible (C2).

For the second half of the theorem, (C2) $E_{p_{0}}\Gamma (x)<\infty$ and $E_{p_{0}}g(x)<\infty$ implies $E_{p_{0}}|\Gamma (x)|<\infty$ and $E_{p_{0}}|g(x)|<\infty$, so by strong law of large numbers (and a union bound on at most *k*^2^ null sets)

$$\Gamma (x)\to_{\text{a.s.}}\Gamma_{0},\;\;g(x)\to_{\text{a.s.}}g_{0}.$$

Then outside a null set,

$$\hat{\theta }\equiv \Gamma (x{)}^{-1}g(x)\to_{\text{a.s.}}\Gamma_{0}^{-1}g_{0}=\theta_{0}.$$

■

**Proof** [Proof for Example 3.1] We choose to estimate *θ* ≡ *μ*/*σ*^2^. Then by (11) and (12),

$${\hat{\mu }}_{h}=\sigma^{2}\hat{\theta }\equiv \sigma^{2}\Gamma (x{)}^{-1}g(x)\;\;=-\sigma^{2}{\left[\sum_{i=1}^{n}h(X_{i})t^{\prime }(X_{i}{)}^{2}\right]}^{-1}\left[\sum_{i=1}^{n}h(X_{i})b^{\prime }(X_{i})t^{\prime }(X_{i})+h(X_{i})t^{\prime \prime }(X_{i})+h^{\prime }(X_{i})t^{\prime }(X_{i})\right]\;\;=-\sigma^{2}{\left[\sum_{i=1}^{n}h(X_{i})\right]}^{-1}\left[\sum_{i=1}^{n}-h(X_{i})\frac{X_{i}}{\sigma^{2}}+h^{\prime }(X_{i})\right].$$

By Theorem 6,

$$\sqrt{n}({\hat{\mu }}_{h}-\mu_{0})\to_{d}N\left(0,\frac{\sigma^{4}E_{0}{\left[-h(X)\frac{X-\mu_{0}}{\sigma^{2}}+h^{\prime }(X)\right]}^{2}}{E_{0}^{2}[h(X)]}\right)\;\;∼N\left(0,\frac{E_{0}\;[-h(X)(X-\mu_{0})+\sigma^{2}h^{\prime }(X){]}^{2}}{E_{0}^{2}[h(X)]}\right).$$

By integration by parts, (suppressing the dependence of *p*_*μ*_0__ on *μ*_0_)

$$\;E_{0}[h(X)h^{\prime }(X)(X-\mu_{0})]\;=\int_{0}^{\infty }h^{\prime }(x)h(x)(x-\mu_{0})p(x)\;dx\;=\int_{0}^{\infty }h(x)(x-\mu_{0})p(x)\;dh(x)\;=h^{2}(x)(x-\mu_{0})p(x){|}_{0}^{\infty }-\int h(x)\;dh(x)(x-\mu_{0})p(x)\;=-\int h^{2}(x)p(x)\;dx-\int h(x)h^{\prime }(x)(x-\mu_{0})p(x)\;dx+\int h^{2}(x)\frac{(x-\mu_{0}{)}^{2}}{\sigma^{2}}p(x)\;dx,$$

where the last step follows from the assumptions $\underset{x↘0^{+}}{\text{lim}}h(x)=0$ and $\underset{x↗+\infty }{\text{lim}}h^{2}(x)(x-\mu_{0})p_{\mu_{0}}(x)=0$. So

(36)
$$E_{0}[h(X)h^{\prime }(X)(X-\mu_{0})]=\frac{E[h^{2}(X)((X-\mu_{0}{)}^{2}∕\sigma^{2}-1)]}{2}.$$

The asymptotic variance is thus

$$\;\frac{E_{0}{\left[-h(X)(X-\mu_{0})+\sigma^{2}h^{\prime }(X)\right]}^{2}}{E_{0}^{2}[h(X)]}\;=\frac{E_{0}\left[h^{2}(X)(X-\mu_{0}{)}^{2}-2\sigma^{2}h^{2}(X)((X-\mu_{0}{)}^{2}∕\sigma^{2}-1)\;∕2+\sigma^{4}h^{\prime^{2}}(X)\right]}{E_{0}^{2}[h(X)]}\;=\frac{E_{0}[\sigma^{2}h^{2}(X)+\sigma^{4}h^{\prime^{2}}(X)}{E_{0}^{2}[h(X)]}.$$

The Cramér-Rao lower bound follows from taking the second derivative of log *p*_*μ*_0__ with respect to *μ*_0_. ■

**Proof** [Proof for Example 3.2] We estimate *θ* ≡ 1/*σ*^2^. By (11) and (12),

$$\hat{\theta }\equiv \Gamma (x{)}^{-1}g(x)\;\;=-{\left[\sum_{i=1}^{n}h(X_{i})t^{\prime }(X_{i}{)}^{2}\right]}^{-1}\left[\sum_{i=1}^{n}h(X_{i})b^{\prime }(X_{i})t^{\prime }(X_{i})+h(X_{i})t^{\prime \prime }(X_{i})+h^{\prime }(X_{i})t^{\prime }(X_{i})\right]\;\;={\left[\sum_{i=1}^{n}h(X_{i})(X_{i}-\mu {)}^{2}\right]}^{-1}\left[\sum_{i=1}^{n}h(X_{i})+h^{\prime }(X_{i})(X_{i}-\mu )\right].$$

By Theorem 6, $\sqrt{n}(\hat{\theta }-\theta )\to_{d}N(0,\varsigma^{2})$, where

ς2≡E0[h(X)((X−μ)2∕σ02−1)−h′(X)(X−μ)]2E02[h(X)(X−μ)2]=1E02[h(X)(X−μ)2](E0[h2(X)(X−μ)4∕σ04−2h2(X)(X−μ)2∕σ02+h2(X))(+h′2(X)(X−μ)2−2h(X)h′(X)(X−μ)3∕σ02+2h(X)h′(X)(X−μ)).

By integration by parts, (suppressing the dependence of $p_{\sigma_{0}^{2}}$ on $\sigma_{0}^{2}$)

$$\;E_{0}[h(X)h^{\prime }(X)(X-\mu {)}^{3}]\;=\int_{0}^{\infty }h^{\prime }(x)h(x)(x-\mu {)}^{3}p(x)\;dx\;=\int_{0}^{\infty }h(x)(x-\mu {)}^{3}p(x)\;dh(x)\;=h^{2}(x)(x-\mu {)}^{3}p(x){|}_{0}^{\infty }-\int h(x)\;dh(x)(x-\mu {)}^{3}p(x)\;=-\int h(x)h^{\prime }(x)(x-\mu {)}^{3}p(x)\;dx-3\int h^{2}(x)(x-\mu {)}^{2}p(x)\;dx+\int h^{2}(x)\frac{(x-\mu {)}^{4}}{\sigma_{0}^{2}}p(x)\;dx,$$

where the last step follows from the assumptions $\underset{x↘0^{+}}{\text{lim}}h(x)=0$ and $\underset{x↗+\infty }{\text{lim}}h^{2}(x)(x-\mu {)}^{3}p_{\sigma_{0}^{2}}(x)=0$. Combining this with (36) we get

$$\sqrt{n}(\hat{\theta }-\theta )\to_{d}N(0,\varsigma^{2})∼N\left(0,\frac{2E_{0}[h^{2}(X)(X-\mu {)}^{2}∕\sigma_{0}^{2}]+E_{0}[h^{\prime^{2}}(X-\mu {)}^{2}]}{E_{0}^{2}[h(X)(X-\mu {)}^{2}]}\right),$$

and so by the delta method, for ${\hat{\sigma }}_{k}^{2}\equiv {\hat{\theta }}^{-1}$,

$$\sqrt{n}({\hat{\sigma }}_{h}^{2}-\sigma_{0}^{2})\to_{d}N\left(0,\frac{2\sigma_{0}^{6}E_{0}[h^{2}(X)(X-\mu {)}^{2}]+\sigma_{0}^{8}E[h^{\prime^{2}}(X-\mu {)}^{2}]}{E_{0}^{2}[h(X)(X-\mu {)}^{2}]}\right).$$

The Cramér-Rao lower bound follows from taking the second derivative of log $p_{\sigma_{0}^{2}}$ with respect to $\sigma_{0}^{2}$. ■

#### A.3. Proof of Theorems in Section 5

**Proof** [Proof of Theorem 9]

*Case b* ≠ 0: We use a strategy similar to that of Inouye et al. (2016). Let $V_{1}=\{v\;:\|v{\|}_{1}=1,v\in R_{+}^{m}\}$. Then by Fubini-Tonelli the normalizing constant is,

$$\;\int_{R_{+}^{m}}\text{exp}\left(\eta^{⊤}\frac{x^{b}-1_{m}}{b}-\frac{1}{2a}x^{a⊤}Kx^{a}\right)\;dx\;=\int_{V_{1}}\int_{0}^{\infty }\text{exp}\left(\eta^{⊤}\frac{z^{b}v^{b}-1_{m}}{b}-\frac{1}{2a}z^{2a}v^{a⊤}Kv^{a}\right)\;dz\;dv\;\propto \int_{V_{1}}\int_{0}^{\infty }\text{exp}\left(z^{b}(\eta^{⊤}v^{b})∕b-z^{2a}(v^{a⊤}Kv^{a})∕(2a)\right)\;dz\;dv.$$

Here $V_{1}$ is compact and the inner integral, if finite, is continuous in ***v***. It thus suffices to show that the inner integral is finite at every single $v\in V_{1}$.

Fixing $v\in V_{1}$, write *A* ≡ *A*(***v***) ≡ ***v***^*a*⊤^**K*v***_*a*_/(2*a*) and *B* ≡ *B*(***v***) ≡ (***η***^⊤^***v***^*b*^)/*b*. We need to show that

$$N(A,B,a,b)\equiv \int_{0}^{\infty }\text{exp}\left(-Az^{2a}+Bz^{b}\right)dz<+\infty .$$

Recall that (CC1) ***v***^⊤^**K*v*** > 0 for all $v\in R_{+}^{m}\setminus \{0\}$, so *A* > 0.

- Suppose *B* ≤ 0. Then $N(A,B,a,b)\leq \int_{0}^{\infty }\text{exp}(-Az^{2a})\;dz=A^{-a∕2}\Gamma (1+1∕(2a))$, a finite constant since *A* > 0 and *a* > 0.
- Suppose *B* > 0. We first want to bound exp(−*Az*^2*a*^ + *Bz*^*b*^) ≤ *N*_0_ exp(−*Az*^2*a*^/2) by some finite constant *N*_0_ > 0, so that $N(A,B,a,b)\leq N_{0}\int_{0}^{\infty }\text{exp}(-Az^{2a}∕2)\;dz$, a finite constant for *a* > 0. Thus, it remains to give conditions so that exp(−*Az*^2*a*^/2 + *Bz^b^*) is bounded by some finite constant *N*_0_, which by continuity only requires a finite limit as *z* ↘ 0 and as *z* ↗ +∞. As *z* ↗ +∞, *Bz^b^* ↗ +∞, while −*Az*^2*a*^/2 ↘ −∞. We thus need *b* < 2*a* so that the sum of the two does not go to positive infinity. On the other hand, as *z* ↘ 0, −*Az*^2*a*^/2 ↗ 0, so we need *b* > 0, otherwise *z^b^* ↗ +∞. In conclusion, we require that 2*a* > *b* > 0.

It thus suffices to require (CC1) and (CC2) 2*a* > *b* > 0 to eliminate restrictions on *B*, and hence on ***η***. That is, ***η*** can take value in the entirety of $R^{m}$.

*Case b* = 0: Again in (CC1) we assume ***v***^⊤^**K*v*** > 0 for all $v\in R_{+}^{m}\setminus \{0\}$. Since $V_{2}\equiv \{v\;:\|v{\|}_{2}=1,v\in R_{+}^{m}\}$ is compact and ***v***^⊤^**K*v*** is continuous in ***v*** and strictly positive on $V_{2}$, the image of $V_{2}$ under ***v***^⊤^**K*v*** is a compact subset of (0, ∞), i.e. $N_{K}\equiv {\text{min}}_{v\in R_{+}^{m}\setminus \{0\}}\;v^{⊤}Kv∕v^{⊤}v\equiv {\text{min}}_{v\in V_{2}}v^{⊤}Kv>0$. We thus have

$$\int_{R_{+}^{m}}\text{exp}\left(\eta^{⊤}\;\log(x)-\frac{1}{2a}x^{a⊤}Kx^{a}\right)dx\;\leq \int_{R_{+}^{m}}\text{exp}\left(\eta^{⊤}\;\log(x)-\frac{N_{K}}{2a}x^{a⊤}x^{a}\right)dx\;=\prod_{j=1}^{m}\int_{0}^{\infty }\text{exp}\left(\eta_{j}\;\log(x_{j})-\frac{N_{K}}{2a}x_{j}^{2a}\right)dx_{j}\;=\prod_{j=1}^{m}\left[\Gamma \left(\frac{\eta_{j}+1}{2a}\right)\frac{(N_{K}∕2a{)}^{-\frac{\eta_{j}+1}{2a}}}{2a}\right],$$

where the integration follows by change of variable and requires *a* > 0. Assuming *a* > 0, the last quantity is finite if and only if ***η*** ≻ −**1**_*m*_, by definition of the gamma function.

In conclusion, given conditions (CC1) ${\text{min}}_{v\in R_{+}^{m}\setminus \{0\}}\;v^{⊤}Kv>0$, (CC2) 2*a* > *b* > 0, and (CC3) *a* > 0, *b* = 0 and ***η*** ≻ −**1**_*m*_, the unnormalized density (16) has a finite normalizing constant when (CC1) and (CC2) both hold, or (CC1) and (CC3) both hold.

The centered settings, where the term involving ***x***^*b*^ is excluded, can be considered as a special case of both (1) and (2) with ***η*** ≡ **0**, and thus (CC1) and *a* > 0 are sufficient. ■

**Proof** [Proof of Theorem 11] Recall assumptions (A1) and (A2):

(A1)
p0(x)hj(xj)∂jlogp(x)∣xj↘0+xj↗+∞=0,∀x−j∈R+m−1,∀p∈P+;∣

(A2)
$$E_{p_{0}}\|\nabla \;\log\;p(X)\;\circ \;h^{1∕2}(X){\|}_{2}^{2}<+\infty ,\;E_{p_{0}}\|(\nabla \;\log\;p(X)\;\circ \;h(X){)}^{\prime }{\|}_{1}<+\infty ,\;\forall p\in P_{+}.$$

Let **K**_0_ and ***η***_0_ be the true parameters so that $p_{0}\in P_{+}$, with $P_{+}$ corresponding to a parameter space in which all parameters satisfy the conditions for a finite normalizing constant. We now give sufficient conditions for *h* to satisfy (A1) and (A2).

##### Conditions for (A1):

Fix *j* = 1,…, *m* and $x_{-j}\in R_{+}^{m-1}$. We show that the conditions on *h_j_* imply that the limits go to 0 as *x_j_* ↗ +∞ and as *x_j_* ↘ 0^+^, which is stronger than (A1); in fact, from (37) below, the limits cannot go to a nonzero finite constant assuming an *h* with polynomial tail, since *a* > 0 and *B*_1_ ≡ *κ*_0,*jj*_ > 0 for all *j*. Now,

(37)
$$p_{0}(x)h_{j}(x_{j})\partial_{j}\;\log\;p(x)\;\propto h_{j}(x_{j})\;\text{exp}\left(-\frac{1}{2a}x^{a⊤}K_{0}x^{a}+\eta_{0}^{⊤}\frac{x^{b}-1_{m}}{b}\right)\partial_{j}\left(-\frac{1}{2a}x^{a⊤}Kx^{a}+\eta^{⊤}\frac{x^{b}-1_{m}}{b}\right)\;\propto h_{j}(x_{j})\;\text{exp}\left(-\frac{1}{a}(k_{0,j,-j}^{⊤}x_{-j}^{a})x_{j}^{a}-\frac{\kappa_{0,jj}}{2a}x_{j}^{2a}+\eta_{0,j}\frac{x_{j}^{b}-1}{b}\right)\times \;\;\left(-k_{j,-j}^{⊤}x_{-j}^{a}x_{j}^{a-1}-\kappa_{jj}x_{j}^{2a-1}+\eta_{j}x_{j}^{b-1}\right)\;\equiv h_{j}(x_{j})\;\text{exp}\left(\frac{A_{1}x_{j}^{a}}{a}+\frac{B_{1}x_{j}^{2a}}{2a}+C_{1}\frac{x_{j}^{b}-1}{b}\right)\left(A_{2}x_{j}^{a-1}+B_{2}x_{j}^{2a-1}+C_{2}x_{j}^{b-1}\right),$$

where $A_{1}\equiv -k_{0,j,-j}^{⊤}x_{-j}^{a}$, $A_{2}\equiv -k_{j,-j}^{⊤}x_{-j}^{a}$, *B*_1_ ≡ −*κ*_0,*jj*_ < 0 and *B*_2_ ≡ −*κ_jj_* < 0 by condition (CC1). Finally *C*_1_ ≡ *η*_0,*j*_, *C*_2_ ≡ *η_j_*.

1. Let *x_j_* ↗ +∞. If *b* > 0, since 2*a* > *b* > 0 and *B*_1_ < 0, the exponential term in (37) decreases to 0 exponentially and its reciprocal dominates any polynomial functions. Thus, the entire product goes to 0 if *h_j_*(*x_j_*) grows no faster than polynomially as *x_j_* ↗ +∞. If *b* = 0, the *C*_1_ log *x_j_* term is again dominated by $B_{1}x_{j}^{2a}∕(2a)$, and the same conclusion holds.
2. Let *x_j_* ↘ 0.
  - Let *b* > 0. Then the exponential term in (37) goes to constant exp(−*C*_1_/*b*), and we only need

(38)
$$\underset{x_{j}↘0^{+}}{\text{lim}}h_{j}(x_{j})(A_{2}x_{j}^{a-1}+B_{2}x_{j}^{2a-1}+C_{2}x_{j}^{b-1})=0.$$

- If *a* > 1 and *b* > 1, the second term in (38) is a polynomial with three terms having powers ≥ min{*a* – 1, *b* – 1}. The product goes to zero if and only if $h_{j}(x_{j})=o(x_{j}^{\max\{1-a,1-b\}})$ as *x_j_* ↘ 0. Note that this is satisfied by any *h_j_* that has a finite right limit at 0.
- If *a* = 1 and *b* ≥ 1, or *a* ≥ 1 and *b* = 1, then the second term in (38) is a polynomial of non-negative power plus a potentially nonzero constant. A sufficient condition for (38) is thus lim_*x_j_* ↘ 0_ *h_j_*(*x_j_*) = 0.
- If *a* < 1 or *b* < 1, then the second part in (38) is a polynomial having terms with negative degree ≥ min{*a* – 1, *b* – 1}. To counteract this a sufficient condition is $h_{j}(x_{j})=o(x_{j}^{\max\{1-a,1-b\}})$.
  In conclusion, lim_*x_j_* ↘ 0_+ *p*_0_(***x***)*h_j_*(*x_j_*)*∂_j_* log *p*(***x***) = 0 if and only if

$$\underset{x_{j}↘0^{+}}{\text{lim}}h_{j}(x_{j})∕x_{j}^{\max\{1-a,1-b\}}=0.$$

- Now assume *b* = 0. Then, (37) now becomes
  $$h_{j}(x_{j})\;\text{exp}\;(A_{1}x_{j}^{a}∕a+B_{1}x_{j}^{2a}∕(2a)+C_{1}\;\log\;x_{j})\;(A_{2}x_{j}^{a-1}+B_{2}x_{j}^{2a-1}+C_{2}∕x_{j}).$$
  With *C*_1_ log *x_j_* dominating, the exponential part scales as $x_{j}^{C_{1}}$. We thus require
  $$\underset{x_{j}↘0^{+}}{\text{lim}}h_{j}(x_{j})(A_{2}x_{j}^{a-1+C_{1}}+B_{2}x_{j}^{2a-1+C_{1}}+C_{2}x_{j}^{C_{1}-1})=0,$$
  which by the previous discussion on (38) holds if and only if
  $$\underset{x_{j}↘0^{+}}{\text{lim}}h_{j}(x_{j})∕x_{j}^{1-C_{1}}=0$$
  since 1 – *a* – *C*_1_ < 1 – *C*_1_.

In summary, (A1) is satisfied if *h_j_*(*x_j_*) grows at most polynomially as *x_j_* ↗ +∞, and ${\text{lim}}_{x_{j}↘0^{+}}\;h_{j}(x_{j})∕x_{j}^{\max\{1-a,1-b\}}=0$ if *b* > 0, or ${\text{lim}}_{x_{j}↘0^{+}}\;h_{j}(x_{j})∕x_{j}^{1-\eta_{0,j}}=0$ if *b* = 0.

##### Conditions for (A2):

For (A2), we consider powers of *x* as the h functions for simplicity; conclusions for other functions that have the same tail behavior (big-O scaling) as *x* ↘ 0 and *x* ↗ +∞ follow similarly. Sufficiency results for piecewise power functions follow by partitioning, and similarly for other functions *h* whose function values and derivatives can be bounded by those of some piecewise power function (e.g. truncated powers), since (A2) is on integrability of products involving positive powers of *h* and *h*′.

Let **K**_0_ and ***η***_0_ be the true parameters from the parameter space that satisfies the conditions for finite normalizing constant. By part (2) of the proof of Theorem 9, the assumption that **K**_0_ satisfies (CC1) implies that ${\text{min}}_{v\in R_{+}^{m}\setminus \{0\}}\;v^{⊤}K_{0}v∕v^{⊤}v\equiv N_{K_{0}}>0$. Then we have the following decomposition

$$p_{K_{0},\eta_{0}}(x)\equiv \text{exp}\left(-\frac{1}{2a}x^{a⊤}K_{0}x^{a}+\eta_{0}^{⊤}\frac{x^{b}-1_{m}}{b}\right)\;\;\leq \prod_{j=1}^{m}\text{exp}\left(-\frac{N_{K_{0}}}{2a}x_{j}^{2a}+\eta_{0,j}\frac{x_{j}^{b}-1}{b}\right).$$

Then for any other **K** and ***η*** in the parameter space, for the first part of (A2) it suffices to show for any *j* = 1,…, *m* that *D* < ∞, where

$$D\equiv \int_{R_{+}^{m}}\prod_{j=1}^{m}\text{exp}\left(-\frac{N_{K_{0}}}{2a}x_{j}^{2a}+\eta_{0,j}\frac{x_{j}^{b}-1}{b}\right)h_{j}(x_{j})\times \;\;{\left(-\kappa_{jj}x_{j}^{2a-1}-\sum_{i\neq j}\kappa_{ji}x_{i}^{a}x_{j}^{a-1}+\eta_{j}x_{j}^{b-1}\right)}^{2}dx\;\;\geq \int_{R_{+}^{m}}p_{0}(x)h_{j}(x_{j})\;(\partial_{j}\;\log\;p(x){)}^{2}\;dx.$$

Note that

$${\left(-\kappa_{jj}x_{j}^{2a-1}-\sum_{i\neq j}\kappa_{ji}x_{i}^{a}x_{j}^{a-1}+\eta_{j}x_{j}^{b-1}\right)}^{2}\;=\kappa_{jj}^{2}x_{j}^{4a-2}+\sum_{i\neq j,\ell \neq j}\kappa_{ji}\kappa_{j\ell }x_{i}^{a}x_{\ell }^{a}x_{j}^{2a-2}+\eta_{j}^{2}x_{j}^{2b-2}+2\sum_{i\neq j}\kappa_{jj}\kappa_{ji}x_{i}^{a}x_{j}^{3a-2}\;\;-2\sum_{i\neq j}\kappa_{ji}\eta_{j}x_{i}^{a}x_{j}^{a+b-2}-2\kappa_{jj}\eta_{j}x_{j}^{2a+b-2}.$$

Thus, plugging this back in the definition of *D*, we can split *D* into a sum of six terms *D*_1_ through *D*_6_, each of which is a sum of terms of the form

$$\int_{R_{+}^{m}}\prod_{k=1}^{m}\text{exp}\left(-\frac{N_{K_{0}}}{2a}x_{k}^{2a}+\eta_{0,k}\frac{x_{k}^{b}-1}{b}\right)h_{j}(x_{j})x_{i}^{{\text{pow}}_{i}}x_{\ell }^{{\text{pow}}_{\ell }}x_{j}^{{\text{pow}}_{j}}\;dx\;=\prod_{k\neq j}\int_{0}^{\infty }\text{exp}\left(-\frac{N_{K_{0}}}{2a}x_{k}^{2a}+\eta_{0,k}\frac{x_{k}^{b}-1}{b}\right)x_{k}^{{\text{pow}}_{k}}\;dx_{k}\;\;\times \int_{0}^{\infty }\text{exp}\left(-\frac{N_{K_{0}}}{2a}x_{j}^{2a}+\eta_{0,j}\frac{x_{j}^{b-1}}{b}\right)h_{j}(x_{j})x_{j}^{{\text{pow}}_{j}}\;dx_{j}$$

times a constant involving **K** and *η_j_*, where pow_*k*_ ≥ 0 for each *k* ≠ *j*. We have thus decomposed the integral into a product of univariate integrals. Note that

$$\int_{0}^{\infty }\text{exp}\left(-\frac{N_{K_{0}}}{2a}x_{i}^{2a}+\eta_{0,i}\frac{x_{i}^{b}-1}{b}\right)x_{i}^{{\text{pow}}_{i}}\;dx_{i}$$

is finite for all pow_*i*_ ≤ 0 regardless of whether *b* is nonzero, since we assumed **K**_0_ and ***η***_0_ to lie in the parameter space with a finite normalizing constant. Indeed, if *b* > 0 then the terms in the exponential is a regular polynomial with positive degree and a negative leading term; if *b* = 0 then integrability follows from *η*_0,*i*_ + pow_*i*_ ≥ *η*_0,*i*_ > −1. Thus, we only need to consider the univariate integral that involve the *x_j_* terms, namely

$$\int_{0}^{\infty }\text{exp}\left(-\frac{N_{K_{0}}}{2a}x_{j}^{2a}+\eta_{0,j}\frac{x_{j}^{b}-1}{b}\right)h_{j}(x_{j})x_{j}^{{\text{pow}}_{j}}\;dx_{j},$$

where pow_*j*_ takes value in {4*a* – 2, 2*a* – 2, 2*b* – 2, 3*a* – 2, *a* + *b* – 2, 2*a* + *b* – 2} ⊆ [2 min{*a*, *b*} – 2, 4*a* – 2]. We split the integral into two parts over [0, 1] and [1, ∞], respectively.

- If *b* > 0, on [0, 1] the exponential part is bounded above and below by positive constants, and for (A1) we require *h_j_*(*x*) = *o*(*x*^1–min{*a*, *b*}^) as *x* ↘ 0^+^, so the integrand is *o*(*x*^min{*a*, *b*}−1^) = *o*(*x*^−1^) and is thus integrable on [0, 1]. The integrand on [1, ∞) is integrable as in (A1) we assume *h* to grow at most polynomially.
- If *b* = 0, pow_*j*_ ∈ [−2, 4*a* – 2] and the integrand becomes
  $$\text{exp}(-N_{K_{0}}x_{j}^{2a}∕(2a))h_{j}(x_{j})x_{j}^{{\text{pow}}_{j}+\eta_{0,j}}.$$
  On [0, 1], (A1) requires *h_j_*(*x*) = *o*(*x*^1–min_*j*_ *η*_0,*j*_^), so $h_{j}(x_{j})x_{j}^{{\text{pow}}_{j}+\eta_{0,j}}=o(x^{-1})$ and the integrand is again integrable. Integrability on [1, ∞) follows similarly to the case with *b* > 0.

Now consider the second part of (A2). By definition $E_{p_{0}}\|(\nabla \;\log\;p(X)\;\circ \;h(X){)}^{\prime }{\|}_{1}$ equals

∫R+mp0(x)∑j=1m∣hj′(Xj)∂jlogp(X)+hj(Xj)∂j2logp(X)∣dx≤∑j=1m∫R+mexp(−NK02axj2a+η0,jxjb−1b)∣hj′(xj)(−κjjxj2a−1−∑i≠jκjixiaxja−1+ηjxjb−1)∣∣+hj(xj)(−κjj(2a−1)xj2a−2−∑i≠jκji(a−1)xiaxja−2+(b−1)ηjxjb−2)∣dx.

By the triangle inequality and the fact that *h_j_* ≥ 0 and $h_{j}^{\prime }\geq 0$, similar to the proof for the first part, for each *j* the integral can be bounded by a sum of six integrals, each of the form

$$\text{const}\times \int_{R_{+}^{m}}\prod_{k=1}^{m}\text{exp}\left(-\frac{N_{K_{0}}}{2a}x_{k}^{2a}+\eta_{0,k}\frac{x_{k}^{b}-1}{b}\right)h_{j}(x_{j})x_{i}^{{\text{pow}}_{i}}x_{j}^{{\text{pow}}_{j}}\;dx,$$

or with *h_j_* replaced by $h_{j}^{\prime }$. Finiteness thus follows from the same type of discussion by noting that *h_j_*(*x*) = *o*(*x*^1–min{*a*, *b*}^) and $h_{j}^{\prime }(x)=o(x^{-\text{min}\{a,b\}})$.

We conclude that if the true and the proposed parameters give densities with finite normalizing constants, and if *h* satisfies assumption (A1), then (A2) is automatically satisfied.

In the centered case where we assume ***η*** ≡ **0**, we only need ${\text{lim}}_{x_{j}↘0^{+}}\;h_{j}(x_{j})∕x_{j}^{1-a}=0$ as it is a special case with *b* = 2*a*. ■

#### A.4. Proof of Theorems in Section 6

**Proof** [Proof of Corollary 14] By Theorem 13, under assumptions in that theorem, the support of $\hat{\Psi }$ is a subset of the true support of **Ψ**_0_, and $\|\hat{\Psi }-\Psi_{0}{\|}_{\infty }\leq \frac{c_{\Gamma_{0}}}{2-\alpha }\lambda$. Since **Ψ**_0_ has ∣*S*_0_∣ nonzero entries,

$$|\|\hat{\Psi }-\Psi_{0}\|{|}_{F}={\left[\sum_{\Psi_{0,jk}\neq 0}({\hat{\Psi }}_{jk}-\Psi_{0,jk}{)}^{2}\right]}^{1∕2}\leq \sqrt{|S_{0}|}\|\hat{\Psi }-\Psi_{0}{\|}_{\infty }\leq \frac{c_{\Gamma_{0}}}{2-\alpha }\;\lambda \sqrt{|S_{0}|}.$$

Similarly, by the definition of matrix *ℓ*_∞_-*ℓ*_∞_ norm,

$$|\|\hat{\Psi }-\Psi_{0}\|{|}_{2}\leq \||\hat{\Psi }-\Psi_{0}\|{|}_{\infty }=\max_{j=1,\ldots ,m}\sum_{k=1}^{m}|{\hat{\Psi }}_{jk}-\Psi_{0,jk}|\leq \frac{c_{\Gamma_{0}}}{2-\alpha }\;\lambda d_{\Psi_{0}}.$$

The result follows by also noting that $\||\hat{\Psi }-\Psi_{0}|{\|}_{2}\leq \||\hat{\Psi }-\Psi_{0}|{\|}_{F}$. ■

**Proof** [Proof of Theorem 15] The proof is based on Theorem 13 and a probabilistic bound on ∥**Γ**_***γ***_ – **Γ**_0_∥_∞_, where in the case of centered Gaussian **Γ** = diag(**xx**^⊤^,…,**xx**^⊤^). Denote $\Sigma_{0}=K_{0}^{-1}$. In particular, given *τ* > 2 we wish to show that for $\epsilon =80\sqrt{2}c_{0}\;\max_{j}(\Sigma_{0,jj})$, assuming $c_{0}\equiv \sqrt{(\tau \;\log\;m+\log\;4)∕n}<1∕\sqrt{2}$,

$$P\left(|n^{-1}\sum_{i=1}^{n}X_{j}^{\left(i\right)}X_{k}^{\left(i\right)}+\gamma_{1j}1_{\left\{j=k\right\}}-EX_{j}X_{k}|>\epsilon \right)\leq m^{2-\tau },$$

and so the results follow from Theorem 13.

By Lemma 1 of Ravikumar et al. (2011), since $X_{j}∕\sqrt{\Sigma_{0,jj}}$ is Gaussian with mean 0 and standard deviation 1,

$$P\left(|n^{-1}\sum_{i=1}^{n}X_{j}^{\left(i\right)}X_{k}^{\left(i\right)}-EX_{j}X_{k}|>t\right)\leq 4\;\text{exp}\left(-\frac{nt^{2}}{3200\;\max_{j}(\Sigma_{0,jj}{)}^{2}}\right)$$

for *t* ∈ (0, 40 max_*j*_ (Σ_0,*jj*_)). Denote the event as *ℇ*_*j,k*_(*t*). Note that $EX_{j}^{2}\leq \max_{j}\;\Sigma_{0,jj}=\epsilon ∕(80\sqrt{2}c_{0})$. Then letting *t* = *ϵ*/2 and conditioning on the complement of *ℇ*_*j,j*_(*ϵ*/2), we have

$$n^{-1}\sum_{i=1}^{n}X_{j}^{(i{)}^{2}}\leq EX_{j}^{2}+\epsilon ∕2\leq \frac{\epsilon }{2}\left(1+\frac{1}{40\sqrt{2}c_{0}}\right).$$

Thus, choosing $\gamma_{\ell j}=(\delta -1)\sum_{i=1}^{n}X_{j}^{(i{)}^{2}}∕n$ for *ℓ* = 1,…, *m* (**Γ** has *m* identical blocks) with $1<\delta <1+(1+1∕(40\sqrt{2}c_{0}){)}^{-1}$, by the triangle inequality and a union bound we have

$$P\left(\max_{j,k}|n^{-1}\sum_{i=1}^{n}X_{j}^{\left(i\right)}X_{k}^{\left(i\right)}+\gamma_{1j}1_{\left\{j=k\right\}}-EX_{j}X_{k}|>\epsilon \right)\leq P(E_{j,k}(\epsilon ∕2))=m^{2-\tau }.$$

Since *τ* > 2, it holds that $1+(1+1∕(40\sqrt{2}c_{0}){)}^{-1}=2-(1+40\sqrt{2}c_{0}{)}^{-1}$ is larger than $2-(1+80\sqrt{\log\;m∕n}{)}^{-1}\equiv C(n,m)$, so it is safe to choose any *δ* ∈ (1, *C*(*n*, *m*)). Thus by the requirement on *ϵ*, the theorem statement holds when $n>\max(c^{*}c_{1}^{2}d_{K}^{2},2)(\tau \;\log\;m+\log\;4)$ with *c** = 12800 max_*j*_(Σ_0,*jj*_)^2^. ■

**Proof** [Proof of Theorem 16] The proof of Theorem 13 from Lin et al. (2016) does not rely on the fact that the original **Γ** is an unbiased estimator for the population **Γ**_0_, but instead only requires one to bound ∥**Γ** – **Γ**_0_∥_∞_. Thus, for **Γ_*γ*_** = **Γ** + diag(***γ***), by Theorem 13 it suffices to prove that for any *τ* > 3, we can bound ∥**Γ**(**x**) + diag(***γ***(**x**)) – **Γ**_0_∥_∞_ by some *ϵ*_1_ and ∥***g***(**x**) – ***g***_0_∥_∞_ by some *ϵ*_2_, uniformly with probability 1 – *m*^3–*τ*^. Recall from (21) that the *j*^th^ block of $\Gamma_{\gamma }\in R^{m^{2}\times m^{2}}$ has (*k*, *ℓ*)-th entry

$$n^{-1}\sum_{i=1}^{n}X_{k}^{\left(i\right)}X_{\ell }^{\left(i\right)}h_{j}\left(X_{j}^{\left(i\right)}\right)+\gamma_{kj}\cdot 1_{\left\{k=\ell \right\}}.$$

The entry in $g\in R^{m^{2}}$ (obtained by linearizing a *m* × *m* matrix) corresponding to (*j*, *k*) is

$$n^{-1}\sum_{i=1}^{n}X_{k}^{\left(i\right)}h_{j}^{\prime }\left(X_{j}^{\left(i\right)}\right)+n^{-1}1_{\left\{j=k\right\}}\sum_{i=1}^{n}h_{j}\left(X_{j}^{\left(i\right)}\right).$$

Denote *M* ≡ max_*j*_ sup_*x*>0_ *h_j_*(*x*), $M^{\prime }\equiv \max_{j}\;{\text{sup}}_{x>0}h_{j}^{\prime }(x)$, and $c_{X}\equiv 2\;\max_{j}(2\sqrt{\Sigma_{jj}}+\sqrt{e}\;E_{0}X_{j})$. Using results for sub-Gaussian random variables from Lemma 22.2 in Appendix B, we have for any *t*_1_ > 0,

$$P\left(|n^{-1}\sum_{i=1}^{n}X_{k}^{\left(i\right)}X_{\ell }^{\left(i\right)}h_{j}\left(X_{j}^{\left(i\right)}\right)-E_{0}X_{k}X_{\ell }h_{j}(X_{j})|>t_{1}\right)\leq 2\;\text{exp}\left(-\;\text{min}\left(\frac{nt_{1}^{2}}{2M^{2}c_{X}^{4}},\frac{nt_{1}}{2Mc_{X}^{2}}\right)\right).$$

Thus, choosing $\epsilon_{1}\equiv 2Mc_{X}^{2}c_{n,m}$, where $c_{n,m}\equiv \max\left\{\frac{2(\log\;m^{\tau }+\log\;6)}{n},\sqrt{\frac{2(\log\;m^{\tau }+\log\;6)}{n}}\right\}$, for *γ_kj_* ≤ *ϵ*_1_/2, we have

(39)
$$P\left(|n^{-1}\sum_{i=1}^{n}X_{k}^{\left(i\right)}X_{\ell }^{\left(i\right)}h_{j}\left(X_{j}^{\left(i\right)}\right)+\gamma_{kj}1_{\left\{k=\ell \right\}}-E_{0}X_{k}X_{\ell }h_{j}(X_{j})|>\epsilon_{1}\right)\;\leq P\left(|n^{-1}\sum_{i=1}^{n}X_{k}^{\left(i\right)}X_{\ell }^{\left(i\right)}h_{j}\left(X_{j}^{\left(i\right)}\right)-E_{0}X_{k}X_{\ell }h_{j}(X_{j})|>\epsilon_{1}∕2\right)$$

(40)
$$\leq 2\;\text{exp}\left(-\;\text{min}\left(\frac{n\epsilon_{1}^{2}}{8M^{2}c_{X}^{4}},\frac{n\epsilon_{1}}{4Mc_{X}^{2}}\right)\right)\leq \frac{1}{3m^{\tau }}.$$

Denote the event inside the probability in (39) as *ℇ*_*k*,*ℓ*,*j*_ (*ϵ*_1_/2).

By definition,

$$c_{X}^{2}=4\;\max_{k}\left(4\Sigma_{kk}+4\sqrt{e}\sqrt{\Sigma_{kk}}\;E_{0}X_{k}+e(E_{0}X_{k}{)}^{2}\right)\geq 4e\;\max_{k}\left(\Sigma_{kk}+(E_{0}X_{k}{)}^{2}\right).$$

By Lemmas 21.2 and 22.1 from Appendix B, var(*X_k_*) ≤ Σ_*kk*_, so $c_{X}^{2}\geq 4e\;\max_{k}\;E_{0}X_{k}^{2}\geq 4eE_{0}X_{k}^{2}h_{j}(X_{j})∕M$. Thus, setting $\epsilon_{1}\equiv 2Mc_{X}^{2}c_{n,m}$ on the complement of *ℇ*_*k*,*k*,*j*_(*ϵ*_1_/2) we have

$$n^{-1}\sum_{i=1}^{n}X_{k}^{(i{)}^{2}}h_{j}\left(X_{j}^{\left(i\right)}\right)\leq E_{0}X_{k}^{2}h_{j}(X_{j})+\epsilon_{1}∕2\leq \frac{\epsilon_{1}}{2}\left(1+\frac{1}{4ec_{n,m}}\right).$$

Then

(41)
$$\frac{1}{1+1∕(4ec_{n,m})}\frac{1}{n}\sum_{i=1}^{n}X_{k}^{(i{)}^{2}}h_{j}\left(X_{j}^{\left(i\right)}\right)\leq \epsilon_{1}∕2$$

on the complement of *ℇ*_*k*,*k*,*j*_(*ϵ*_1_/2), again with $c_{n,m}\equiv \max\left\{\frac{2(\log\;m^{\tau }+\log\;6)}{n},\sqrt{\frac{2(\log\;m^{\tau }+\log\;6)}{n}}\right\}$. Note that the multiplier on the left of (41) is increasing in *c*_*n*, *m*_, and that 2(log *m*^*τ*^ + log6) > 6 log *m* by the assumption that *τ* > 3. Thus, if we let

$$\gamma_{kj}\equiv \frac{1}{1+1∕\left(4e\;\max\left\{6\;\log\;m∕n,\sqrt{6\;\log\;m∕n}\right\}\right)}\frac{1}{n}\sum_{i=1}^{n}X_{k}^{(i{)}^{2}}h_{j}\left(X_{j}^{\left(i\right)}\right),$$

which is just a constant multiple of the (*k*, *k*)-th entry of **Γ**_*j*_ itself, with the constant explicitly calculable and a function of *p* and *n* only, then for *k* = *ℓ*

$$P\left(|n^{-1}\sum_{i=1}^{n}X_{k}^{\left(i\right)}X_{\ell }^{\left(i\right)}h_{j}\left(X_{j}^{\left(i\right)}\right)+\gamma_{kj}-E_{0}X_{k}X_{\ell }h_{j}(X_{j})|\geq \epsilon_{1}\right)\leq P(E_{k,\ell ,j}(\epsilon_{1}∕2))\leq \frac{1}{3m^{\tau }}.$$

Since this also holds for *k* ≠ *ℓ* without the *γ*_*kj*_ term, by a union bound over *m*^3^ events,

(42)
$$P\left(\max_{j,k,\ell }|n^{-1}\sum_{i=1}^{n}X_{k}^{\left(i\right)}X_{\ell }^{\left(i\right)}h_{j}\left(X_{j}^{\left(i\right)}\right)+\gamma_{kj}1_{\left\{k=\ell \right\}}-E_{0}X_{k}X_{\ell }h_{j}(X_{j})|\geq \epsilon_{1}\right)\leq \frac{1}{3m^{\tau -3}}.$$

Now, on the other hand, Lemma 22.1 and Hoeffding’s inequality give for any *t*_2,1_, *t*_2,2_ > 0 that

$$P\left(|n^{-1}\sum_{i=1}^{n}X_{k}^{\left(i\right)}h_{j}^{\prime }\left(X_{j}^{\left(i\right)}\right)-E_{0}X_{k}h_{j}^{\prime }(X_{j})|\geq t_{2,1}\right)\leq 2\;\text{exp}\left(-\frac{nt_{2,1}^{2}}{2M^{\prime 2}c_{X}^{2}}\right),\;P\left(|n^{-1}\sum_{i=1}^{n}h_{j}\left(X_{j}^{\left(i\right)}\right)-E_{0}h_{j}(X_{j})|\geq t_{2,2}\right)\leq 2\;\text{exp}\;(-2nt_{2,2}^{2}∕M^{2}).$$

Choosing $\epsilon_{2,1}\equiv \sqrt{2}M^{\prime }c_{X}\sqrt{\frac{\log\;m^{\tau -1}+\log\;6}{n}}$, $\epsilon_{2,2}\equiv M\sqrt{\frac{\log\;m^{\tau -2}+\log\;6}{2n}}$ and taking union bounds over *m*^2^, and *m* events, respectively, we have

(43)
$$P\left(\max_{j,k}|n^{-1}\sum_{i=1}^{n}X_{k}^{\left(i\right)}h_{j}^{\prime }\left(X_{j}^{\left(i\right)}\right)-E_{0}X_{k}h_{j}^{\prime }(X_{j})|\geq \epsilon_{2,1}\right)\leq \frac{1}{3m^{\tau -3}},$$

(44)
$$P\left(\max_{j}|n^{-1}\sum_{i=1}^{n}h_{j}\left(X_{j}^{\left(i\right)}\right)-E_{0}h_{j}(X_{j})|\geq \epsilon_{2,2}\right)\leq \frac{1}{3m^{\tau -3}}.$$

Hence, by (42) (43) (44), with probability at least 1 – *m*^3–*τ*^, ∥**Γ**_***γ***_(**x**) – **Γ**_0_∥_∞_ < *ϵ*_1_ and ∥***g***(**x**) – ***g***_0_∥_∞_ < *ϵ*_2_ ≡ *ϵ*_2,1_ + *ϵ*_2,2_. Consider any *τ* > 3, and let

c2≡6αcΓ0,n>max{2M2cX2c22dK02(τlogm+log6),2McX2c2dK0(τlogm+log6)},λ>3(2−α)αmax{cK0ϵ1,ϵ2}≡3(2−α)αmax{4McK0cX2(logmτ+log6)n},{2McK0cX22(logmτ+log6)n,2M′cXlogmτ−1+log6n+Mlogmτ−2+log62n}.

Then *d*_**K**_0__*ϵ*_1_ ≤ *α*/(6*c*_**Γ**_0__) and the results follow from Theorem 13. ■

**Proof** [Proof of Theorem 17] Similar to the proof of Theorem 16, by Theorem 13 it suffices to prove that for any *τ* > 3, we can bound ∥**Γ_*γ*_**(**x**) – **Γ**_0_∥_∞_ by some *ϵ*_1_ and ∥***g***(**x**) – ***g***_0_∥_∞_ by some *ϵ*_2_, uniformly with probability 1 – *m*^3–*τ*^. Recall that $\Gamma \in R^{\left(m^{2}+m)\times (m^{2}+m\right)}$ is a rearrangement of **Γ**^(*)^ which is in turn formed by $\Gamma_{11}\in R^{m^{2}\times m^{2}}$, $\Gamma_{12}\in R^{m^{2}\times m}$, $\Gamma_{12}^{⊤}$ and $\Gamma_{22}\in R^{m\times m}$, all of which are block-diagonal with *m* blocks.

The *j*^th^ block of $\Gamma_{11}\in R^{m^{2}\times m^{2}}$ has (*k*, *ℓ*)-th entry

$$n^{-1}\sum_{i=1}^{n}X_{k}^{\left(i\right)}X_{\ell }^{\left(i\right)}h_{j}\left(X_{j}^{\left(i\right)}\right),$$

the *k*^th^ entry in the *j*^th^ block of **Γ**_12_ is

$$-n^{-1}\sum_{i=1}^{n}X_{k}^{\left(i\right)}h_{j}\left(X_{j}^{\left(i\right)}\right),$$

the *j*^th^ diagonal entry of **Γ**_22_ is

$$n^{-1}\sum_{i=1}^{n}h_{j}\left(X_{j}^{\left(i\right)}\right).$$

On the other hand, $g\in R^{\left(m^{2}+m\right)}$ is a rearrangement of $g^{\left(*\right)}\equiv [g_{1}^{⊤},g_{2}^{⊤}{]}^{⊤}$, where the entry in $g_{1}\in R^{m^{2}}$ (obtained by linearizing a *m* × *m* matrix) corresponding to (*j*, *k*), is

$$n^{-1}\sum_{i=1}^{n}X_{k}^{\left(i\right)}h_{j}^{\prime }\left(X_{j}^{\left(i\right)}\right)+n^{-1}1_{\left\{j=k\right\}}\sum_{i=1}^{n}h_{j}\left(X_{j}^{\left(i\right)}\right),$$

while the *j*-th component of $g_{2}\in R^{m}$ is

$$-n^{-1}\sum_{i=1}^{n}h_{j}^{\prime }\left(X_{j}^{\left(i\right)}\right).$$

Recalling that the bounds in Lemma 22 also hold when ***μ*** ≠ 0, we may then use bounds similar to those in the proof of Theorem 16, and use union bounds to arrive at analogous consistency results, modulus different constants. The amplifiers ***γ*** can be incorporated analogously. ■

### Appendix B. Auxiliary Lemmas and Definitions

In this appendix, to simplify notation, when it is clear from the context, the operator E is defined as the expectation under the true distribution, unless otherwise noted.

Definition 20 (Sub-Gaussian and Sub-Exponential Variables)

*The* sub-Gaussian (*r* = 2) *and* sub-exponential (*r* = 1) norms *of a random variable are*

$$\|X{\|}_{\psi_{r}}\equiv \underset{q\geq 1}{\text{sup}}q^{-1∕r}(E|X{|}^{rq}{)}^{1∕(rq)}\equiv \underset{q\geq 1}{\text{sup}}q^{-1∕r}\|X{\|}_{rq}.$$

*If* ∥*X*∥_*ψ*_2__ < ∞ *we say X is* sub-Gaussian; *if* ∥*X*∥_*ψ*_1__ < ∞ *we call X* sub-exponential. *For a* zero-mean *sub-Gaussian random variable X also define the* sub-Gaussian parameter

$$\tau (X)=\text{inf}\{\tau \geq 0\;:E\;\text{exp}(tX)\leq \text{exp}(\tau^{2}t^{2}∕2),\forall t\in R\}.$$

The definition of sub-Gaussian norm here allows for a non-centered variable and differs from the one in Vershynin (2012), which uses ∥*X*∥_*q*_. Instead, it coincides with *θ*_2_ in Buldygin and Kozachenko (2000). The sub-Gaussian parameter is defined as in Buldygin and Kozachenko (2000) and the sub-exponential norm as in Vershynin (2012).

Lemma 21 (Properties of Sub-Gaussian and Sub-Exponential Variables)

1. *For any X and r* = 1,2, $\|X-EX{\|}_{\psi_{r}}\leq 2\|X{\|}_{\psi_{r}}$. *and* $\|X{\|}_{\psi_{r}}\leq \|X-EX{\|}_{\psi_{r}}+|EX|$*, as long as the expectation and norms are finite*.
2. (Buldygin and Kozachenko, 2000) *τ*(*X*) *is a norm on the space of all zero-mean sub-Gaussian variables; so τ*(*X* + *Y*) ≤ *τ*(*X*) + *τ*(*Y*). *If X is zero-mean sub-Gaussian, then* var(*X*) ≤ *τ*^2^(*X*), $\|X{\|}_{\psi_{2}}\leq 2\tau (X)∕\sqrt{e}$, $\tau (X)\leq \sqrt{e}\|X{\|}_{\psi_{2}}$. *If X*_1_,…, *X_n_* *are i.i.d. zero-mean sub-Gaussian,* $\tau (n^{-1}\sum_{i=1}^{n}X_{i})\leq n^{-1∕2}\tau (X_{i})$.
3. *If X*_1_ *and X*_2_ *are sub-Gaussian (not necessarily independent) with* ∥*X*_1_∥_*ψ*_2__ ≤ *K*_1_ *and* ∥*X*∥_*ψ*_2__ ≤ *K*_2_*, then* *X*_1_*X*_2_ *is sub-exponential with* ∥*X*_1_*X*_2_∥_*ψ*_1__ ≤ *K*_1_*K*_2_.
4. (Buldygin and Kozachenko, 2000) *If X is zero-mean sub-Gaussian and q* > 0*, then*
  $$E|X{|}^{q}\leq 2(q∕e{)}^{q∕2}\tau^{q}(X).$$
5. (Buldygin and Kozachenko, 2000) *If X*_1_,…,*X_n_ are independent zero-mean, sub-Gaussian variables, then for any ϵ* > 0,
  $$P(|X_{1}|\geq \epsilon )\leq 2\;\text{exp}\left(-\frac{\epsilon^{2}}{2\tau^{2}(X_{1})}\right),\;P\left(|n^{-1}\sum_{i=1}^{n}X_{i}|\geq \epsilon \right)\leq 2\;\text{exp}\left(-\frac{n\epsilon^{2}}{2\;\max_{i}\;\tau^{2}(X_{i})}\right).$$
6. (Vershynin, 2012) *If X*_1_,…,*X_n_ are independent zero-mean sub-exponential random variables with K* ≥ max_*i*_ ∥*X_i_*∥_*ψ*_1__*, then for any ϵ* > 0,
  $$P(|X_{1}|\geq \epsilon )\leq 2\;\text{exp}\left(-\;\text{min}\left(\frac{\epsilon^{2}}{8e^{2}K^{2}},\frac{\epsilon }{4eK}\right)\right),\;P\left(|n^{-1}\sum_{i=1}^{n}X_{i}|\geq \epsilon \right)\leq 2\;\text{exp}\left(-\;\text{min}\left(\frac{n\epsilon^{2}}{8e^{2}K^{2}},\frac{n\epsilon }{4eK}\right)\right).$$
7. *(Boucheron et al., 2013) If for X*_*i*_ *i.i.d. there exists some B* > 0 *such that*
  $$\underset{q\geq 2}{\text{sup}}{\left(\frac{E|X{|}^{q}}{q!}\right)}^{1∕q}\leq B∕2$$
  *then for all ϵ* > 0,
  $$P\left(|n^{-1}\sum_{i=1}^{n}(X_{i}-EX_{i})|\geq \epsilon \right)\leq 2\;\text{exp}\left(-\;\text{min}\left(\frac{n\epsilon^{2}}{2B^{2}},\frac{n\epsilon }{2B}\right)\right).$$

Proof

1) For *r* = 1, 2, by the triangle inequality, $\|X-EX{\|}_{\psi_{r}}\leq \|X{\|}_{\psi_{r}}+\|EX{\|}_{\psi_{r}}=\|X{\|}_{\psi_{r}}+|EX|\leq \|X{\|}_{\psi_{r}}+E|X|\leq 2\|X{\|}_{\psi_{r}}$, where in the last step we used the definition of ∥ η ∥_*ψ_r_*_ with *q* = 1 for *r* = 1 and $E|X|\leq (E|X{|}^{2}{)}^{1∕2}$ with *q* = 2 for *r* = 2. On the other hand, $\|X{\|}_{\psi_{r}}\leq \|X-EX{\|}_{\psi_{r}}+\|EX{\|}_{\psi_{r}}=\|X-EX\|\psi_{r}+|EX|$.

2) These follow from Theorems 1.2 and 1.3 and Lemmas 1.2 and 1.7 from Buldygin and Kozachenko (2000), and $\sqrt[{4}]{3.1}e^{9∕16}∕\sqrt{2}\approx 1.6467\leq 1.6487\approx \sqrt{e}$.

3) By Hölder’s inequality (or Cauchy-Schwarz),

$$\|X_{1}X_{2}{\|}_{\psi_{1}}=\underset{q\geq 1}{\text{sup}}q^{-1}(E|X_{1}X_{2}{|}^{q}{)}^{1∕q}=\underset{q\geq 1}{\text{sup}}q^{-1}(E|X_{1}^{q}X_{2}^{q}|{)}^{1∕q}\;\;\leq \underset{q\geq 1}{\text{sup}}q^{-1}{\left[(E|X_{1}{|}^{2q}{)}^{1∕2}(E|X_{2}{|}^{2q}{)}^{1∕2}\right]}^{1∕q}\;\;\leq \underset{q\geq 1}{\text{sup}}\left[q^{-1∕2}(E|X_{1}{|}^{2q}{)}^{1∕2q}\right]\underset{q\geq 1}{\text{sup}}\left[q^{-1∕2}(E|X_{2}{|}^{2q}{)}^{1∕2q}\right]\;\;=\|X_{1}{\|}_{\psi_{2}}\|X_{2}{\|}_{\psi_{2}}\leq K_{1}K_{2}.$$

4-6) These are Lemma 1.4 and Theorem 1.5 in Buldygin and Kozachenko (2000), and a consequence of Corollary 5.17 in Vershynin (2012).

7) By Theorem 2.10 of Boucheron et al. (2013) wherein we let *v* ≡ *nB*^2^/2 and *c* ≡ *B*/2, we have

$$P\left(|n^{-1}\sum_{i=1}^{n}(X_{i}-EX_{i})|\geq \epsilon \right)\leq 2\;\text{exp}\left(-\frac{n\epsilon^{2}}{B^{2}+B\epsilon }\right)$$

for all *ϵ* > 0. (Theorem 2.10 gives an one-sided bound; bound for the other side is obtained by taking *X_i_* = −*X_i_*). The inequality follows by splitting into cases *ϵ* ≤ *B* and *ϵ* > *B*. ■

**Lemma 22** *Suppose **X** follows a truncated normal distribution on* $R_{+}^{m}$ *with parameters **μ** and* **Σ** = **K**^−1^ ≻ **0**. *Let* ***X***^(1)^,…, ***X***^(*n*)^ *be i.i.d. copies of **X**, with j-th component of the (i) i-th copy being* $X_{j}^{\left(i\right)}$. *Then*

1. *For* $\tau (X_{j}-EX_{j})\leq \sqrt{\Sigma_{jj}}$. *That is, the sub-Gaussian parameter of any marginal distribution of **X**, after centering, is bounded by the square root of its corresponding diagonal entry in the covariance parameter* **Σ**. *Then, for any ϵ* > 0,
  $$P\left(|n^{-1}\sum_{i=1}^{n}X_{j}^{\left(i\right)}-EX_{j}|>\epsilon \right)\leq 2\;\text{exp}\left(-\frac{n\epsilon^{2}}{2\Sigma_{jj}}\right).$$
  *In particular, if h*_0_ *is a function bounded by M*_0_, *then for any ϵ* > 0,
  $$P\left(|n^{-1}\sum_{i=1}^{n}X_{j}^{\left(i\right)}h_{0}\left(X_{k}^{\left(i\right)}\right)-EX_{j}h_{0}(X_{k})|\geq \epsilon \right)\leq 2\;\text{exp}\left(-\frac{n\epsilon^{2}}{8M_{0}^{2}(2\sqrt{\Sigma_{jj}}+\sqrt{e}\;EX_{j}{)}^{2}}\right),\;\;\tau \left(n^{-1}\sum_{i=1}^{n}X_{j}^{\left(i\right)}h_{0}\left(X_{k}^{\left(i\right)}\right)-EX_{j}h_{0}(X_{k})\right)\leq \frac{2M_{0}}{\sqrt{n}}\left(2\sqrt{\Sigma_{jj}}+\sqrt{e}\;EX_{j}\right),\;\;{\left\|n^{-1}\sum_{i=1}^{n}X_{j}^{\left(i\right)}h_{0}\left(X_{k}^{\left(i\right)}\right)-EX_{j}h_{0}(X_{k})\right\|}_{\psi_{2}}\leq \frac{4M_{0}}{\sqrt{en}}\left(2\sqrt{\Sigma_{jj}}+\sqrt{e}\;EX_{j}\right).$$
2. *For j*, *k*, *ℓ*, ∈ {1,… ,*p*}, *if h*_0_ *is a function bounded by M*_0_*, then*
  (45)
  $$\|X_{j}X_{k}h_{0}(X_{\ell })-EX_{j}X_{k}h_{0}(X_{\ell }){\|}_{\psi_{1}}\leq \frac{M_{0}}{2e}c_{X}^{2},$$
  *where* $c_{X}\equiv 2\;{\text{max}}_{j}(2\sqrt{\Sigma_{jj}}+\sqrt{e}EX_{j})$. *In particular, for any ϵ* > 0,
  $$P\left(|n^{-1}\sum_{i=1}^{n}X_{j}^{\left(i\right)}X_{k}^{\left(i\right)}h_{0}\left(X_{\ell }^{\left(i\right)}\right)-EX_{j}X_{k}h_{0}(X_{\ell })|>\epsilon \right)\;\;\leq 2\;\text{exp}\left(-\;\text{min}\left(\frac{n\epsilon^{2}}{2M_{0}^{2}c_{X}^{4}},\frac{n\epsilon }{2M_{0}c_{X}^{2}}\right)\right).$$

**Proof** [Proof of Lemma 22]

1. Without loss of generality choose *j* = 1. By the definition of sub-Gaussian parameters, we need to show that for all t∈R,

$$E\;\text{exp}(tX_{1})\leq \text{exp}\;(t^{2}\Sigma_{11}∕2+tEX_{1}),$$

which is equivalent to

(46)
$$t^{2}\Sigma_{11}∕2+tEX_{1}-\log\;E\;\text{exp}(tX_{1})\geq 0\;\;\forall t\in R.$$

Since the left-hand side of (46) equals 0 at *t* = 0, it suffices to show that its derivative,

(47)
$$t\Sigma_{11}+EX_{1}-\frac{d\;\log\;E\;\text{exp}(tX_{1})}{dt}=t\Sigma_{11}+EX_{1}-\frac{\frac{dE\;\text{exp}(tX_{1})}{dt}}{E\;\text{exp}(tX_{1})},$$

is non-negative on (0, ∞) and non-positive on (−∞, 0). By properties of moment-generating functions, $\frac{d}{dt}E\text{exp}(tX_{1})$ evaluated at *t* = 0 equals $EX_{1}$, so (47) equals 0 at *t* = 0. It in turn suffices to show the derivative of (47), namely

(48)
$$\Sigma_{11}-\frac{d^{2}\;\log\;E\;\text{exp}(tX_{1})}{dt^{2}}$$

is non-negative in t∈R.

Given any vector $v\in R^{p}$, define $R_{+}^{p}-v\equiv \{u-v:u\in R_{+}^{p}\}$. By Tallis (1961), denoting the first column of **Σ** as **Σ**_1_, the moment-generating function of the marginal distribution of *X*_1_ is

$$\frac{\int_{R_{+}^{p}-(\mu +t\Sigma_{1})}\;\text{exp}\;(-\frac{1}{2}x^{⊤}\Sigma^{-1}x)\;dx}{\int_{R_{+}^{p}-\mu }\;\text{exp}\;(-\frac{1}{2}x^{⊤}\Sigma^{-1}x)\;dx}\;\text{exp}\left(t\mu_{1}+\frac{1}{2}t^{2}\Sigma_{11}\right).$$

(48) thus becomes

$$-\frac{d^{2}}{dt^{2}}\;\log\int_{R_{+}^{p}-(\mu +t\Sigma_{1})}\;\text{exp}\left(-\frac{1}{2}x^{⊤}\Sigma^{-1}x\right)\;dx.$$

Showing this is non-negative in t∈R is equivalent to showing that the integral itself is log-concave in *t*. But

$$\int_{R_{+}^{p}-(\mu +t\Sigma_{1})}\text{exp}\left(-\frac{1}{2}x^{⊤}\Sigma^{-1}x\right)\;dx=\int_{R^{p}}\text{exp}\left(-\frac{1}{2}x^{⊤}\Sigma^{-1}x\right)1_{R_{+}^{p}-\mu }(x+t\Sigma_{1})\;dx$$

with $\text{exp}(-\frac{1}{2}x^{⊤}\Sigma^{-1}x)$ log-concave in ***x*** and $1_{R_{+}^{p}-\mu }(x+t\Sigma_{1})$ log-concave in (***x***, *t*) since $R_{+}^{p}-\mu$ is a convex set. Since log-concavity is closed under multiplication and integration over $R^{p}$, the integral is indeed log-concave, and our proof of the bound on the sub-Gaussian parameter of $X_{j}-EX_{j}$ is complete. The tail bound follows from 5) of Lemma 21. ■

Now by 1) and 2) of Lemma 21,

$$\|X_{j}{\|}_{\psi_{2}}\leq 2\sqrt{\Sigma_{jj}∕e}+EX_{j}.$$

If *h*_0_ is a function bounded by *M*_0_, then by definition

$$\|X_{j}h_{0}(X_{k}){\|}_{\psi_{2}}\leq M_{0}\left(2\sqrt{\Sigma_{jj}∕e}+EX_{j}\right).$$

By 1) and 2) of Lemma 21 again,

$$\tau (X_{j}h_{0}(X_{k})-EX_{j}h_{0}(X_{k}))\leq \sqrt{e}\|X_{j}h_{0}(X_{k})-EX_{j}h_{0}(X_{k}){\|}_{\psi_{2}}\;\;\leq 2\sqrt{e}\|X_{j}h_{0}(X_{k}){\|}_{\psi_{2}}\;\;\leq 2M_{0}(2\sqrt{\Sigma_{jj}}+\sqrt{e}\;EX_{j}).$$

The tail bound thus follows from the first inequality using 5) of Lemma 21. By 2) of the Lemma 21,

$$\tau \left(n^{-1}\sum_{i=1}^{n}X_{j}^{\left(i\right)}h_{0}\left(X_{k}^{\left(i\right)}\right)-EX_{j}h_{0}(X_{k})\right)\leq \frac{2M_{0}}{\sqrt{n}}\left(2\sqrt{\Sigma_{jj}}+\sqrt{e}\;EX_{j}\right),\;{\left\|n^{-1}\sum_{i=1}^{n}X_{j}^{\left(i\right)}h_{0}\left(X_{k}^{\left(i\right)}\right)-EX_{j}h_{0}(X_{k})\right\|}_{\psi_{2}}\leq \frac{4M_{0}}{\sqrt{en}}\left(2\sqrt{\Sigma_{jj}}+\sqrt{e}\;EX_{j}\right).$$

2. By the proof of 1) of this lemma, $\|X_{j}{\|}_{\psi_{2}}\leq 2\sqrt{\Sigma_{jj}∕e}+EX_{j}$, and by 3) of Lemma 21,

$$\|X_{j}X_{k}{\|}_{\psi_{1}}\leq \left(2\sqrt{\Sigma_{jj}∕e}+EX_{j}\right)\left(2\sqrt{\Sigma_{kk}∕e}+EX_{k}\right)\leq \max_{j}{\left(2\sqrt{\Sigma_{jj}∕e}+EX_{j}\right)}^{2}.$$

Since *h*_0_ is a function bounded by *M*_0_, by definition

$$\|X_{j}X_{k}h_{0}(X_{\ell }){\|}_{\psi_{1}}\leq M_{0}\max_{j}{\left(2\sqrt{\Sigma_{jj}∕e}+EX_{j}\right)}^{2}.$$

Then by 1) of Lemma 21 again,

$$\|X_{j}X_{k}h_{0}(X_{\ell })-EX_{j}X_{k}h_{0}(X_{\ell }){\|}_{\psi_{1}}\leq 2M_{0}\max_{j}{\left(2\sqrt{\Sigma_{jj}∕e}+EX_{j}\right)}^{2}.$$

The tail bound then follows from 6) of Lemma 21. ■

Although not used for our consistency results, in the special case of *h*_0_ ≡ 1, we also have the following lemma. The notable difference between bounds (49) below and (45) from Lemma 22.2 is in the constants and dependency on $EX_{j}$: The constants in the denominator in the right-hand side of (45) is smaller and thus gives a tighter bound, but (49) is preferred when $EX_{j}$ is notably large compared to $\sqrt{\Sigma_{jj}}$, since the constant is only linear in $EX_{j}$.

**Lemma 23** *Consider the setting in Lemma 22. Then for j, k* ∈ {1,… ,*p*}, *for any ϵ* > 0,

(49)
$$P\left(n^{-1}|\sum_{i=1}^{n}X_{j}^{\left(i\right)}X_{k}^{\left(i\right)}-EX_{j}X_{k}|\geq \epsilon \right)\leq 4\;\text{exp}\left(-\;\text{min}\left(\frac{2n\epsilon^{2}}{C_{1}^{2}},\frac{n\epsilon }{C_{1}}\right)\right),$$

*where* $C_{1}\equiv 91{\text{max}}_{j}\;\Sigma_{jj}+72\;{\text{max}}_{j}\;EX_{j}\;{\text{max}}_{j}\sqrt{\Sigma_{jj}}$.

**Proof** [Proof of Lemma 23] We use a proof similar to Lemma 1 in Ravikumar et al. (2011) (note that $EX_{j}$ may be nonzero in our case). Define

$$U_{jk}^{\left(i\right)}\equiv X_{j}^{\left(i\right)}+X_{k}^{\left(i\right)},\;U_{jk}\equiv X_{j}+X_{k},\;V_{jk}^{\left(i\right)}\equiv X_{j}^{\left(i\right)}-X_{k}^{\left(i\right)},\;V_{jk}\equiv X_{j}-X_{k}.$$

Since $X_{j}^{\left(i\right)}X_{k}^{\left(i\right)}=\frac{1}{4}\left(U_{jk}^{(i{)}^{2}}-V_{jk}^{(i{)}^{2}}\right)$, by union bound we have

$$P\left(|n^{-1}\sum_{i=1}^{n}X_{j}^{\left(i\right)}X_{k}^{\left(i\right)}-EX_{j}X_{k}|\geq \epsilon \right)\;\leq P\left(|n^{-1}\sum_{i=1}^{n}U_{jk}^{(i{)}^{2}}-EU_{jk}^{2}|\geq 2\epsilon \right)+P\left(|n^{-1}\sum_{i=1}^{n}V_{jk}^{(i{)}^{2}}-EV_{jk}^{2}|\geq 2\epsilon \right).$$

We next define

$$Z_{jk}^{\left(i\right)}\equiv U_{jk}^{(i{)}^{2}}-\;EU_{jk}^{2}=A_{jk}^{(i{)}^{2}}+B_{jk}^{\left(i\right)}+C_{jk},\;\;\;\;{\bar{X}}_{j}^{\left(i\right)}\equiv X_{j}^{\left(i\right)}-\;EX_{j},\;A_{jk}^{\left(i\right)}\equiv {\bar{X}}_{j}^{\left(i\right)}+{\bar{X}}_{k}^{\left(i\right)},\;\;B_{jk}^{\left(i\right)}\equiv 2(EX_{j}+EX_{k})({\bar{X}}_{j}^{\left(i\right)}+{\bar{X}}_{k}^{\left(i\right)}),\;\;C_{jk}\equiv -\;E({\bar{X}}_{j}^{\left(i\right)}+{\bar{X}}_{k}^{\left(i\right)}{)}^{2}.$$

Then since *τ* is a norm by 2) of Lemma 21, *A_jk_* is sub-Gaussian with parameter ≤ $\sqrt{\Sigma_{jj}}+\sqrt{\Sigma_{kk}}$, and *B*_*jk*_ is sub-Gaussian with parameter ≤ $2(EX_{j}+EX_{k})(\sqrt{\Sigma_{jj}}+\sqrt{\Sigma_{kk}})$. Using 4) of Lemma 21 together with the inequality (*a* + *b* + *c*)^*q*^ ≤ (3 max{*a*, *b*, *c*})^*q*^ ≤ 3^*q*^(*a^q^* + *b*^*q*^ + *c^q^*) for all *a*, *b*, *c* ≥ 0 and *q* > 0, we have for any *q* ≥ 2

(E∣Zjk∣q)1∕q≤(3q(E∣Ajk∣2q+E∣Bjk∣q+∣Cjk∣q))1∕q≤31+1∕q((E∣Ajk∣2q)1∕q+(E∣Bjk∣q)1∕q+∣Cjk∣)≤31+1∕q(21∕q(2q∕e)(Σjj+Σkk)2)(+21∕qq∕e2(EXj+EXk)(Σjj+Σkk)+var(Xj+Xk)).

Using var(*X* + *Y*) ≤ 2(var(*X*) + var(*Y*)) and the fact that $\text{var}(X_{j})=\text{var}(X_{j}-EX_{j})\leq \tau^{2}(X_{j}-EX_{j})\leq \Sigma_{jj}$ (by 2) of Lemma 21 and 1) of Lemma 22, we then have

$${\left(\frac{E|Z_{jk}{|}^{q}}{q!}\right)}^{1∕q}\;\;\leq 3^{1+1∕q}\frac{2^{3+1∕q}(q∕e)\;\max_{j}\;\Sigma_{jj}+2^{3+1∕q}\sqrt{q∕e}\;\max_{j}\;EX_{j}\cdot \max_{j}\;\sqrt{\Sigma_{jj}}+4\;\max_{j}\;\Sigma_{jj}}{(q!{)}^{1∕q}}.$$

Since all three coefficients involving *q* are decreasing in *q* ≥ 2, we have

$$\underset{q\geq 2}{\text{sup}}{\left(\frac{E|Z_{jk}{|}^{q}}{q!}\right)}^{1∕q}\leq \left(48\sqrt{3}∕e+6\sqrt{6}\right)\max_{j}\;\Sigma_{jj}+24\sqrt{6∕e}\max_{j}EX_{j}\max_{j}\sqrt{\Sigma_{jj}}.$$

Thus by 7) of Lemma 21, letting $B\equiv (91\;{\text{max}}_{j}\;\Sigma_{jj}+72\;{\text{max}}_{j}\;EX_{j}\;{\text{max}}_{j}\;\sqrt{\Sigma_{jj}})$ for all *ϵ* > 0:

$$P\left(n^{-1}|\sum_{i=1}^{n}Z_{jk}^{\left(i\right)}|\geq 2\epsilon \right)\leq 2\;\text{exp}\left(-\;\text{min}\left(\frac{2n\epsilon^{2}}{B^{2}},\frac{n\epsilon }{B}\right)\right).$$

A tail bound for the sample average of $V_{jk}^{2}$ can be similarly derived, and the result follows. ■

### Appendix C. Simulation Results for Erdös-Rényi Graphs

We revisit the simulations from Section 7 but use Erdös-Rényi (ER) graphs in which each possible edge is independently included with probability *π*. Independent uniform draws from [0.5, 1] are used to fill the non-zero off-diagonal entries of the symmetric matrix **K**_0_. The diagonal elements are set such that **K**_0_ has minimum eigenvalue 0.1. We choose *π* = 0.08 for *n* = 1000, and *π* = 0.02 for *n* = 80.

#### C.1. Truncated GGMs

In this section we present the results for truncated GGMs.

##### C.1.1. Choice of *h*

The results for truncated centered GGMs are reported in Table 4 and Figure 13. Those for truncated non-centered GGMs using the profiled estimator are in Table 5 and Figure 14.

##### C.1.2. Choice of multiplier

The results for truncated centered GGMs where each curve represents a different multiplier are shown in Figure 15, and those for truncated non-centered GGMs are in Figure 16, where each curve corresponds to a different ratio *λ*_**K**_/*λ*_***η***_.

#### C.2. Other *a/b* Models

Figure 17 exhibits the results for the exponential models.

Figure 18 displays the results for the gamma models.

Figures 19 and 20 demonstrate the results for *a* = 3/2 and *b* = 1/2 or *b* = 0, respectively.
